# Mechanisms of Chromosome Congression during Mitosis

**DOI:** 10.3390/biology6010013

**Published:** 2017-02-17

**Authors:** Helder Maiato, Ana Margarida Gomes, Filipe Sousa, Marin Barisic

**Affiliations:** 1 Chromosome Instability & Dynamics Laboratory, Instituto de Biologia Molecular e Celular, Universidade do Porto, Rua Alfredo Allen 208, 4200-135 Porto, Portugal; margarida.gomes@ibmc.up.pt (A.M.G.); filipe.sousa@ibmc.up.pt (F.S.); 2 Instituto de Investigação e Inovação em Saúde—i3S, Universidade do Porto, Rua Alfredo Allen 208, 4200-135 Porto, Portugal; 3 Cell Division Group, Experimental Biology Unit, Department of Biomedicine, Faculdade de Medicina, Universidade do Porto, Alameda Prof. Hernâni Monteiro, 4200-319 Porto, Portugal; 4 Danish Cancer Society Research Center, Cell Division Laboratory, Strandboulevarden 49, 2100 Copenhagen, Denmark; barisic@cancer.dk

**Keywords:** mitosis, microtubule, kinetochore, mitotic spindle, polar ejection forces, Kinesin, Dynein, CENP-E, Chromokinesin, chromosome, tubulin code

## Abstract

Chromosome congression during prometaphase culminates with the establishment of a metaphase plate, a hallmark of mitosis in metazoans. Classical views resulting from more than 100 years of research on this topic have attempted to explain chromosome congression based on the balance between opposing pulling and/or pushing forces that reach an equilibrium near the spindle equator. However, in mammalian cells, chromosome bi-orientation and force balance at kinetochores are not required for chromosome congression, whereas the mechanisms of chromosome congression are not necessarily involved in the maintenance of chromosome alignment after congression. Thus, chromosome congression and maintenance of alignment are determined by different principles. Moreover, it is now clear that not all chromosomes use the same mechanism for congressing to the spindle equator. Those chromosomes that are favorably positioned between both poles when the nuclear envelope breaks down use the so-called “direct congression” pathway in which chromosomes align after bi-orientation and the establishment of end-on kinetochore-microtubule attachments. This favors the balanced action of kinetochore pulling forces and polar ejection forces along chromosome arms that drive chromosome oscillatory movements during and after congression. The other pathway, which we call “peripheral congression”, is independent of end-on kinetochore microtubule-attachments and relies on the dominant and coordinated action of the kinetochore motors Dynein and Centromere Protein E (CENP-E) that mediate the lateral transport of peripheral chromosomes along microtubules, first towards the poles and subsequently towards the equator. How the opposite polarities of kinetochore motors are regulated in space and time to drive congression of peripheral chromosomes only now starts to be understood. This appears to be regulated by position-dependent phosphorylation of both Dynein and CENP-E and by spindle microtubule diversity by means of tubulin post-translational modifications. This so-called “tubulin code” might work as a navigation system that selectively guides kinetochore motors with opposite polarities along specific spindle microtubule populations, ultimately leading to the congression of peripheral chromosomes. We propose an integrated model of chromosome congression in mammalian cells that depends essentially on the following parameters: (1) chromosome position relative to the spindle poles after nuclear envelope breakdown; (2) establishment of stable end-on kinetochore-microtubule attachments and bi-orientation; (3) coordination between kinetochore- and arm-associated motors; and (4) spatial signatures associated with post-translational modifications of specific spindle microtubule populations. The physiological consequences of abnormal chromosome congression, as well as the therapeutic potential of inhibiting chromosome congression are also discussed.

## 1. Introduction

### 1.1. What is Chromosome Congression?

In preparation for cell division, two poles and an equator start to be defined by the mitotic spindle axis. Precisely at the onset of mitosis, when chromosomes start condensing and the nuclear envelope breaks down, dispersed chromosomes initiate directed movements that culminate with their position at the spindle equator before migrating to the poles after sister chromatid separation. This stochastic motion towards the equator coincides with the beginning of prometaphase and is known as “chromosome congression” (from the English “to come together”; terminology first introduced by Darlington [1]). Chromosome congression truly represents the first challenge of mitosis and culminates with the formation of a metaphase plate, a hallmark of mitosis in metazoans, and occurs in tight spatiotemporal coordination with the assembly of the mitotic spindle that mediates the microtubule-chromosome interactions required for chromosome movement.

### 1.2. Why do Chromosomes Congress?

At first glance, it may seem counterintuitive that before chromosomes segregate to the poles (during anaphase), they first meet at the equator. This likely reflects millions of years of evolution aiming to improve chromosome segregation fidelity. For instance, if one imagines a mitotic cell in which chromosomes do not congress, the risk of chromosome missegregation after sister chromatid separation at anaphase would be too high, unless all chromatids are extensively moved apart, like in the budding yeast S. cerevisiae, in which the anaphase spindle elongates about 5-fold relative to the metaphase spindle length [2]. In contrast, metazoan spindles only elongate less than 2-fold the metaphase spindle length [3] and thus must rely on different strategies to ensure faithful chromosome segregation during anaphase. One of these strategies is precisely the formation of a metaphase plate, forcing all chromosomes to start subsequent poleward motion from the same position relative to the spindle axis, i.e., from the equator. The other is to trigger an abrupt cleavage of cohesin by separase-mediated degradation of securin, leading to the synchronous separation and movement of sister chromatids towards the pole. This anaphase synchrony has been shown to depend on the uniform distribution of spindle forces acting on all chromosomes prior to anaphase [4]. Aligning chromosomes at the equator also maximizes the chances of kinetochore capture by microtubules emanating from both spindle poles leading to chromosome bi-orientation, which is required to satisfy the spindle assembly checkpoint (SAC; see [5]). Finally, chromosome congression is important to prevent unstable/erroneous kinetochore-microtubule attachments because the proximity to the poles promotes microtubule destabilization at kinetochores due to high Aurora A kinase activity that leads to phosphorylation of Ndc80 (among others), thereby reducing its affinity for microtubules [6,7,8]. In addition, tension generated by opposing pulling forces on aligned bi-oriented chromosomes is required and sufficient to stabilize correct attachments [9].

## 2. Mechanisms of Chromosome Congression

### 2.1. Historical Perspective

In contrast to many other fundamental concepts behind cell division, if one looks for references to the problem of chromosome congression in the early compilations about “The Cell” by E. B. Wilson at the turn of the 20th century, one finds a huge gap in knowledge between the so-called “prophases”, which dealt essentially with the condensation and resolution of visible threads/chromatids, and metaphase, by which chromosomes already lie at the equator. The very few references to what happens between these two stages can be resumed in a single sentence: “After definite formation of the chromosomes the nuclear membrane usually disappears and the chromosomes (…) are set free in the protoplasm (and) take up their position in the equatorial plane of the spindle” [10]. Most of the attention at that time was focused on the mechanisms of anaphase and, due to the lack of live-cell studies, the longest stage of mitosis in vertebrates that comprises the entire prometaphase (a term that was only later introduced by Lawrence [11]) was completely left out of the equation.

**Figure 1:**
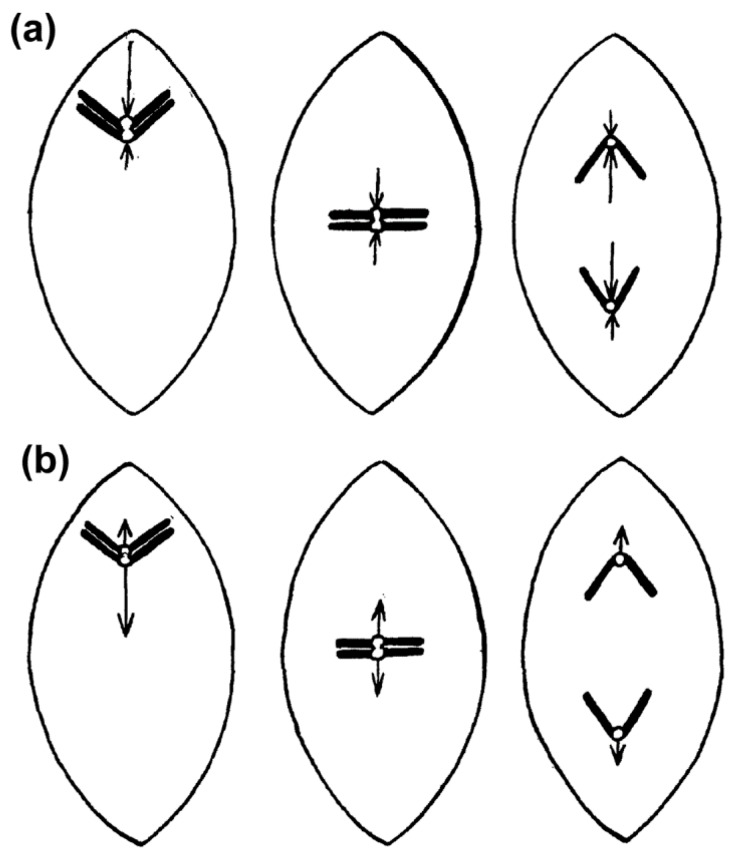
First models of chromosome congression involving either pushing or pulling forces on chromosomes. (**a**) Model of chromosome congression proposed by Darlington [1] involving a balance of pushing forces on chromosomes. These forces are higher when chromosomes are closer to spindle poles; (**b**) Model of chromosome congression proposed by Östergren involving pulling forces on chromosomes that are proportional to k-fiber length. Adapted from Östergren, 1950 [13] and displayed under a Creative Commons Attribution-Noncommercial-Share Alike 4.0 International license, as described at https://creativecommons.org/licenses/by-nc-sa/4.0/legalcode.

The first ideas that attempted to explain the process of chromosome congression date back to 1895 from the works of Drüner [12], and later further developed in the works of Belar, Darlington, Rashevsky, Wada and Östergren [1,13,14,15,16,17] (reviewed in [18]). These models conceived that chromosomes are either repelled from the pole by a pushing force that decreases with distance, or attracted to the pole by a pulling force that increases with distance, until all chromosomes eventually reach an equilibrium condition at the equator (Figure 1). One key conceptual difference between these models was the assumption (by some authors) of the existence of kinetochore-to-pole connections from the very beginning of prometaphase. For instance, Belar conceived unaligned chromosomes attached to a “traction fiber” sliding along continuous fibers (most likely interpolar microtubules, as we know them today) until chromosomes eventually reach the equator. However, it was unclear whether bi-orientation and the formation of effective kinetochore-microtubule attachments that connect unaligned chromosomes with the poles was required for initial chromosome congression towards the equator. Moreover, it had been naively assumed that the mechanisms required for initial chromosome congression also play a role in maintaining the equatorial position of chromosomes (see Section 2.10). This is particularly evident in the model proposed by Östergren, who explained chromosome congression by a model in which pulling forces on a given kinetochore act as a linear function of kinetochore-fiber (k-fiber) length. Östergren based his arguments on work with naturally occurring trivalents during meiosis I that were often found positioned off the equator, with their two-kinetochore side closer to the pole, based on the assumption that the pulling force on two kinetochores is higher than on single kinetochores [17,19].

**Figure 2:**
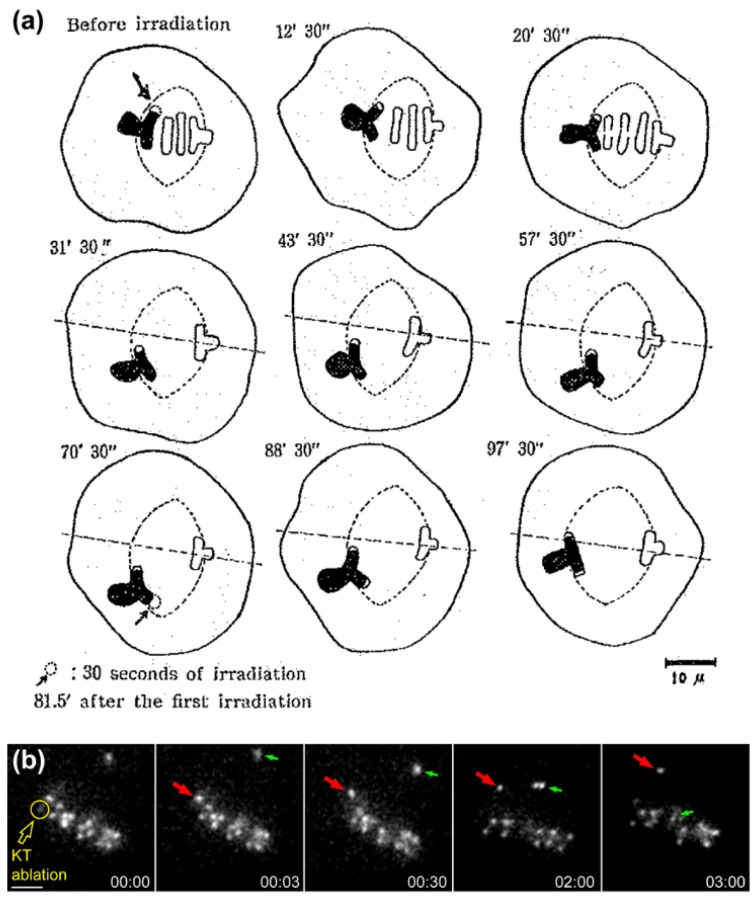
Evidence that forces on kinetochores are required to position chromosomes at the equator. (**a**) Original drawings from Izutzu depicting the loss of equatorial position when one of the kinetochore regions from a bivalent chromosome was irradiated with an UV microbeam. Note the displacement of the bivalent from the metaphase plate towards the pole facing the non-irradiated kinetochore after irradiation. Scale bar is 10 μm. Reprinted from Izutsu et al., 1959 [20]; (**b**) Laser microsurgery of one of the kinetochores from an equatorially-aligned chromosome in a *Drosophila* S2 cell. Kinetochores were directly labelled with the Centromere Protein A (CENP-A) homologue Cid fused with Green Fluorescent Protein (GFP). Likewise, the chromosome was displaced from the equator after surgery and underwent poleward migration towards the pole facing the undisturbed kinetochore from the pair. Red arrows track the undisturbed kinetochore from the irradiated pair. Green arrows track the congression of an undisturbed chromosome. Laser microsurgery was performed as described in [29]. Scale bar is 2 μm.

Direct evidence that the equatorial position of (already aligned) chromosomes is determined by antagonistic pulling forces on opposing kinetochores was provided by the works of Izutsu and colleagues. They irradiated one kinetochore region of a grasshopper bivalent chromosome in metaphase I using a focused UV microbeam, resulting in the gradual motion of the irradiated bivalent towards the spindle pole facing the non-irradiated kinetochore [20,21,22] (Figure 2a). Similar findings were subsequently reported by McNeal and Berns for mitotic chromosomes in cultured PtK2 cells [23] (see Figure 2b for a representative example using *Drosophila* S2 cells). Hays and colleagues also estimated the force-length relationship on experimentally generated trivalents in living grasshopper spermatocytes and found it to be consistent with Östergren’s hypothesis [24]. However, ideas that the pulling force on kinetochores is not a function of k-fiber length, but rather of their diameter (as function of the number of microtubules attached) started to emerge [25], but even this view has been controversial. For instance, a balance of microtubule numbers on opposite kinetochores has been suggested by elegant experiments using laser microsurgery combined with correlative light and electron microscopy of meiosis I spermatocytes [26], but recent work that measured birefringence retardation of k-fibers of maloriented bivalents challenged this model [27]. In addition, no positive correlation between the number of kinetochore microtubules and the direction of chromosome movement could be observed in vertebrate cells [28]. Overall, these pioneering studies provided definitive demonstration that chromosome position at the equator is maintained (but not necessarily achieved) through a balance of pulling forces acting on opposite kinetochores from the same chromosome that do not strictly depend on k-fiber length or kinetochore microtubule number.

### 2.2. Polar Ejection Forces

Several subsequent works have challenged aspects of Östergren’s hypothesis based on the prediction that kinetochore-pulling forces depend on k-fiber length. If that were the case, one would expect that severing a k-fiber on a metaphase chromosome should lead to a significant displacement of the aligned chromosome towards the pole facing the undamaged k-fiber. However, several experiments that aimed to cut through k-fibers in different systems (from plant to human cells in culture) have revealed that chromosomes either do not shift at all or shift only slightly towards the pole of the unperturbed k-fiber [21,22,29,30,31,32,33,34,35,36,37,38].

Important observations that shed light on the mechanism of chromosome congression came from studies of chromosome behavior during transient monopolar spindle formation in newt cells by Bajer and Mole-Bajer. They astutely noticed that “…the chromosomes approached the pole only up to a certain distance and it was evident that they could not come closer to the pole.” [39]. These observations further challenged Östergren’s hypothesis based exclusively on pulling forces acting on kinetochores from the same chromosome, as it would have been predicted that a mono-oriented chromosome would travel all the way to the pole, which was not the case. Overall, these data indicate that although kinetochore pulling forces are important to position chromosomes at the equator, as proposed by Östergren, their magnitude is independent of k-fiber length, implying the existence of additional mechanisms.

**Figure 3:**
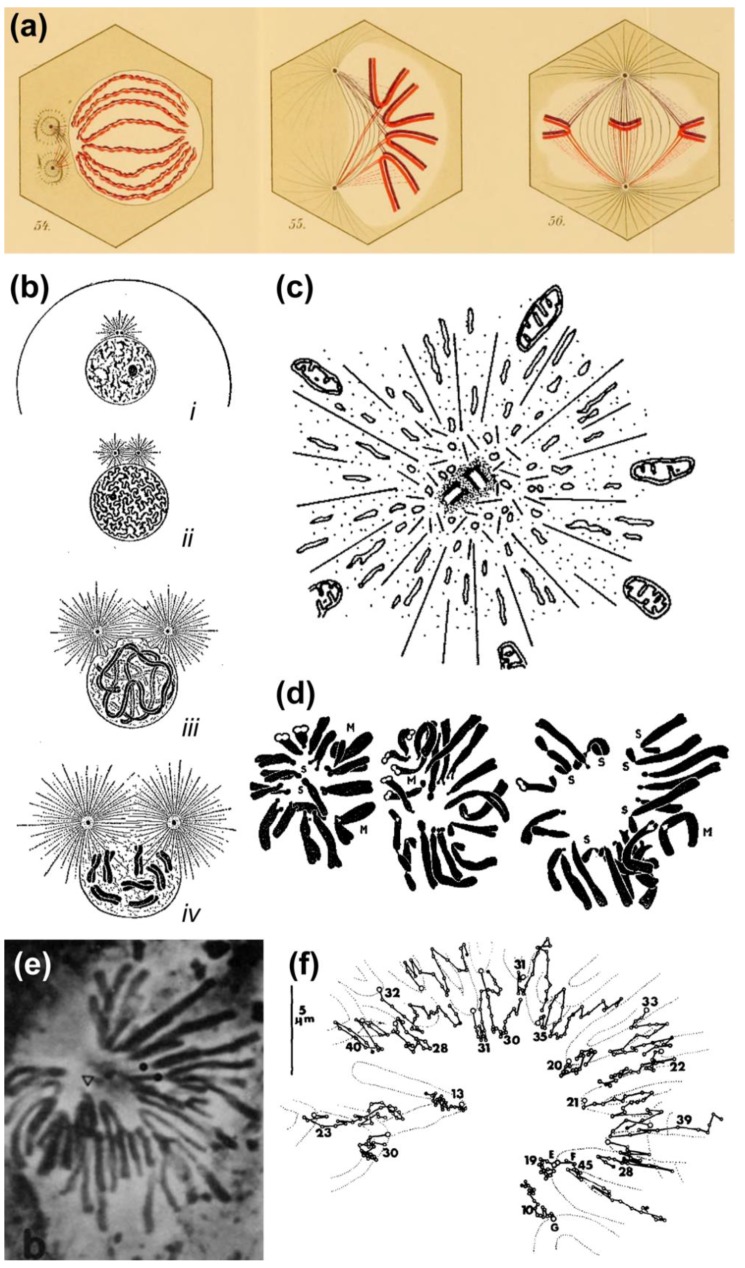
Evidence that centrosome-derived microtubules can exert pushing forces. (**a**) Original Drawings by Drüner depicting the invasion of the chromosomal region by microtubules, which exert a pushing force that assists chromosome alignment at the spindle equator. Reprinted from Drüner, 1895 [12]. Image courtesy of Biodiversity Heritage Library. http://www.biodiversitylibrary.org; (**b**) Schematic drawing by E. B. Wilson illustrating the pushing action of centrosomal microtubules on the nuclear envelope and subsequent rupture. Reprinted from Wilson, 1925 [10]. Image displayed under a Creative Commons Attribution-Noncommercial-Share Alike 4.0 International license, as described at https://creativecommons.org/licenses/by-nc-sa/4.0/legalcode. Image courtesy of the Wellcome Library. http://wellcomelibrary.org; (**c**) Schematic drawing by Luykx illustrating the repulsive action of centrosomal microtubules over large organelles (mitochondria). Reprinted from Luykx, 1970 [40]. Courtesy of Elsevier; (**d**) Original drawings by Darlington illustrating the variability in chromosome positioning in pollen grain cells. Reprinted from Darlington, 1937 [1]. Image courtesy of Biodiversity Heritage Library. http://www.biodiversitylibrary.org; (**e**,**f**) Phase contrast image of a newt lung cell undergoing transient monopolar configuration. Kinetochore position was tracked over time, clearly demonstrating the oscillatory behavior of mono-oriented chromosomes in this system. Note that chromosomes do not travel all the way towards the pole. Reprinted from Bajer et al., 1982 [41] and displayed under a Creative Commons Attribution-Noncommercial-Share Alike 4.0 International license, as described at https://creativecommons.org/licenses/by-nc-sa/4.0/legalcode.

Based on their observations on transient monopolar spindles, Bajer and Mole-Bajer proposed that “The only logical explanation for the behavior of chromosomes in monopolar division is that the chromosomes approach the center of the aster only to the point at which there is equilibrium between the aster elimination property and the pulling of kinetochore fibers.” [39]. Although this “aster elimination property” or “polar ejection force (PEF)” has been noted more than a century ago by Drüner, who refers to a pressure by “growing beams” [i.e., microtubules] from the poles when they encounter an obstacle such as chromosomes [12] (Figure 3a), and was quite evident in the invaginations of the nuclear envelope as the aster develops in prophase (see [10]; Figure 3b) and found to exclude large organelles (e.g., mitochondria) from the centriolar region (reviewed in [40]; Figure 3c), it was Darlington that firmly proposed its involvement in chromosome congression (although he assumed this was essentially due to electrostatic repulsions). This view was based on the analysis of pollen-grain mitosis, in which the distance of peripheral centromeres relative to the spindle pole was highly variable [1] (Figure 3d). This irregular pattern likely reflected the dynamic behavior of chromosomes on monopolar spindles, which was subsequently extensively characterized by Bajer and colleagues [39,41,42] (Figure 3e,f). Together, these studies supported a new view of chromosome congression involving a balance of PEFs and kinetochore-pulling forces.

**Figure 4:**
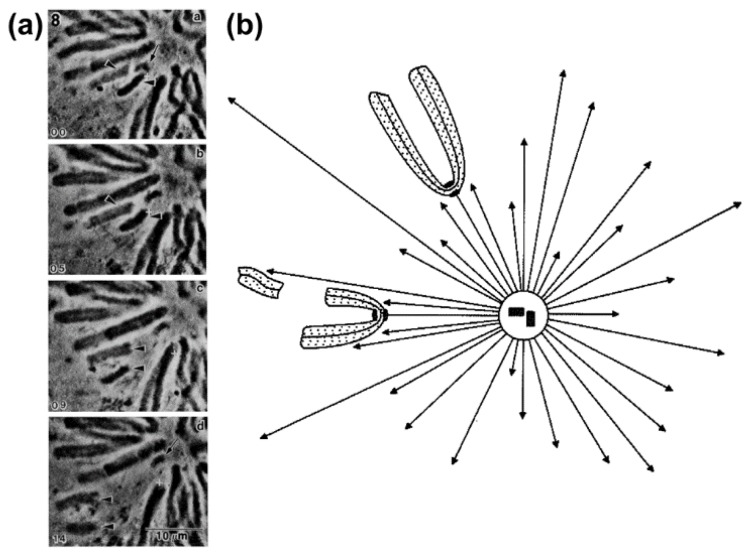
Demonstration that polar ejection forces act along the entire chromosome. (**a**) Phase contrast image of a newt lung cell in which the chromosome arms on one chromosome (arrowheads) were physically separated from the kinetochore region using laser microsurgery. Note the ejection of the acentric chromosome arms away from the polar region. In contrast, the kinetochore-containing region (arrow) moves closer to the polar region. Reprinted from Rieder et al., 1986 [43] and displayed under a Creative Commons Attribution-Noncommercial-Share Alike 4.0 International license, as described at https://creativecommons.org/licenses/by-nc-sa/4.0/legalcode; (**b**) Schematic representation of the experiment illustrated in (a). Reprinted from Salmon, 1989 [44]. Courtesy of Elsevier.

The exact nature and mode of action of PEFs was only elucidated by Rieder and colleagues using an elegant combination of laser microsurgery and correlative light-electron microscopy experiments [43]. First, they demonstrated that the distal kinetochore from an oscillating mono-oriented chromosome was indeed devoid of microtubules and consequently was not under opposing kinetochore pulling forces. Second, by cutting near the kinetochore regions of mono-oriented chromosomes to generate acentric fragments (i.e., without kinetochore), they found that kinetochore-free chromosome arms were immediately ejected away from the spindle pole with velocities similar to the outward movement of an oscillating chromosome [44], whereas the remaining kinetochore-containing fragment moved closer to the pole [43] (see also [44,45]; Figure 4a,b). Subsequent studies by Salmon, Rieder and colleagues have further demonstrated that when astral microtubules were reversibly depolymerized/polymerized, mono-oriented chromosomes moved closer to or were pushed away from the pole, respectively [44,46,47]. These studies revealed no difference in the mechanism of chromosome positioning between monopolar and bipolar spindles, including average distances from the pole. Finally, it was shown that kinetochores moving away from their associated pole do not exert a significant pushing force on the chromosome [48,49] and PEFs determine the amplitude of chromosome oscillations near the pole [50]. Thus, PEFs derived from astral microtubules acting along chromosome arms oppose kinetochore-pulling forces. This “push-pull” mechanism was proposed to account for chromosome oscillations, while determining chromosome position relative to the spindle pole. In the context of a bipolar spindle, chromosome congression could now be explained in light of the balance of four forces on a chromosome: two antagonistic poleward forces acting at the kinetochores and two opposing PEFs acting along chromosome arms. As so, formation of a metaphase plate equidistant to the spindle poles would result from the net forces applied to the chromosomes being zero [44,47]. An integrated view of these studies can be found in a landmark essay that firmly established the contribution of PEFs and kinetochore directional instability (i.e., kinetochores can switch from poleward to anti-poleward motion) for chromosome congression in vertebrates [51].

PEFs are likely associated with the pushing action of elongating astral microtubules undergoing dynamic instability along the length of the chromosome. Consistent with this idea, taxol-induced polymerization of polar microtubules can push chromosome arms away from the pole [45,52], whereas nocodazole or colcemid treatment completely abolished PEFs on chromosomes [45]. Importantly, dynamic microtubules were shown to be required for continuous ejection of chromosome arms away from the poles [45]. Theoretical predictions and calculation of PEFs distribution further indicate that PEFs are stronger closer to the center of the aster, where microtubule density is higher, and depend on chromosome size [47,50]. In vitro measurements of the force produced by a polymerizing microtubule against a rigid surface or inside lipid vesicles have determined maximal forces between 2–4 pN [53,54]. Interestingly, it was found that forces on short buckling microtubules tend to be higher than those on long buckling microtubules, likely reflecting the length-dependent stiffness of microtubules [53]. Attempts to measure the scale of PEFs by individual microtubules on chromosomes using either in vitro reconstitution or in vivo systems have estimated a force between 0.5–1 pN per microtubule [55,56] and ~100 pN near the pole where microtubule density is higher [56]. While the PEF produced by individual microtubules is compatible with that generated by polymerizing microtubules in vitro [53,54], it was also consistent with the force generated by single Kinesin motors [57,58,59], suggesting their involvement in PEFs [44,60].

### 2.3. The Role of Chromosome Arm-Associated Motors in the Generation of Polar Ejection Forces

Chromokinesins are Kinesin-like motor proteins that have DNA-binding properties and associate with chromosomes during mitosis [61,62]. The best characterized mammalian Chromokinesins are Kif4A and Kid, which belong to two distinct families: Kinesin-4 and Kinesin-10, respectively (reviewed in [63]). Functional analysis revealed a combined role for Kinesin-4 and Kinesin-10 in chromosome congression, arm-orientation and normal chromosome oscillations, consistent with an active role of Kinesin-4 and Kinesin-10 in the generation of PEFs [6,62,64,65,66,67,68,69,70,71,72]. Both Kinesin-4 and Kinesin-10 were shown to have microtubule plus-end directed motility [73,74,75], but they appeared to be non- or weakly-processive motors under load [74,75]. Nevertheless, antibody-blocking experiments in vitro suggested that Kinesin-10 is a major contributor for PEFs [56]. In vitro reconstitution experiments have indicated that, despite of its slower motility compared to Kinesin-4 [74,75], Kinesin-10 binds more strongly to microtubules and dominates over Kinesin-4 during cooperative microtubule motility associated with chromatin [76]. Similar findings have been reported upon functional perturbation of these two Chromokinesin families in *Drosophila* and human cells, which suggested a combined role during chromosome congression, with Kinesin-10 providing the major PEF required for arm orientation and Kinesin-4 mainly regulating microtubule dynamics [68,71]. Altogether, these data can be reconciled in light of the “soft” nature of the chromosomes. If strong and highly processive motors worked as PEF generators, this would likely lead to chromatin deformations/damage and loss of chromosome structure. Indeed, overexpression of Kinesin-10 in *Drosophila* S2 cells was shown to stretch and deform chromatin when microtubules impact or pass by the chromosomes [77]. As discussed by Brouhard and Hunt for Kinesin-10 [56], the combined action of Kinesin-10 and Kinesin-4 on chromosome arms is ideal for exerting PEFs against microtubules through slow, weak, and discontinuous action, which would be sufficient to bias chromosome ejection away from the poles without inducing damage. Finally, direct demonstration that Kinesin-4 and Kinesin-10 collectively mediate PEFs on chromosome arms in human cells was only recently obtained. By combining RNAi-mediated depletion of Kid and Kif4A with laser microsurgery to generate acentric chromosome fragments in human culture cells, it was shown that arm ejection forces operating in the absence of kinetochore-pulling forces relied on the cooperative action between Kinesin-4 and Kinesin-10, with only a minor fraction that could be attributed to the pushing force of polymerizing microtubules impacting on chromosome arms [6]. Most importantly, this work revealed that PEFs operating on acentric fragments caused the ejection of chromosome arms in random directions, including towards the cortex. This indicated that although PEFs mediated by Kinesin-4 and Kinesin-10 are sufficient to exert a pushing force on chromosome arms that leads to chromosome ejection away from the pole, they are not the critical players that conduct chromosome movement exclusively towards the equator.

A critical aspect of the model proposed by Rieder and Salmon was that congressing mono-oriented chromosomes experience tension at kinetochores as result from the push-pull between PEFs along chromosome arms and kinetochore-pulling forces [51,78]. This was a reasonable assumption based on findings that mono-oriented chromosomes during transient monopolar formation in newt cells showed robust k-fibers on the attached kinetochore [43,46]. However, kinetochore-microtubule attachments on mono-oriented chromosomes are highly unstable, unless constant tension away from the pole is applied [9,79,80] (see also [47]). This apparent paradox could only be solved if PEFs produce sufficient kinetochore tension independently of opposing kinetochore-pulling forces that result from chromosome bi-orientation. This hypothesis has been recently tested in *Drosophila* culture cells. Elegant experiments involving overexpression of Kinesin-10 have first indicated that elevated PEFs could indeed stabilize kinetochore-microtubule attachments [77]. These proof-of-concept experiments were followed by studies of *Drosophila* cultured cells undergoing mitosis with unreplicated genomes (MUGs), where the function of individual kinetochores could be investigated in the context of single chromatids that are unable to bi-orient [81]. In this work it was shown that PEFs mediated by Kinesin-4 and Kinesin-10 stabilize kinetochore-microtubule attachments on mono-oriented chromosomes. Over time, mono-oriented chromosomes were also shown to experience significant intra-kinetochore stretch or structural deformation (see discussion in [82,83,84]) comparable with those typically experienced by bi-oriented chromosomes [81]. Taken together, these data indicate that Chromokinesin-mediated PEFs oppose kinetochore-pulling forces and contribute to tension-dependent stabilization of microtubule attachments on mono-oriented chromosomes. 

### 2.4. Coordination between PEFs and Kinetochore-Pulling Forces Drives Chromosome Congression after Bi-Orientation

A related problem that derives from the existence of kinetochore-pulling forces on attached chromosomes concerns their nature. One model is based on the action of pulling forces resulting from depolymerization of attached kinetochore microtubules. This model stems from original work by Shinya Inoue on the effect of colchicine on spindle microtubules and chromosome movement using oocytes from the marine annelid worm *Chaetopterus pergamentaceous*. In this system, the metaphase arrested spindle is anchored by one of its poles to the cell cortex and, upon addition of colchicine or cold treatment (now well established treatments that induce spindle microtubule depolymerization), the aligned chromosomes at the spindle equator were observed to move towards the anchored pole [31,85]. Based on these observations, Inoue concluded that the spindle affected by colchicine or cold is able to perform mechanical work and exert a pulling force on chromosomes (reviewed in [86]). In vitro reconstitution works have provided additional evidence that microtubule depolymerization at their plus-ends can exert a pulling force on the kinetochore that is independent of ATP hydrolysis and is sufficient to move chromosomes [87,88,89]. In agreement, nocodazole-induced microtubule depolymerization has been shown to occur near the kinetochore during poleward chromosome movement in prometaphase [90]. Moreover, oscillating mono-oriented chromosomes have been proposed to switch from microtubule depolymerization and polymerization states, as inferred by accumulation of EB proteins at growing microtubule plus-ends at kinetochores [91]. However, based on the analysis of the profile of individual microtubule plus-ends within a k-fiber, it has been proposed that two-thirds adopt a conformation compatible with a microtubule depolymerizing state, regardless of the directional instability associated with poleward and anti-poleward chromosome oscillations [92]. These apparently contradicting findings have recently been reconciled by the observation that EB protein bursts near kinetochores are rather infrequent and only represent a small bias for microtubule polymerization within an incoherent k-fiber that contains a mixture of polymerizing and depolymerizing microtubules [93]. Overall, these data support a model in which regulation of microtubule dynamics favoring depolymerization can generate pulling forces on attached kinetochores.

Any model of chromosome congression involving kinetochore-pulling forces implies that any perturbation of end-on kinetochore-microtubule attachments or defects in spindle assembly/organization would lead to chromosome alignment problems. Indeed, an extensive survey of the literature revealed more than 100 proteins that have been implicated in chromosome alignment (Table 1), yet it is only for a select handful that we know the mechanism and thus will represent our focus in this review. Probably the best studied case is the one involving the KMN network, which forms the core microtubule interface at kinetochores and all respective regulatory proteins, such as Aurora B and Plk1 kinases (reviewed in [94]). Additionally, proteins that modulate kinetochore-microtubule attachments and their dynamic state are also likely to play an important role. Among these, microtubule plus-end-tracking proteins (+TIPs) are of special interest due to their specific accumulation at the plus-ends of microtubules [95,96,97] where they promote microtubule growth by catalyzing the addition of tubulin subunits to microtubule plus-ends [98], by inducing rescue [99], or by stabilizing microtubules [100,101]. CLIP-170 was the first +TIP reported [102] and was initially associated with microtubule rescue [99]. Functional inhibition of CLIP-170 during mitosis results in chromosome alignment defects, possibly associated with defective kinetochore-microtubule attachments [103,104]. However, CLIP-170 inhibition does not seem to affect kinetochore microtubule dynamics or stability, possibly because it is stripped from the kinetochore by Dynein upon the establishment of end-on kinetochore-microtubule attachments [103,104]. Moreover, phosphorylation of CLIP-170 at S312 by Plk1 regulates its binding to microtubules and is crucial for chromosome alignment [105]. CLIP-170 appears to promote kinetochore-microtubule attachments and chromosome congression by counteracting Dynein/Dynactin [106]. The XMAP215/Ch-TOG and CLASP families of +TIPs have also been implicated in chromosome congression. The XMAP215/Ch-TOG proteins act as microtubule polymerases at microtubule plus-ends and promote microtubule assembly [98,107,108], whereas CLASPs promote microtubule rescue and suppress catastrophe [109,110]. Depletion of proteins from the XMAP215/Ch-TOG family results in the presence of unattached kinetochores and chromosome alignment defects [111,112,113,114]. Moreover, XMAP215/Ch-TOG contributes to chromosome oscillations [115]. Recruitment of CLASPs to microtubule plus-ends requires interactions with CLIP-170 and EB1 [100,101]. Importantly, CLASPs also localize to kinetochores in a microtubule-independent manner and remain at kinetochores upon microtubule attachment [116,117]. This localization at the kinetochore-microtubule interface favors a role of CLASPs in the regulation of microtubule dynamics at the kinetochore [118,119], thereby contributing for chromosome congression [116]. Surprisingly, perturbation of either CLASPs or XMAP215/Ch-TOG increases the stability of kinetochore-microtubule attachments [115,119]. One possibility might be that during mitosis the activity of these proteins is regulated by phosphorylation and/or binding to other proteins that promote microtubule depolymerization [120,121].

The members of the Kinesin-13 family Kif2a, Kif2b and Kif2c/MCAK are also important regulators of microtubule dynamics, including at kinetochores [122]. Kinesin-13 proteins are non-motile but use the energy from ATP hydrolysis to promote microtubule depolymerization by binding both the plus- and the minus-ends of microtubules and inducing a conformational change that leads to a catastrophe event [123,124,125]. In the context of the mitotic spindle, Kinesin-13 proteins associate with both spindle poles and kinetochores where they play distinct roles [124,126]. Kif2b and MCAK regulate microtubule plus-end dynamics at the kinetochore where they play an important role in the correction of erroneous microtubule attachments [124,127,128,129,130], while Kif2a appears to have a preference for microtubule minus-ends where it plays an important role in the regulation of spindle microtubule flux [131,132]. Interestingly, Kif2a and MCAK are dispensable for chromosome congression [132], whereas Kif2b appears to be required for proper chromosome oscillation on a monopolar spindle configuration [124]. However, because Kif2b only transiently associates with kinetochores before microtubule attachments [124] it is unlikely to play an important role assisting chromosome congression after bi-orientation, suggesting the involvement of other players. 

The widely conserved Kinesin-8 family has been proposed to function both as plus-end-directed motors and as microtubule depolymerases [133,134,135,136]. However, the depolymerase activity of human Kif18A remains controversial. Although Kif18A was initially proposed as a microtubule depolymerase [134], further studies suggested that Kif18A suppresses microtubule growth by capping the microtubule plus-ends [137,138]. This would be consistent with the emerging role of Kinesin-8 motors as negative regulators of microtubule length, since loss of Kinesin-8 activity generally leads to longer cellular microtubules [134,139,140,141,142]. Importantly, genetic and siRNA-based studies demonstrate that Kinesin-8 motors are necessary for proper chromosome alignment by suppressing chromosome oscillations on bi-oriented chromosomes [68,70,134,139,143,144]. Accordingly, in the absence of functional Kif18A, kinetochores exhibit an increase in the oscillation amplitude leading to a deregulation of metaphase plate organization [144]. Furthermore, loss of Kif18A leads to a modest increase in spindle size and longer microtubules [134,144]. In agreement, overexpression of Kif18A decreases chromosome oscillations, favoring chromosome alignment at the metaphase plate [144,145]. Overall, these data are consistent with a model of chromosome congression after bi-orientation, in which Kif18A forms a gradient along attached kinetochore microtubules, directly regulating their length and dynamics to facilitate chromosome alignment at the spindle equator [144].

The co-existence of PEFs acting along the entire chromosome arms and kinetochore-pulling forces driven by microtubule depolymerization suggests that they might work in parallel to regulate chromosome oscillations during congression after bi-orientation. Disruption of PEFs by inhibition of Chromokinesin function in cultured cells altered chromosome oscillations on both monopolar and bipolar spindles [65,66,67,71]. Although perturbation of Chromokinesin functions did not fully compromise chromosome congression, few monooriented chromosomes remained close to the poles, suggesting that PEFs might increase the efficiency of chromosome congression by facilitating the stabilization of end-on kinetochore microtubule attachments and biorientation [77,81]. Furthermore, despite having opposite effects on chromosome movement, PEFs and Kif18A synergistically promote the position of bi-oriented chromosomes near the spindle equator [146]. Overall, these findings suggest that the coordinated activities of Kif18A and PEFs regulate chromosome oscillations and are important for chromosome congression after bi-orientation.

### 2.5. The Role of Kinetochore Motors in Chromosome Congression

A concurrent model for the explanation of kinetochore-pulling forces is based on the presence of ATP-dependent motor proteins at kinetochores. The best candidate for such force generator is the cytoplasmic form of the microtubule minus-end-directed motor Dynein, which has been shown to localize to kinetochores [268,269] and was proposed to counteract the action of PEFs on chromosome arms by generating kinetochore poleward motion [51]. However, despite some evidence (mostly from studies in anaphase) supporting a requirement for kinetochore Dynein in chromosome poleward motion, this remains a highly controversial issue (reviewed in [270]). The strongest arguments against such a role are based on the fact that chromosome-to-pole velocities in anaphase are about one order of magnitude slower than those typically observed by Dynein-dependent transport and Dynein accumulation at kinetochores is negatively regulated by microtubule attachments [271,272]. Moreover, inhibition of Dynein motor activity did not affect minus-end-directed chromosome motion driven by microtubule depolymerization in vitro [88,273,274]. Although it remains possible that few molecules of Dynein are able to generate kinetochore-pulling forces after the establishment of end-on microtubule attachments during chromosome congression, the rate of motion is likely governed by other processes, such as microtubule depolymerization.

Although a major role played by kinetochore Dynein in the generation of kinetochore-pulling forces after the establishment of end-on microtubule attachments is disputable, its role in the stages that precede chromosome congression is well supported. It has long been noticed by Schneider that some chromosomes tend to move toward the poles before congressing to the spindle equator [275]. Bajer and Mole-Bajer, in their classic cinematographic studies of mitosis also clearly demonstrate and recognize that some chromosomes undergo poleward motion before migrating to the equator [276,277]. Similar findings have been reported in cultured newt cells by Zirkle and colleagues, who first recognized the frequent appearance of “centrophilic” chromosomes (i.e., that lie near the centrosomes) that do not migrate straightaway to the equator [278,279,280], as well as in insect spermatocytes [281] and PtK1 cells [282]. These sharp observations have indicated that the process of chromosome congression is complex and that not all chromosomes follow the same path, suggesting the existence of concurrent mechanisms.

The implication of Dynein in the poleward movement of chromosomes that precede congression of some chromosomes was proposed even a few months before the report of its localization to kinetochores [268,269], based on the characterization of initial kinetochore-microtubule interactions during early prometaphase [283]. This study showed that a single astral microtubule extending well beyond the kinetochore region was sufficient to mediate the initial attachment and subsequent poleward movement of some chromosomes. Importantly, this association involved the tangential interaction between the microtubule and the kinetochore fibrous corona (the outermost domain of the kinetochore that expands into crescents in the absence of attached microtubules) and was independent of microtubule depolymerization. Based on the recorded velocities of chromosomes during this fast poleward movement after initial lateral interaction between kinetochores and microtubules (typically ranging between 25–55 μm/min in newt lung cells in culture), Rieder and Alexander proposed that Dynein at kinetochores could account for this behavior. This proposal was seconded by Merdes and De Mey (after the discovery of Dynein at kinetochores) who reported similar findings [284]. Shortly thereafter, it was shown that kinetochore Dynein is indeed a component of the fibrous corona [285], but direct demonstration of this hypothesis came only several years later. By studying the specific role of kinetochore Dynein by RNAi-mediated depletion of its kinetochore-targeting factor ZW10, as well as injection of function-blocking antibodies against Dynein Intermediate Chain, or injection of Dynamitin protein that disrupts the Dynein/Dynactin complex, several laboratories reported a role for Dynein in the fast poleward movement of chromosomes during the initial encounters between microtubules and kinetochores, but not in k-fiber formation [231,232,286]. Consequently, in some of these perturbations, particularly evident after ZW10 RNAi, some chromosomes failed to complete congression and remained outside the spindle pole with mono-oriented or unattached kinetochores [231,232]. Similar findings were also reported after RNAi of Spindly, a protein that is required to recruit Dynein to kinetochores without affecting the SAC [179]. Overall, these data indicated a role for kinetochore Dynein in the poleward movement of chromosomes during early prometaphase, with possible implications for the mechanism of congression in a subset of chromosomes.

In addition to a microtubule minus-end-directed motor activity, in vitro studies have also revealed the existence of a microtubule plus-end-directed activity at kinetochores from purified chromosomes [287,288]. Independent work by Yen and colleagues led to the discovery of CENP-E, which is enriched at prometaphase kinetochores [289] and was subsequently shown to be a Kinesin-like (Kinesin-7) motor protein [290] associated with the kinetochore fibrous corona [291,292]. Direct demonstration of microtubule plus-end-directed activity was obtained after characterization of CENP-E in *Xenopus*, where immunodepletion/immunoblocking experiments in oocyte extracts revealed a role in chromosome alignment [293]. Similar findings were reported after microinjection of function-blocking antibodies, expression of a dominant-negative motor-less CENP-E construct and antisense oligonucleotide blocking in human cells in culture [294,295] or analysis of CENP-E mutants in *Drosophila* [296]. However, these experiments were unable to make a clear distinction whether CENP-E motor activity was required for chromosome congression or maintenance of chromosome alignment after reaching the equator. This was only firmly established by live-cell recordings from nuclear envelope breakdown (NEB) after perturbation of CENP-E function by antibody microinjection in human cells in culture, where some chromosomes that were found to undergo initial poleward movement were unable to complete congression within the next 2h after NEB [297]. Overall, these studies demonstrated the existence of a Kinesin-like motor protein with microtubule plus-end-directed activity that is associated with the kinetochore fibrous corona and plays a role in chromosome congression. Importantly, because most chromosomes are able to align at the equator after perturbation of CENP-E function, it was concluded that the dependence on CENP-E for chromosome congression must be critically linked to chromosome position within the spindle (see Section 2.6), further demonstrating the existence of concurrent mechanisms. 

**Figure 5:**
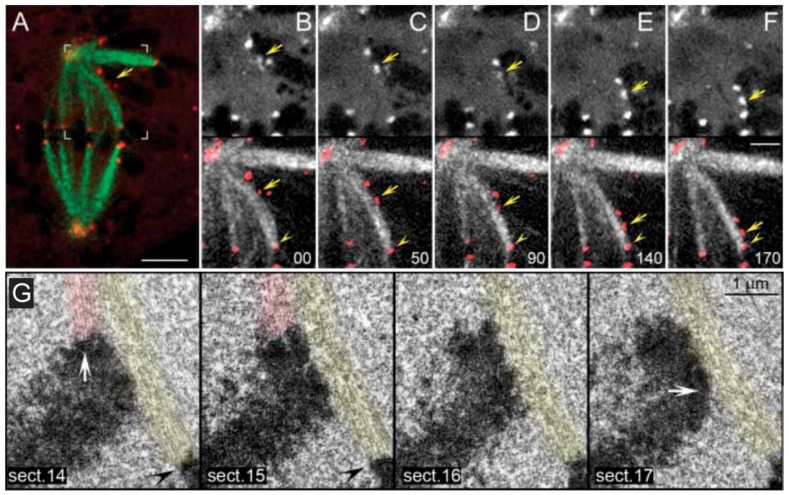
Demonstration that chromosome congression is independent of bi-orientation. From A-F, the movement of a polar chromosome along a pre-existing k-fiber is illustrated in a PtK1 cell. The leading kinetochore is indicated (yellow arrows). The kinetochore of a neighbor k-fiber on a bi-oriented chromosome is also indicated (yellow arrowheads). Time is in sec. In G, serial sections of a sliding mono-oriented chromosome with the leading kinetochore laterally attached to a neighbor k-fiber. Kinetochores of the congressing chromosome are indicated (white arrows), as well as the kinetochore of a neighbor k-fiber (black arrowheads). Images adapted from Kapoor et al., 2006 [300]. Reprinted with permission from The American Association for the Advancement of Science (AAAS).

For years, it was believed that CENP-E function at kinetochores required for chromosome congression and bi-orientation was related to the regulation of end-on kinetochore microtubule attachments [297,298,299], in part through a contribution of CENP-E in maintaining attachment of kinetochores to the end of a depolymerizing microtubule [273]. However, this capacity to couple kinetochores to depolymerizing microtubule plus-ends does not require ATP, suggesting that the role of CENP-E in chromosome congression relies on a different mechanism. The paradigm shift occurred after the demonstration that chromosomes can congress to the spindle equator before bi-orientation [300]. In this work, Khodjakov and colleagues demonstrated that mono-oriented chromosomes located near the poles could glide towards the equator along pre-existing spindle microtubules, including k-fibers, in a CENP-E-dependent manner. These observations provided an explanation for the involvement of CENP-E microtubule plus-end-directed motility at the kinetochore fibrous corona for chromosome congression (Figure 5). 

One controversial issue has been related with CENP-E processivity. In vitro microtubule gliding assays with recombinant CENP-E motor domain revealed a velocity around 5 μm/min [293,301]. Similar microtubule gliding assays with the full-length protein reported velocities around 1 μm/min [301,302]. More recently, single CENP-E molecule measurements (either the full length or motor domain only) have indicated a much faster velocity in the order of 20 μm/min [303,304], suggesting that CENP-E binding to the coverslip in traditional gliding assays is partially inhibitory of its function. Interestingly, the measured chromosome velocity during CENP-E-dependent congression of polar chromosomes in human cells was around 1.5 μm/min [6,305] indicating that, in vivo, cumulative CENP-E processivity is significantly attenuated by a yet unknown mechanism. One possibility could be related with the presence of non-motile microtubule-associated proteins (MAPs) or residual Dynein activity on microtubules that could slow down CENP-E-dependent transport of chromosomes during congression in vivo.

### 2.6. Chromosome Positioning Relative to Spindle Poles at NEB Defines the Mechanism of Congression 

Another critical question has been what determines that some chromosomes use (or not) the motor-dependent pathway for congression. Classical correlative light and electron microscopy studies in PtK1 cells at the onset of prometaphase have suggested that chromosomes that were equidistant from the two spindle poles immediately bi-orient (the so-called “direct congression”), whereas chromosomes that were closer to only one of the spindle poles become mono-oriented before congressing to the equator [282,306]. Interestingly, inhibition of CENP-E function in human cultured cells only prevents congression of about 20% of the chromosomes [6,241], suggesting that most chromosomes utilize a motor-independent pathway to align at the equator. By back-tracking those chromosomes that were found locked at the spindle poles after CENP-E inhibition, it was found that they were mostly located outside the interpolar region at NEB [6], suggesting that chromosomes that are favorably positioned between the two spindle poles at NEB undergo direct motor-independent congression involving PEFs and kinetochore-pulling forces after bi-orientation. This might be facilitated by the organization of chromosomes in a ring-like configuration and by the expansion of the outer kinetochore, thereby facilitating microtubule capture and immediate bi-orientation during early prometaphase [72,307]. Interestingly, early embryonic divisions in the nematode *C. elegans*, which lacks a CENP-E orthologue but has holocentric centromeres extending along the entire chromosome length, occur in a stereotypical manner, always with two fully separated centrosomes at NEB [308]. The combination of large kinetochores with fully separated centrosomes at NEB might favor the direct congression of chromosomes in this system, where PEFs mediated by Chromokinesins also appear to play a critical role [309]. Thus, the action of Dynein and CENP-E motors at kinetochores appears to be only critical to align peripheral chromosomes that lie much closer to one of the spindle poles, where bi-orientation at NEB is unlikely to occur. A corollary of this hypothesis is that the action of kinetochore Dynein in bringing peripheral chromosomes to the vicinity of the spindle poles after initial lateral attachments, followed by CENP-E-mediated congression, increases the chances of bi-orientation as chromosomes approach the equator.

### 2.7. Coordination between Kinetochore- and Arm-Associated Motors

As all great solutions to a problem, they usually open up more questions. The existence of two distinct motor activities operated by Dynein and CENP-E, both localized at the kinetochore fibrous corona, but with opposite directional preferences along microtubules, posed obvious questions regarding their coordination to mediate chromosome congression (see Section 2.8 and Section 2.9). In addition, the identification of microtubule plus-end-directed activities at kinetochores and chromosome arms demanded clarification of their relative contribution in moving chromosomes away from the pole. The critical role of kinetochores for chromosome movement towards the equator is known since the works of Zirkle and colleagues using focused UV or proton microbeams on parts of chromosomes in cultured newt cells [278,279,280]. They found that “centrophilic” chromosomes in which the kinetochore region was irradiated lost their ability to move in a directed fashion, drifted about until anaphase and never joined the metaphase plate. Similar findings were later reported in PtK1 and PtK2 cells [23,310,311]. These observations indicate that despite the action of PEFs on chromosome arms [43], they are not sufficient to drive the congression of “centrophilic” chromosomes. Moreover, these observations demonstrate that kinetochores are essential for this process, suggesting a dominant role over PEFs. Work by Brinkley and colleagues using CHO cells undergoing MUGs, in which kinetochores completely detach from chromatin, has further demonstrated that kinetochores are not only required, but they are also sufficient to ensure chromosome migration to the equator [312,313] (see also [314] for similar findings in HeLa cells undergoing MUGs). However, it should be noted that, under these circumstances, chromatin-detached kinetochores frequently establish unorthodox attachments with spindle microtubules, mostly resulting in merotelic attachments in which the same kinetochore binds microtubules from opposite poles [313,314]. In agreement, merotelic attachments on chromosome fragments with only one kinetochore have been shown to support chromosome congression [315]. 

A systematic dissection of the respective roles of kinetochore- and arm-associated motors for chromosome congression in human cells has been recently performed [6]. Accordingly, by combining molecular perturbations of the different motor functions with laser microsurgery of chromosome arms, it was shown that “centrophilic” chromosomes rely on CENP-E motor activity at kinetochores to counteract Dynein-mediated poleward force and move towards the equator. When chromosome arms were released from the kinetochore region by laser microsurgery, about 20% of them did not move towards the equator. Instead, they moved away towards the cortex in a Chromokinesin-dependent manner. Thus, although Chromokinesin-mediated PEFs can mediate chromosome ejection away from the poles, CENP-E-mediated forces at kinetochores are dominant and required to bias chromosome motion exclusively towards the equator. This work further demonstrated that kinetochore Dynein activity is dominant over PEFs along chromosome arms and this is required for poleward motion after initial lateral kinetochore-microtubule attachments. This role of Dynein prevents random chromosome ejection and stabilization of end-on kinetochore-microtubule attachments on chromosomes positioned near the poles due to the action of PEFs along chromosome arms, while bringing chromosomes close to the highest Aurora A activity near the poles [6,7,77,316]. This explains why “centrophilic” chromosomes after perturbation of CENP-E function move abnormally close to the pole and are mostly devoid of end-on attached microtubules [297,298] and lack any detectable oscillatory motion [295,297]. Overall, Dynein activity was proposed to prevent the formation of premature/erroneous kinetochore-microtubule attachments, thereby allowing CENP-E to undergo processive motion necessary to transport polar chromosomes along pre-existing spindle microtubules towards the equator [6,316].

Interestingly, CENP-E activity at kinetochores was shown to be required for chromosome ejection from the poles, including in monopolar spindles in which chromosome bi-orientation does not take place [6], probably by mediating the motion of leading kinetochores [300], since trailing kinetochores do not seem to exert a significant pushing force [48]. Intriguingly, CENP-E activity required for chromosome congression is independent of the establishment of stable end-on kinetochore-microtubule attachments and the formation of k-fibers, but appears to require spindle microtubule stabilization [305,317]. In contrast, Dynein was found to counteract PEFs also in monopolar spindles [6,179,231]. Thus, both CENP-E and Dynein are dominant over PEFs and play antagonistic roles at the kinetochore, independently of the establishment of stable end-on kinetochore-microtubule attachments and chromosome bi-orientation. Finally, simultaneous inhibition of all kinetochore and arm-associated motors did not prevent congression of all chromosomes [6], further demonstrating the existence of motor-dependent and -independent pathways that ultimately mediate the alignment of all chromosomes at the spindle equator. 

### 2.8. Motor Regulators

The mechanism of chromosome congression independent of chromosome bi-orientation requires the spatial and temporal coordination of different motor activities. For instance, the direction of motor movement at kinetochores in vitro has long been known to be regulated by phosphorylation, namely by the activation of the plus-end-directed and/or inactivation of the minus-end-directed motor activities at kinetochores [288]. The kinetochore motor CENP-E is extensively phosphorylated during mitosis [318], although the functional significance of many of these phosphorylation events is not completely understood. CENP-E phosphorylation at its C-terminal tail by Cdk1 and MAPK regulates CENP-E interaction with microtubules [319,320]. This C-terminal tail is able to completely block CENP-E motility in vitro due to a direct interaction with the motor domain [301]. This auto-inhibition of CENP-E can be reversed by Mps1- or Cdk1-mediated phosphorylation of its C-terminal tail, thereby restoring normal CENP-E motility in vitro [301]. Additionally, CENP-E is phosphorylated in a conserved residue (T422) close to the motor domain by Aurora A and B [321]. This phosphorylation reduces the affinity of CENP-E for microtubules and is required for congression of polar chromosomes. However, it remains unclear how a reduction in microtubule affinity would promote CENP-E processivity necessary to overcome Dynein-mediated poleward motion. Importantly, dephosphorylation of CENP-E at T422 by PP1 phosphatase is required for stable chromosome bi-orientation after congression [321]. The recent demonstration of the existence of an Aurora A activity gradient from the spindle poles [7] has provided the necessary positional cues to control the extent of CENP-E phosphorylation at T422 as polar chromosomes approach the equator. Interestingly, Dynein intermediate chain is phosphorylated by Plk1 on T89 also in a chromosome position-dependent manner and this appears to be counteracted by PP1 phosphatase [322,323]. This phosphorylation is required for normal Dynein recruitment to kinetochores and inhibits its association with Dynactin, as well as Dynein poleward streaming along attached microtubules. Since Dynactin is required for cytoplasmic Dynein processivity [324,325], these results suggest that Dynein phosphorylation at T89 is inhibitory of its motor-mediated transport functions, as originally predicted by in vitro studies [288].

The role of CENP-E in polar chromosome congression is also regulated by sumoylation and farnesylation. When sumoylation is inhibited by overexpressing the SUMO isopeptidase SENP2, CENP-E no longer localizes to kinetochores and chromosome congression is impaired [326]. Interestingly, cells treated with farnesyltransferase inhibitors (FTIs) exhibit a prometaphase delay, suggesting the involvement of farnesylated proteins in chromosome alignment [327,328,329,330] (see also Section 4.2). These mitotic defects observed after treatment with FTIs were initially attributed to the inhibition of CENP-E and CENP-F farnesylation [327,328,331]. While inhibition of farnesylation appears to interfere with CENP-E association with microtubules [327], the role of farnesylation in regulating CENP-E localization and function at kinetochores remains controversial. Treatment of cells with FTIs was reported to deplete CENP-E and CENP-F from metaphase, but not from prometaphase kinetochores [328]. CENP-E is also degraded shortly after mitotic exit [332], and its degradation requires farnesylation [333]. Interestingly, it was suggested that farnesylation of Spindly is also involved in the regulation of kinetochore Dynein, since mutation of a potential farnesylation site in Spindly prevented its localization at the kinetochore [179]. More recently, two independent studies confirmed Spindly as a farnesylation substrate [334,335]. In one study, FTI treatment resulted in loss of Spindly at kinetochores without affecting the RZZ complex or CENP-E and CENP-F kinetochore localization [335]. In contrast, in another study, CENP-E and CENP-F kinetochore levels were also affected by FTI treatment, but to a less extent compared to Spindly [334]. Both studies have shown that preventing farnesylation of Spindly delays chromosome congression, producing a similar phenotype observed in cells treated with FTIs. Taking these findings together, it seems that the role of farnesylation in regulating CENP-E function during chromosome congression rather represents a minor effect, while loss of Spindly kinetochore localization (and consequently Dynein) after farnesylation inhibition appears to be the major contributing factor to the congression defects observed in cells treated with FTIs. 

Different studies have implicated Mps1 in chromosome alignment, but the underlying molecular mechanism remains unclear [336,337,338,339]. Initially it was proposed that regulation of chromosome alignment by Mps1 acts through modulation of Aurora B kinase activity [337]. However, recent studies have provided evidence that regulation of chromosome alignment by Mps1 is independent of Aurora B [336,340,341]. The regulation of chromosome alignment by Mps1 may be through CENP-E phosphorylation [301], as this is necessary to recruit CENP-E to kinetochores [336,342]. These results suggest that the role of CENP-E in polar chromosome congression might be regulated by Mps1. 

Finally, motor proteins involved in chromosome congression are also regulated by proteolysis. For instance, the Kinesin-10 Kid and the kinetochore motor CENP-E are degraded at the end of mitosis, consistent with down-regulation of PEFs at the metaphase-anaphase transition to allow chromosome poleward movement [44,65,332]. 

### 2.9. The Role of Tubulin PTMs as a Navigation System for Kinetochore-Based Motility of Chromosomes

In addition to the regulation of kinetochore motor activities, the possibility that tubulin post-translational modifications (PTMs), as part of the so-called “tubulin code” [343,344], additionally contribute with spatial cues required for chromosome congression has recently been proposed [345,346]. Tubulin, the building unit of microtubules, can be enzymatically processed to undergo different PTMs, including detyrosination, (poly)glutamylation, glycylation, phosphorylation, acetylation and the recently-discovered methylation [344,347]. Some of these modifications have been already shown to regulate the motor activity of Kinesin-1, affecting its binding and transport in neurons [348,349,350,351]. In vitro reconstitution assays have further dissected the impact of tubulin PTMs on the performance of motor proteins such as Kinesin-1, Kinesin-2, Kinesin-13, and Dynein [352,353]. Therefore, it is plausible that the activities of the motor proteins involved in the directed transport of chromosomes along distinct microtubule populations, before and during chromosome congression, are also regulated by PTMs that differentiate the microtubule tracks on which they move [346]. Indeed, it has been known for decades that different PTMs label distinct microtubule populations within the mitotic spindle [354,355,356,357]. For instance, the dynamic, short-lived astral microtubules that extend from the spindle poles towards the cell cortex are highly tyrosinated (i.e. they contain a tyrosine as the last amino acid on the α-tubulin C-terminal tail), while more stable spindle microtubules, such as k-fibers and possibly interpolar microtubules, are detyrosinated, acetylated and polyglutamylated [354,355,356,357]. Therefore, this patterned distribution of different tubulin PTMs within the mitotic spindle could work as a navigation system for kinetochore-based motor proteins involved in the critical steps that anticipate and mediate chromosome congression [345,346]. 

Such a navigation system would have particular implications for the congression of peripheral chromosomes that are unable to bi-orient soon after NEB. According to this model, the Dynein-mediated poleward movement of peripheral chromosomes upon the initial interaction with astral microtubules would be regulated by their high tyrosinated state [355,356]. In support of this concept, recent in vitro reconstitution studies of Dynein/Dynactin activity have indicated that tubulin C-terminal tail tyrosination is of great importance for Dynactin-mediated initiation of Dynein motion on microtubules [358]. Similar findings have been reported in vivo, where the Dynactin subunit p150 and tubulin tyrosination were shown to mediate the initiation of retrograde vesicle transport in neurons [359]. Finally, these data are in line with previous studies reporting that p150/Dynactin has higher affinity for tyrosinated microtubules [325,360] and that the motility of both cytoplasmic and axonemal Dyneins highly depends on tubulin C-terminal tails [325,361,362,363].

After the initial Dynein dominance during the poleward transport of peripheral chromosomes along tyrosinated astral microtubules, Dynein is overtaken by CENP-E to drive the congression of polar chromosomes to the equator [6]. In concert with Aurora A kinase-mediated activation of CENP-E by phosphorylation near the poles [321], and in agreement with the slow association of CENP-E with microtubules observed in vitro [364], recent work revealed that CENP-E has a preference for the more stable detyrosinated spindle microtubules, and this is important to guide polar chromosomes towards the equator [345]. Accordingly, this study showed that, similar to CENP-E depletion/inhibition, attenuation of tubulin detyrosination either by inhibition of the tubulin carboxypeptidase (TCP) (the enzyme that removes the last tyrosine from the α-tubulin C-terminal tail on polymerized microtubules), or by overexpression of the tubulin tyrosine ligase (TTL) (the enzyme that adds back tyrosine to soluble α-tubulin), prevented polar chromosomes from congressing. In vitro reconstitution experiments confirmed that CENP-E motility is enhanced on detyrosinated microtubules [345]. Moreover, RNAi-mediated depletion of TTL, which increases overall detyrosination of the mitotic spindle, including astral microtubules, prevented peripheral chromosomes from reaching the spindle pole [345]. Since this could only be partially rescued by co-depletion of CENP-E [345], it suggests that increased detyrosination of astral microtubules further prevents kinetochore Dynein-mediated poleward transport. Altogether, these data support that the state of α-tubulin detyrosination provides important spatial cues for the regulation of chromosome movements during mitosis [346]. As so, the difference in detyrosination levels between highly dynamic astral and more stable spindle microtubules mediates an activity switch that enables the fine spatiotemporal regulation of the opposite motility of Dynein and CENP-E at kinetochores. This ensures that peripheral chromosomes are first transported poleward by Dynein along tyrosinated astral microtubules, followed by CENP-E-mediated congression along more detyrosinated microtubules pointing to the equator. 

This activity switch seems to be very finely regulated, since in vitro studies showed that tubulin (de)tyrosination induced less than 2- and up to 4-fold changes in the processivity of CENP-E and Dynein motors, respectively [345,358]. Importantly, a recent in vitro reconstitution study demonstrated that single Kinesin and Dynein motors produce approximately similar forces [365], which helps to explain how slight differences in tubulin (de)tyrosination can influence motor kinetics and determine the directionality of chromosome movements. This is further supported by recent theoretical work, which demonstrated that tubulin PTMs are sufficient to generate a 2-fold difference on motor kinetics and target cargoes to specific locations along microtubules [366].

A critical emerging question is how a single amino acid change at the α-tubulin C-terminal tail selectively affects motor recognition and function at the structural level. It is well established that tubulin C-terminal tails regulate the binding and processivity of Kinesin-1 and Dynein in vitro [362,367]. CryoEM, backed-up by crystallographic studies, have allowed the visualization of the CENP-E motor domain in complex with microtubules [368,369]. Although the exact interaction between CENP-E and tubulin C-terminal tails has not been determined due to their flexible nature, these works indicate that the CENP-E motor domain might interact with helix 12 from α-tubulin, close to the C-terminal tail. Because the association of the CENP-E C-terminal kinetochore-binding domain with microtubules depends little (20% reduction) on tubulin C-terminal tails [370], these results suggest that microtubule detyrosination directly regulates recognition by the CENP-E motor domain. In contrast, the recognition of tyrosinated microtubules by Dynein has been shown to involve p150/Dynactin [358,360] and structural reconstructions have indicated that this interaction is mediated by the GKNDG motif on the CAP-Gly domain of p150/Dynactin [371,372].

### 2.10. Chromosome Congression vs. Maintenance of Alignment

**Figure 6:**
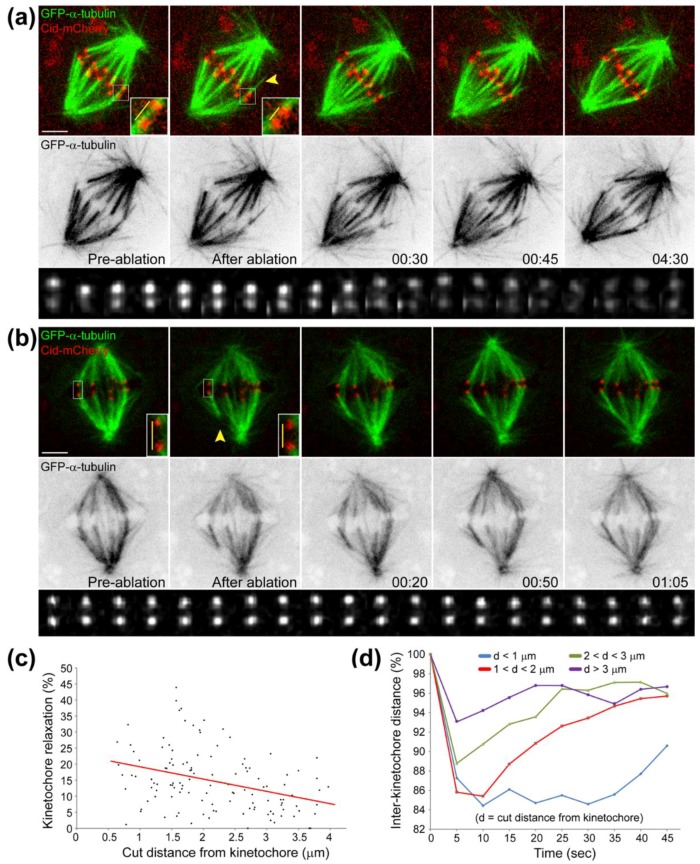
Forces at kinetochores are proportional with k-fiber length, but chromosome position at the equator is independent of k-fiber length. (**a**,**b**) Laser microsurgery of k-fibers in *Drosophila* S2 cells stably expressing GFP-α-tubulin to label microtubules (green) and Cid-mCherry to label kinetochores (red). K-fibers were cut (yellow arrowhead) and grew back as described previously [29]. Inverted contrast of GFP-α-tubulin is also shown, as well as the variation of inter-kinetochore distance over time (kymograph; first frame corresponds to pre-surgery distance; second frame onwards are after surgery). Measurement of the inter-kinetochore distance before and after laser surgery ablation of k-fibers (yellow bars) indicates that kinetochores relax after surgery, and this relaxation is more evident the closer the cut is to the kinetochore. Time is in min:sec. White scale bars are 2 μm; (**c**) Quantification of the percentage of kinetochore relaxation after surgery (determined by the difference between initial inter-kinetochore distance and the minimum observed distance after surgery) indicates a negative correlation (R^2^ = −0.361; *p* < 0.001) with the cut distance from the kinetochore (n = 125 cells); (**d**) Corresponding quantification of the inter-kinetochore distance over time as a function of the cut distance from the kinetochore. Each group was normalized against its initial distance such that one hundred percent corresponds to the average initial distance. The closer the cut is to the kinetochore, the longer the recovery of inter-kinetochore distance and the higher is the relaxation. The inclusion of a kinetochore marker in this study and the observed variability of inter-kinetochore distance after k-fiber cut explains previous observations in which no detectable kinetochore relaxation was observed without the use of a kinetochore marker [29]. Laser microsurgery was performed essentially as described in [373].

One poorly understood aspect of mitosis is whether the mechanisms that mediate chromosome congression consist of the same principles that ensure the maintenance of a bi-oriented chromosome at the equator after completing congression. Clearly, motor-dependent chromosome congression does not rely on a force balance on a given kinetochore pair, as chromosome bi-orientation is not required to complete congression [300]. Moreover, end-on kinetochore-microtubule attachments are not even required for motor-driven congression to the equator, but are essential to maintain aligned chromosomes at the metaphase plate [305]. This is corroborated by microsurgery experiments in which the kinetochore region of a once aligned chromosome is irradiated with a focused UV or laser microbeam, causing the chromosome to immediately move towards the direction of the undisturbed kinetochore [20,21,22,23]. In contrast, when k-fibers are cut on a bi-oriented chromosome positioned at the equator, chromosomes either do not shift at all or shift only slightly towards the pole of the unperturbed k-fiber [21,22,29,30,31,32,33,34,35,36,37,38]. Interestingly, inter-kinetochore tension in vertebrate and insect cells is proportional to k-fiber length [37,38] (Figure 6). Overall, these data indicate that while force at kinetochores is proportional to k-fiber length, maintenance of chromosome position near the equator is not.

Several theoretical and experimental studies have predicted or provided evidence for mechanical coupling between kinetochore and non-kinetochore (interpolar) microtubules [4,37,38,374,375,376,377,378,379,380,381], which might account for the maintenance of chromosome positioning at the equator independently of k-fiber length. While the molecular nature of this spindle microtubule coupling system remains unknown, it is likely to involve multiple players that possess the necessary molecular properties to serve this purpose. These include several MAPs and motors with microtubule cross-linking properties, such as PRC1, Kinesin-5, Kinesin-15, CLASPs, Clathrin/Ch-TOG/TACC3, Asp, NuMa, Kinesin-14 and Dynein [382,383,384]. In addition, Chromokinesins, Kif4A in particular, might also work as a coupling element between k-fibers and interpolar microtubules interacting with chromosome arms [71].

Interestingly, many loss-of-function studies of Chromokinesins revealed only a very minor role during chromosome congression, while being critical to maintain chromosomes aligned at the equator [6,71]. These results suggest that Chromokinesins might additionally contribute to the stabilization of kinetochore-microtubule attachments of aligned chromosomes, possibly in coordination with the activity of Kinesin-8 [146]. Indeed, recent works in *Drosophila* S2 cells have shown that Chromokinesins promote kinetochore-microtubule stabilization and the conversion from lateral to end-on attachments, independently of chromosome bi-orientation [77,81], which might be important to maintain chromosomes aligned at the equator after congression. This implies that CENP-E is no longer dominant over Chromokinesins once chromosome bi-orientation and equatorial alignment is achieved. This would be consistent with the finding that CENP-E levels at the kinetochore decrease significantly due to Dynein-mediated stripping upon microtubule attachment and chromosome bi-orientation [385]. However, whether CENP-E plays a role in maintaining chromosome positioning at the equator after alignment has been controversial. For instance, CENP-E has been proposed to play a role in stabilizing end-on kinetochore-microtubule attachments [297,298,299]. This model is supported by electron microscopy studies after inactivation of CENP-E function, which showed a reduced microtubule number at kinetochores of aligned bi-oriented chromosomes, supporting a role for CENP-E after chromosome congression [297,298]. Importantly, the observed differences relative to controls appear to be attenuated during a prolonged mitosis where the range of microtubule binding was similar to controls, indicating that CENP-E is not essential for binding of a full complement of microtubules at kinetochores of bi-oriented chromosomes [297]. Interestingly, original antibody micro-injection experiments in metaphase cells have indicated that CENP-E is not required for maintenance of chromosome alignment [289]. In contrast, treatment of metaphase cells with a CENP-E inhibitor that forces CENP-E to bind tightly to microtubules (a “*rigor*” state) caused the displacement of chromosomes from the equator, supporting a role of CENP-E in maintaining chromosome alignment after bi-orientation, in addition to mediating chromosome congression [303]. The availability of a second generation of CENP-E inhibitors that compromise ATPase activity without interfering with microtubule binding [386] will be important to clarify the role of CENP-E after chromosome alignment.

Finally, many studies have reported chromosome misalignment problems after functional perturbation of several proteins (see Table 1). However, since live-cell imaging was not used in many of these studies, it remains unclear whether it truly reflects a direct role of these proteins in chromosome congression or in the maintenance of chromosome alignment. The recent discovery that apparently unrelated experimental perturbations associated with a metaphase delay often lead to “cohesion fatigue” (i.e., the uncoordinated loss of sister chromatid cohesion after chromosome congression but prior to anaphase onset, due to the action of mitotic spindle forces) [155,387,388] incites for a systematic re-evalution of proteins formerly associated with chromosome alignment using state-of-the-art live-cell imaging techniques.

### 2.11. An Integrated Model of Chromosome Congression

**Figure 7:**
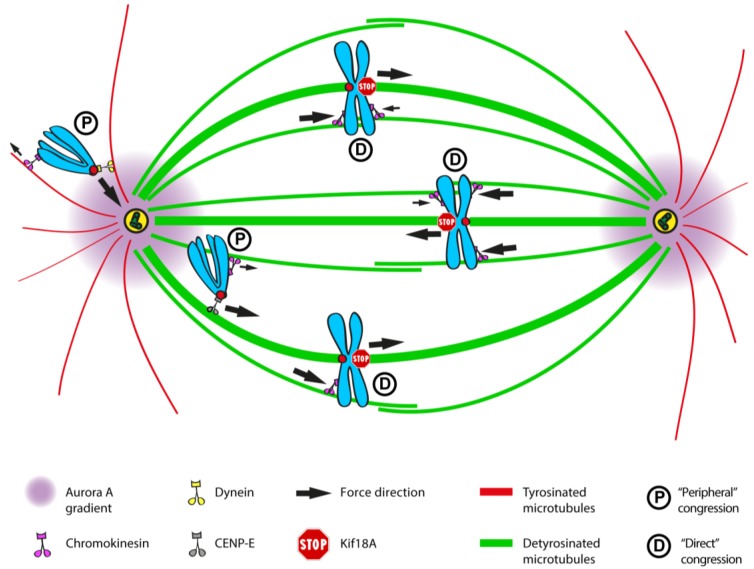
Integrated model of chromosome congression in human cells. In this representation, Kif18A is shown to restrict k-fiber length, thereby contributing to a directional switch and regulating chromosome oscillations after bi-orientation. See text for a detailed description.

Based on the arguments expressed in the previous sections, we propose that chromosome congression in humans can essentially be explained by two main mechanisms that operate in parallel (Figure 7), meaning that not all chromosomes rely on the same mechanism to complete congression. A key aspect that determines which mechanism is used depends essentially on whether chromosomes establish lateral or end-on attachments at their kinetochores on their way towards the equator. This is influenced by the position of chromosomes relative to the spindle poles at NEB. Those chromosomes that are able to bi-orient soon after NEB would use a “direct congression” mechanism in which opposite kinetochore-pulling forces, resulting from the tight regulation of microtubule dynamics and length at the kinetochores, in coordination with PEFs along chromosome arms, drive chromosome oscillations until net force is zero near the equator. A corollary from this model is that the establishment of stable end-on attachments inhibits the other congression mechanism relying on lateral interactions between microtubules and kinetochores. This second mechanism would take advantage of the high processivity of the Dynein/Dynactin motor localized on unattached kinetochores to capture peripheral chromosomes, which are unable to bi-orient at NEB and establish stable end-on kinetochore microtubule attachments. The minus-end directed motion of Dynein/Dynactin along tyrosinated astral microtubules transports peripheral chromosomes close to one of the spindle poles, where Aurora A activity is highest and prevents the stabilization of end-on kinetochore-microtubule attachments. This configuration also imposes a dominance of kinetochore Dynein/Dynactin over the action of Chromokinesin-mediated PEFs along chromosome arms that would otherwise promote the premature stabilization of end-on kinetochore-microtubule attachments and lead to errors resulting in chromosome missegregation. In addition, while travelling along tyrosinated astral microtubules, Dynein/Dynactin will be dominant over the other kinetochore motor, CENP-E, with plus-end-directed motility and a preference for more stable detyrosinated microtubules. Once at the poles, phosphorylation by Aurora A will activate CENP-E, (while other centrosome kinases, such as Plk1, inactivate Dynein/Dynactin), favoring the lateral transport of chromosomes by CENP-E along detyrosinated microtubules (either k-fibers or interpolar microtubule bundles) towards the equator, where the chances for bi-orientation are maximal. At the equator, Chromokinesins promote the conversion from lateral to end-on attachments, which further downregulates CENP-E and Dynein, thereby ensuring the maintenance of chromosome position at the metaphase plate. Once aligned and bi-oriented at the metaphase plate, the coordination between kinetochore-pulling forces and PEFs continue to determine the amplitude of chromosome oscillations, but maintenance of chromosome position near the equator will depend on additional factors that mediate the cross-linking between kinetochore and non-kinetochore microtubules.

### 2.12. A Note about Chromosome Congression in Acentrosomal Systems

The problem of chromosome congression in acentrosomal systems such as animal oocytes and land plants is not less complex than in mammalian somatic cells. While the lack of centrosomes could in principle simplify the process and decrease microtubule heterogeneity within the context of the spindle, these systems have developed alternative microtubule organizing structures or mechanisms that, in a way, functionally resemble the centrosomes. For instance, land plants assemble a “prophase spindle” on opposite sides of the nucleus before NEB. These prophase spindle microtubules undergo “search-and-capture” and eventually interact with chromosomes and assist their motion (reviewed in [39,389,390]). There is, however, good evidence that canonical PEFs are rather weak or absent in plants [39,391]. Mammalian oocytes form acentriolar microtubule-organizing centers (aMTOCs) that assemble transient “multipolar” spindles that ultimately cluster into a bipolar structure (and show astral-like microtubules) and mediate interactions with chromosomes towards bi-orientation [392,393]. Therefore, “direct congression” of at least some chromosomes after NEB is likely to take place in mammalian oocytes and land plants. In contrast, in *Xenopus* oocyte extracts, microtubules organize “inside-out” in the vicinity of chromatin and in a Ran-GTP-dependent manner (reviewed in [394]). As so, chromosomes already start “congressed” during spindle assembly and do not need to be transported from the poles. Nevertheless, CENP-E and Kid/Chromokinesin motors appear to be necessary to maintain chromosomes equidistant from the poles in this system, either by promoting chromosome bi-orientation or simply by mediating persistent microtubule plus-end-directed chromosome motion, such as in PEFs [65,66,293].

Recent insight from live-cell imaging of mammalian oocytes has revealed unprecedented details about the process of chromosome congression in this system [395]. It was found that chromosome congression is completed before bi-orientation due to the establishment of an intermediate configuration, the “prometaphase belt”, in which chromosomes are organized around the spindle. During congression, chromosomes that were located far from the equator moved towards it by sliding along spindle microtubules, whereas chromosomes that were already located near the equator remained stationary. Subsequently, chromosomes invaded the spindle area establishing the final metaphase plate organization and bi-orientation. Interestingly, very similar findings have been reported for human somatic cells in culture [72], suggesting conservation of the mechanisms of chromosome congression between mammalian centrosomal and acentrosomal systems. In support of this idea, chromosome congression in mammalian oocytes also does not seem to depend on the Chromokinesin Kid [395,396], but CENP-E activity appears to be required, possibly by facilitating bi-orientation [397]. Similar findings were also recently reported in *Drosophila* and *C. elegans* oocytes, in which prometaphase chromosome motion and bi-orientation was shown to depend essentially on lateral attachments [398,399]. However, while in *Drosophila* oocytes chromosome bi-orientation and lateral attachments were shown to rely on CENP-E [398], in the case of *C. elegans* oocytes the process might involve the Chromokinesin KLP-19 [399]. It should be noted that chromosomes in *Drosophila* oocytes are compacted into a karyosome and, similar to *Xenopus* oocyte extracts, congression is unnecessary, whereas in *C. elegans* KLP-19 is only required for chromosome alignment in metaphase I-arrested, but not normally progressing oocytes [399,400]. Therefore, CENP-E and Chromokinesin activities in these systems might only be required to maintain chromosomes at the equator. In the case of land plants, they appear to lack cytoplasmic Dynein motors [390,401], but CENP-E-like Kinesin-7 motors and Chromokinesins are conserved [390,402] and the former has been implicated in chromosome congression in moss, even though it does not seem to localize at kinetochores [403]. Finally, it is worth remarking that even in animal somatic cells in which centrosome function was genetically perturbed, chromosome congression was delayed but not prevented, further supporting a marginal role for centrosome-mediated PEFs in chromosome alignment in metazoans [404]. 

## 3. Consequences of Abnormal Congression

### 3.1. Aneuploidy, Tumor Suppression and Oncogenic Potential

Aneuploidy is defined as a karyotype state with a chromosome number that deviates from a multiple of the haploid, and is a hallmark of human cancers. Aneuploidy is often accompanied by high rates of chromosome missegregation, a phenomenon called chromosomal instability (CIN), in which chromosomes are permanently gained and lost during multiple divisions [405]. Therefore, CIN might contribute to tumorigenesis by changing the dosage of oncogenes and tumor suppressors required for tissue homeostasis. CIN has also been associated with both poor patient prognosis and resistance to some chemotherapeutic agents [406,407,408,409,410]. Paradoxically, there is also evidence that excessive CIN is a disadvantage for tumor progression and is associated with better prognosis [411]. Whatever the case may be, and despite all controversy, direct targeting of CIN as a potential anti-cancer therapy is now the subject of active research [412,413]. 

Chromosome congression defects are amongst the multiple pathways that could lead to CIN [405,414,415]. Different studies reported that cell and animal models with reduced levels of CENP-E generate high levels of aneuploidy. CENP-E deletion in mouse embryonic fibroblasts (MEFs) and in liver tissues resulted in cells with several mitotic defects, including chromosome misalignment and increased levels of lagging chromosomes, an indication of chromosome missegregation [298,416]. Homozygous disruption of the CENP-E gene causes early embryonic lethality [298], while heterozygous loss of CENP-E causes aneuploidy and CIN that can both promote or suppress tumor formation, depending on the context [417,418]. Mice heterozygous for CENP-E show a mild increase in the rate of spontaneous lung and spleen tumors, but exhibit a decreased incidence of liver tumors [418]. CENP-E heterozygosity did not accelerate tumor initiation or progression after treatment with the chemical carcinogen DMBA [417,418]. Moreover, when CENP-E heterozygosity was combined with the loss of the tumor suppressor p19ARF (CENP-E^+/−^ p19ARF^−/−^), most of the animals showed a strong delay in tumorigenesis [417,418]. Furthermore, exacerbating the level of CIN in CENP-E^+/−^ mice by crossing them with Mad2^+/−^ or APC^Min/+^ resulted in increased cell death and reduced tumor progression [417,419]. These findings suggest that low levels of CIN caused by minor chromosome congression and segregation defects could potentially lead to transformation, whereas an elevated rate of CIN inhibits tumor formation.

*Drosophila* models have also been generated to investigate whether induction of aneuploidy by knocking down CENP-E is tumorigenic . In one study, CENP-E depletion alone was not sufficient to drive tumorigenesis [420]. However, another study found that knockdown of CENP-E and Nsl1 (which targets Bub3 to the kinetochore, compromising the SAC) induced a tumorigenic response [421]. These results suggest that, per se, minor chromosome congression defects are insufficient to drive tumor formation in flies and that a significant level of aneuploidy is required.

Altered expression or mutations in CENP-E have been reported in some human diseases. CENP-E is upregulated in individuals with rheumatoid arthritis [422] and with breast cancer [423]. Moreover, CENP-E expression negatively correlated with disease-specific survival in patients with breast cancer [423]. In contrast, human hepatocellular carcinoma exhibits abnormally low levels of CENP-E [424]. Several non-synonymous single nucleotide polymorphisms were also reported in CENP-E and the Y63H point mutation, which disrupts the native conformation of the ATP-binding region in the CENP-E motor domain, was found to be associated with cancer [425]. Finally, mutations in CENP-E leading to chromosome congression problems were also associated with microcephalic primordial dwarfism (MPD) [426].

Kif18A is overexpressed in human colorectal [427] and human breast cancers [428]. Kif18A expression in breast cancers correlates with tumor grade, metastasis and survival, whilst suppression of Kif18A expression in breast cancer cells inhibits tumor growth in vivo [428]. In addition, proteomic analysis identified Kif18A as a potential biomarker of cholangiocarcinoma and lung cancer [429,430]. Genetic studies in mice demonstrated that disrupting Kif18A function affects male, but not female, fertility [193]. Kif18A^−/−^ male mice develop relatively normally and exhibit defects in the testis, but not in other organs. Testis atrophy in these mice is caused by impaired microtubule dynamics and loss of spindle pole integrity associated with chromosome congression defects during mitosis and meiosis. Another study showed that depletion of Kif18A protects animals from colitis-associated colorectal (CAC) cancers [431]. Although suggestive, the involvement of Kif18A in cancer requires further investigation.

Besides its function during chromosome congression, the Chromokinesin Kif4A plays several other roles throughout mitosis, and loss of this protein leads to various mitotic defects including chromosome hypercondensation, aberrant spindle formation, anaphase bridges, defective cytokinesis and aneuploidy [69,432]. Kif4A is absent or expressed at low levels in 35% of human cancers [433]. Kif4A is also downregulated in gastric carcinoma tissues and Kif4A expression levels correlate with tumor differentiation [434]. Interestingly, overexpression of Kif4A in gastric cancer cells inhibits proliferation in vitro, as well as the ability to form tumors in vivo [434]. Kif4A is also overexpressed in cervical cancer [435] and non-small cell lung cancer associated with poor patient outcome [436]. Furthermore, loss of Kif4A in murine embryonic stem cell results in several mitotic defects, including chromosome misalignment, spindle defects and aberrant cytokinesis [433]. Additionally, a high percentage of cells lacking Kif4A are aneuploid and injection of these cells into nude mice has the ability to form tumors. Based on these findings, the aneuploidy associated with aberrant mitosis after Kif4A depletion can promote tumor formation, but it remains unclear whether this is a direct consequence of its role in chromosome congression. Altogether, these findings demonstrate that loss of different Kinesin-like proteins involved in different aspects of chromosome congression might lead to aneuploidy. 

## 4. Targeting Chromosome Congression for Cancer Therapy

### 4.1. CENP-E Inhibitors

Microtubule poisons that disrupt spindle assembly and function have demonstrated to be powerful tools in the treatment of many human cancers [437], but their efficacy is limited by side effects such as neurotoxicity, neutropenia and acquisition of resistance [438,439,440]. Taxanes and vinca alkaloids are amongst the most successful microtubule drugs and are known to compromise chromosome congression by preventing the formation of proper kinetochore-microtubule attachments that nevertheless satisfy the SAC, leading to an abnormal mitotic exit and apoptosis [441,442,443,444,445]. The discovery of new mitotic targets for cancer therapy has raised interest in developing antimitotic agents that do not target microtubules [446,447]. The most notable targets are the Aurora kinases A and B, as well as Plk1 [448]. Although there are obvious drawbacks (and the main reason for failure in clinical trials) related with cytotoxicity of normal fast dividing cells, such as those in the bone marrow, gut, and hair follicles, protein targets that are only expressed in dividing cells are attractive for cancer therapy, since non-dividing differentiated cells should not be affected. CENP-E is expressed predominantly in mitosis (and G2) [290] and plays an important role in peripheral chromosome congression [293,295,300], thereby representing an attractive target for cancer therapeutics. GSK923295 is an allosteric inhibitor of CENP-E that blocks its microtubule stimulated ATPase activity and stabilizes the interaction between the motor domain and microtubules [449,450]. GSK923295 has demonstrated both in vitro and in vivo antitumor activity against various malignancies [449,451,452,453,454,455]. Cells treated with GSK923925 assemble bipolar spindles and the majority of chromosomes align at the spindle equator. However, some chromosomes remain clustered near the spindle poles, leading to mitotic arrest and apoptosis [449,456]. The antitumor activity of GSK923925 has been evaluated in combination with standard chemotherapies, as well as with other emerging targeted drugs [454]. Inhibition of ERK1 revealed a significant synergistic proliferation inhibition activity when combined with GSK923225 in neuroblastoma, lung, pancreatic and colon carcinoma cell lines [454]. Combination of GSK923225 with Pgp-pump modulators also appeared to improve the antitumor effects against cells with Pgp overexpression, thereby overcoming the resistance to Pgp inhibitors [457]. 

Another CENP-E inhibitor, PF-2771, selectively inhibits proliferation of basal breast cancer cell lines compared with normal and premalignant cells. Moreover, the sensitivity to this inhibitor correlates with the degree of CIN, suggesting that cancers with elevated CIN may benefit from CENP-E-targeted therapy [423]. Finally, inhibition of CENP-E motor function by PF-2771 resulted in tumor regression in a patient-derived basal-like breast cancer xenograft tumor model [423]. More recently, a new inhibitor of CENP-E directly targeting its ATPase activity, known as compound A, was found to have anti-proliferative activity in multiple cancer cell lines and in a xenograft nude mouse model [386,458]. CENP-E inhibition using compound A resulted in p53-dependent post-mitotic apoptosis triggered by elevated chromosome missegregation [458]. Interestingly, both CENP-E inhibitors PF-2771 and GSK923295 were found to increase CIN levels in a recent large-scale screen [459]. Taken together, these data suggest that CENP-E may be an effective therapeutic target for cancer cells with high levels of CIN. 

Other compounds have been claimed to specifically inhibit CENP-E, but turned out to target other proteins. For instance, the compound UA62784 was initially described to be a specific inhibitor of the ATPase activity of CENP-E and highly cytotoxic against human pancreatic cancer cell lines with a deletion of the DPC4 gene [460]. However, a subsequent study demonstrated that this compound does not exert its cellular activity by inhibiting CENP-E and rather binds microtubules tightly [461,462]. Another study that tested the antitumor activity of UA62784 and 80 analogs against pancreatic cancer cell lines revealed that these compounds potently inhibit several protein kinases that are overexpressed in these cancer cells, but not mitotic Kinesins (Kinesin-5, CENP-E, MKLP-1, and MCAK) [463]. Another compound, Syntelin, was also reported to be a highly selective CENP-E inhibitor [464]. Inhibition of CENP-E by Syntelin caused misaligned chromosomes with syntelic attachments, in which sister kinetochores stably attached to microtubules near the same spindle pole [464]. This was surprising, since perturbation of CENP-E produces polar chromosomes that are mostly devoid of microtubules at kinetochores [6,297,298], suggesting that Syntelin also targets other proteins (e.g., Aurora B).

To date, only one of the CENP-E inhibitors, GSK923295, has been evaluated in a Phase I clinical trial [465]. In this trial, peripheral neuropathy, a well-known taxane adverse effect, was not evident. As such, the use of CENP-E inhibitors as anticancer drugs could be better tolerated than taxanes and possibly easier to use in combination with other cancer therapies. Thus, better understanding of the molecular mechanisms behind CENP-E inhibition might help to find optimal clinical strategies for certain human cancers.

### 4.2. Farnesyltransferase Inhibitors (FTIs)

FTIs are promising agents for therapeutic intervention in several diseases, including cancer, malaria and progeria [466,467,468,469,470,471,472,473]. Due to the clinical relevance of these drugs it became important the identification of the cellular substrates of the farnesyltransferase. There are several proteins that are prone to be farnesylated [474] and several studies have shown that FTIs prevent the farnesylation of Ras family and some mitotic proteins involved in chromosome congression (such as CENP-E, CENP-F and Spindly) [327,334,335,475,476]. Since farnesylation is required for the recruitment of Ras proteins to the plasma membrane and many tumors exhibit mutations in Ras, FTIs were initially developed as therapeutic agents that target Ras activity in cancer cells [477]. Indeed, FTIs exhibited a potent inhibitory effect on the proliferation and invasive capabilities of breast cancer cells with active H-Ras in culture [478]. However, it became evident that the target of FTIs might not be only Ras proteins [479], and there was some evidence that FTIs demonstrated activity in cancer cells irrespective of Ras mutations [330,480,481,482]. Moreover, some studies have shown that treatment of different cancer cells with FTIs enhanced the anti-proliferative and apoptotic effects of cisplatin [483], 5-fluorouracil [484], MEK inhibitors [485], Cdk inhibitors [486], mTOR inhibitor (rapamycin) [487] and taxol [488]. Finally, and most relevant for our purposes, FTIs were shown to affect bipolar spindle assembly and chromosome congression [328,329,335]. 

Some FTIs, such as Tipifarnib (or R115777), Lonafarnib (or SCH66336), BMS-214662, L-778123 and SCH44342 are currently in clinical trials for the treatment of various solid tumors and hematological malignancies [471,489,490,491,492,493]. Although FTIs have been extensively tested in the clinics, their mechanism of cytotoxicity is not fully understood. In some clinical trials, treatment with FTIs alone or in combination with chemotherapeutic agents failed to improve the overall outcome of patients with solid tumors and leukemia [494,495,496,497,498,499,500,501]. However, other clinical trials demonstrated that the combination of FTIs with conventional chemotherapeutic agents might be useful in hematologic and some solid tumors [502,503,504,505,506,507,508]. Moreover, patients with poor-risk acute myeloid leukemia may benefit from FTIs maintenance therapy following cytotoxic induction and consolidation therapies [509]. Understanding the mechanisms by which these drugs inhibit cell proliferation and induce cell death might facilitate the development of new therapeutic strategies.

### 4.3. Inhibitors of Tubulin PTMs

The levels of various tubulin PTMs, including acetylation, detyrosination, Δ2 deglutamylation, polyglutamylation and glycylation, are altered in different cancer cell lines and tissues, contributing to tumor growth and enhancing their metastatic potential [510,511,512,513,514,515,516,517,518,519,520,521]. α-tubulin acetylation and detyrosination are increased in breast cancer cells and correlate with tumor aggressiveness and poor prognosis in patients [511,517]. A balance of tubulin acetylation and deacetylation by α-TAT1 and HDAC6 enzymes with opposite activities was proposed to regulate the migratory and invasive capacities of breast tumor cells [510]. Low expression of TTL, the enzyme responsible for tubulin retyrosination, leads to increased microtubule detyrosination and is correlated with inhibition of neuronal differentiation and increased cell growth in neuroblastoma with poor prognosis [518]. TTL expression was found to be suppressed during tumor growth in mice [516], as well as during epithelial-to-mesenchymal transition in human mammary epithelial cells in vitro [521], implicating the tubulin tyrosination cycle in both tumor propagation and metastasis. Such highly acetylated and detyrosinated microtubules can indeed form microtentacle protrusions that enhance cellular invasive migration and re-attachment [511,517]. Experimental microtubule deacetylation, achieved by mutating the α-tubulin acetylation site at Lysine 40, decreased the incidence of microtentacles and inhibited cellular migration and invasiveness, confirming the interdependence between cancer progression and tubulin PTMs [511].

Because of their correlation with cancer, tubulin PTMs present a very promising target for novel therapeutic approaches in human cancers. One of the most obvious strategies would rely on the pharmacological inhibition of the enzymes responsible for tubulin PTMs. A promising group of potential anti-cancer drugs that target tubulin detyrosination are sesquiterpene lactones, a series of bioactive compounds isolated from the Asteraceae family of plants [522]. The most studied compound is parthenolide, which has already been used in cancer clinical trials [523,524] and suppresses several different steps within the nuclear factor kappa B (NF-κB) signaling pathway [525,526,527,528]. In addition, parthenolide prevents microtubule detyrosination by inhibiting TCP, independently from its effect on NF-κB [529]. Therefore, parthenolide-mediated targeting of TCP and microtubule detyrosination might have a preventive effect on tumor growth, aneuploidy and metastasis, independently from its interference with the NF-κB pathway. Indeed, parthenolide-mediated suppression of cell invasiveness and re-attachment of breast cancer metastatic cells was shown to be independent of NF-κB [530]. Interestingly, several studies reported that various sesquiterpene lactones induced a G2 or M arrest [531,532,533], which might account for their anti-cancer activity. More recently, the effect of parthenolide over TCP inhibition was found to cause chromosome congression defects during mitosis [345,346], reinforcing the potential of targeting chromosome congression for cancer therapy.

The great advantage of parthenolide as an anti-cancer drug is that it appears to selectively target cancer cells, as documented by several different in vitro studies [524]. Moreover, parthenolide was the first small molecule shown to selectively kill cancer stem cells, while leaving normal stem cells intact [524,534]. This is of enormous therapeutic importance, since the presence of cancer stem cells is considered as one of the main reasons underlying chemotherapy resistance and tumor relapse due to their capacity of self-renewal and differentiation into multiple cell types [535,536]. The mechanism behind parthenolide selectivity towards cancer stem cells is not completely understood, but it is believed that the reason lies in its ability to target multiple major pathways required for cancer stem cell survival and self-renewal, such as MAPK, JAK/STAT, PI3K and NF-κB signaling [524,537]. Whether TCP inhibition by parthenolide contributes to cancer stem cell eradication remains to be elucidated.

The biggest disadvantage of parthenolide as a therapeutic drug is its high hydrophobicity, which limits its bioavailability for oral usage and solubility in plasma [523]. This is partially circumvented by the synthesis of a more water-soluble analog dimethylamino-parthenolide (DMAPT), which possesses an increased oral bioavailability [524]. DMAPT has already proved effective in selective eradication of human acute myeloid leukemia primary cultured stem cells [538] and breast cancer stem-like cultured cells [539], and has been shown to inhibit tumor growth and metastasis of prostate, lung and bladder cancer xenografts in mice [531,540]. However, although parthenolide and DMAPT demonstrated high potential in prevention of metastasis and treatment of cancer stem cells, they were not able to reduce tumor volumes. In contrast, radiotherapy and more conventional chemotherapeutic drugs, including the microtubule poisons taxanes, are able to reduce tumor volume, but usually fail to target cancer stem cells. Therefore, a therapy that includes radiotherapy or conventional chemotherapeutics, in combination with parthenolide/DMAPT could simultaneously target all types of cancer cells. Indeed, a synergistic effect of parthenolide in combination with either taxanes [541,542] or vinca alkaloids [543] was observed in breast cancer xenograft models in mice, affecting both tumor cells and cancer stem cells, while preventing metastasis. The development of new drugs that more specifically target enzymes that account for tubulin PTMs might reveal useful in evaluating potential clinical applications in the future. 

## 5. Conclusions and Future Perspectives

Overall, we conclude that chromosome congression in mammalian cells relies on the concerted action of motor-dependent and -independent mechanisms, which are determined by the establishment of end-on or lateral kinetochore-microtubule interactions. Therefore, any perturbation that introduces alterations of microtubule dynamics or kinetochore function will likely compromise the congression of at least some chromosomes during mitosis. In addition, the recent discovery that tubulin PTMs have an impact on kinetochore motors and might work as a navigation system during chromosome congression brings together two old research fields, while opening up new and exciting avenues for investigation in the future. To date, more than 100 proteins have been implicated in chromosome alignment (Table 1), but their exact role in the activities necessary for either congression or maintenance of alignment remains unknown for >90% of them. A systematic analysis of the respective role of these proteins in chromosome congression will be an important challenge for future studies of mitosis. Moreover, the functional relationship between forces involved in chromosome congression and mitotic spindle architecture remains poorly understood and deserves further attention [415]. Finally, it will be important to firmly establish whether problems in chromosome congression are directly responsible for human diseases, such as cancer, and whether targeting chromosome congression represents a valid therapeutic approach.

## Figures and Tables

**Table 1:** Proteins that have been implicated in chromosome alignment.

Protein Name	Subcellular Localization	Misaligned Chromosomes/Chromatids	Chromosome Congression Defects (by Live Cell Imaging)	References
Astrin	Spindle pole; kinetochores	Yes	Yes	[147,148,149,150]
HICE1/HAUS8	Centrosome; mitotic spindle; spindle midzone; midbody	Yes	ND	[151]
Aurora A	Centrosome; central spindle	Yes	Yes	[152,153,154]
CENP-E	Kinetochore	Yes	Yes	[6,155,156,157]
CEP57	Centrosome	Yes	ND	[158]
Cep72	Centrosome	Yes	ND	[159]
Cep90	Centrosome; Pericentriolar satellites	Yes	ND	[160]
ChTOG	Centrosome; spindle pole	Yes	Yes	[112,150,161]
CLASPs	Centrosome; kinetochore; microtubule plus ends; central spindle	Yes	Yes	[150,162]
Aurora-B	Centromere; spindle; spindle midzone	Yes	Yes	[163,164]
Haspin	Chromosome; centrosome	Yes	Yes	[165,166,167]
ILK	Plasma membrane; focal adhesion; cytosol	Yes	ND	[168]
Kinastrin/SKAP	Spindle pole; kinetochore; microtubule plus ends	Yes	yes	[148,149,169]
HEC1	Kinetochore	Yes	Yes	[170,171,172,173]
Spc24	Kinetochore	Yes	ND	[174]
Spc25	Kinetochore	Yes	ND	[174]
Nuf2	Kinetochore	Yes	Yes	[174,175]
NuMA	Nucleus; spindle pole	Yes	ND	[176]
Sgo1/Shugoshin	Centromere; kinetochore; centrosome; spindle pole	Yes	Yes	[177]
Spindly	Kinetochore; spindle pole	Yes	Yes	[178,179]
TACC3	Centrosome	Yes	Yes	[161,180,181,182]
CHC (Clathrin heavy chain)	Mitotic spindle	Yes	Yes	[181,183]
4.1r	Mature centriole	Yes	ND	[184]
Ska1	Kinetochore; mitotic spindle	Yes	Yes	[185,186,187,188]
Ska2	Kinetochore; mitotic spindle	Yes	Yes	[185,186,187,188]
Ska3/RAMA1	Kinetochore; mitotic spindle	Yes	Yes	[186,187,188,189,190]
Kid	Chromosome arms; spindle poles	Yes	Yes	[70,71,191]
Kif4A	Chromosome arms; spindle midzone	Yes	Yes	[69,70,71]
Kif18A	Plus-ends of kMTs	Yes	Yes	[134,144,146,192,193]
Kif18B	Astral microtubule plus ends	Yes	Yes	[194,195,196]
MCAK	Spindle poles; spindle midzone; kinetochore	Yes	Yes	[70,124,197]
HURP	Kinetochore	Yes	Yes	[198,199,200]
CENP-L	Kinetochore	Yes	Yes	[201]
NuSAP1	Central spindle	Yes	Yes	[202,203]
SAF-A/hnRNP-U	Spindle microtubules; spindle midzone	Yes	Yes	[204]
Bub1	Kinetochore	Yes	Yes	[164,205]
BubR1	Kinetochore	Yes	Yes	[164,206,207,208]
NUP188	Centrosomes	Yes	Yes	[209]
CENP-F/mitosin	Kinetochore	Yes	Yes	[210,211,212]
Plk1	Centrosome	Yes	Yes	[213,214,215]
NudC	Kinetochore	Yes	Yes	[216,217]
RRS1	Chromosome periphery	Yes	Yes	[218]
Nucleolin	Nucleoli; chromosome periphery	Yes	Yes	[219]
KIBRA	ND	Yes	ND	[220]
DDA3	Spindle microtubules; kinetochores; midbody	Yes	Yes	[221,222]
HIP1r	Mitotic spindle	Yes	Yes	[223]
Nucleophosmin	Perichromosomal region	Yes	Yes	[224]
Kif2a	Spindle poles	Yes	Yes	[124,221]
Beclin-1	Kinetochore	Yes	Yes	[225]
CLIP-170	Kinetochore; mitotic spindle	Yes	Yes	[104,106]
ATRX	Pericentromeric heterochromatin	Yes	Yes	[226]
CHICA	Mitotic spindle	Yes	Yes	[227,228]
p38γ	Kinetochore; spindle poles	Yes	Yes	[229]
SPICE	Mitotic spindle; centrioles	Yes	Yes	[230]
Zw10	Kinetochore	Yes	Yes	[231,232]
DHC/DYNC1H1	Kinetochore; mitotic spindle	Yes	Yes	[6,178]
DIC2/DYNC1I2	Kinetochore; mitotic spindle	Yes	Yes	[178]
Roadblock-1/DYNLRB1	Kinetochore; mitotic spindle	Yes	Yes	[178]
Lis1/PAFAH1B1	Kinetochore; mitotic spindle	Yes	Yes	[178]
Nde1	Kinetochore; mitotic spindle	Yes	Yes	[178]
Ndel1	Kinetochore; mitotic spindle	Yes	Yes	[178]
ARP1	Kinetochore; mitotic spindle	Yes	Yes	[178]
TAO1/MARKK	Microtubules	Yes	Yes	[233]
Kif14	Spindle poles; mitotic spindle; midbody	Yes	Yes	[70,234]
CENP-W	Kinetochore	yes	yes	[235,236,237]
CENP-T	Kinetochore	Yes	ND	[235,238]
CENP-H	Kinetochore	Yes	Yes	[239]
Chl4r	Kinetochore	Yes	Yes	[239]
Nnf1R	Kinetochore	Yes	Yes	[239,240]
CENP-Q	Kinetochore	Yes	Yes	[241]
CENP-U	Kinetochore	Yes	Yes	[238,242]
CENP-N	Kinetochore	Yes	ND	[238]
CENP-M	Kinetochore	Yes	ND	[238,243]
Septin 7	Spindle poles; mitotic spindle; midbody	Yes	ND	[244]
TRAMM	Perinuclear region	Yes	Yes	[245]
Shp2	Kinetochore; centrosome; spindle midzone; midbody	Yes	Yes	[246,247]
Bod1	Centrosomes; kinetochores	Yes	Yes	[248,249]
PTEN	Centrosome; mitotic spindle; midbody	Yes	Yes	[250]
RSK2/RPS6KA3	Centrosomes; mitotic spindle; midbody; kinetochore	Yes	Yes	[251,252,253]
Nup62	Nuclear envelope; cytoplasm; centrosomes	Yes	ND	[254,255]
Mdp3	Mitotic spindle	Yes	Yes	[256]
ANKRD53	Spindle poles	Yes	Yes	[257]
NF-1 (neurofibromatosis type 1)	Astral microtubules; mitotic spindle; centrosomes; midbody	Yes	ND	[258]
Hsp72	Mitotic spindle; midbody	Yes	Yes	[259]
RGS2	Centrosome; mitotic spindle; astral microtubules	Yes	ND	[260]
B56	Centromere	Yes	Yes	[174,207,261,262]
And-1 (acidic nucleoplasmic DNA-binding protein 1)	Cytoplasm	Yes	ND	[263]
ASURA (PHB2)	Cytoplasm	Yes	ND	[264]
Rab5	Early endosomes	Yes	Yes	[211]
MST1	ND	Yes	Yes	[265]
GAK	Trans-Golgi network	Yes	ND	[266]
Usp16	Cytoplasmic in interphase; kinetochore	Yes	Yes	[267]
TTL	Mitotic spindle	Yes	Yes	[345]
TCP	ND	Yes	Yes	[345]

ND (not determined).

## Abbreviations

The following abbreviations are used in this manuscript:

SAC	Spindle Assembly Checkpoint
UV	Ultra-violet
PEF	Polar Ejection Force
GFPMUGs	Green Fluorescent ProteinMitosis with Unreplicated Genomes
+TIPs	Microtubule Plus-End-Tracking Proteins
ATP	Adenosine Triphosphate
RNAi	RNA interference
MAPs	Microtubule-Associated Proteins
NEB	Nuclear Envelope Breakdown
PTMs	Post-Translational Modifications
TCP	Tubulin Carboxypeptidase
TTL	Tubulin Tyrosine Ligase
aMTOCs	acentriolar microtubule-organizing centers
CIN	Chromosomal Instability
MPD	Microcephalic primordial dwarfism
CAC	Colitis-Associated Cancer
DMAPT	Dimethylamino-parthenolide

## References

[1] Darlington C.D. (1937). Recent Advances in Cytology.

[2] Straight A.F., Marshall W.F., Sedat J.W., Murray A.W. (1997). Mitosis in living budding yeast: Anaphase a but no metaphase plate. Science.

[3] Goshima G., Scholey J.M. (2010). Control of mitotic spindle length. Annu. Rev. Cell Dev. Biol..

[4] Matos I., Pereira A.J., Lince-Faria M., Cameron L.A., Salmon E.D., Maiato H. (2009). Synchronizing chromosome segregation by flux-dependent force equalization at kinetochores. J. Cell Biol..

[5] Joglekar A.P. (2016). A cell biological perspective on past, present and future investigations of the spindle assembly checkpoint. Biology.

[6] Barisic M., Aguiar P., Geley S., Maiato H. (2014). Kinetochore motors drive congression of peripheral polar chromosomes by overcoming random arm-ejection forces. Nat. Cell Biol..

[7] Ye A.A., Deretic J., Hoel C.M., Hinman A.W., Cimini D., Welburn J.P., Maresca T.J. (2015). Aurora A Kinase Contributes to a Pole-Based Error Correction Pathway. Curr. Biol..

[8] Chmatal L., Yang K., Schultz R.M., Lampson M.A. (2015). Spatial Regulation of Kinetochore Microtubule Attachments by Destabilization at Spindle Poles in Meiosis I. Curr. Biol..

[9] King J.M., Nicklas R.B. (2000). Tension on chromosomes increases the number of kinetochore microtubules but only within limits. J. Cell Sci..

[10] Wilson E.B. (1925). The Cell in Development and Heredity.

[11] Lawrence W.J.C. (1931). The genetics and cytology of Dahlia variabilis. J. Genet..

[12] Drüner L. (1895). Studien über den mechanismus der zellteilung. Jenaische Ztschr. Naturw..

[13] Östergren G. (1950). Considerations on some elementary features of mitosis. Hereditas.

[14] Belar K. (1929). Beiträge zur kausalanalyse der mitose II. Arch. Entwickl..

[15] Rashevsky N. (1941). Some remarks on the movement of chromosomes during cell division. Bull. Math. Biophys..

[16] Wada B. (1950). The mechanism of mitosis based on studies of the submicroscopic structure and of the living state of the Tradescantia cell. Cytologia.

[17] Östergren G. (1945). Equilibrium of trivalents and the mechanism of chromosome movements. Hereditas.

[18] Schrader F. (1953). Mitosis—The Movements of Chromosomes in Cell Division.

[19] Böök J.A. (1945). Equilibrium of trivalents at metaphase. Hereditas.

[20] Izutsu K. (1959). Irradiation of parts of single mitotic apparatus in grasshopper spermatocytes with an ultraviolet-microbeam. Mie Med. J..

[21] Takeda S., Izutsu K. (1960). Partial irradiation of individual mitotic cells with ultraviolet microbeam. Symposia Cell Chem..

[22] Izutsu K. (1961). Effects of ultraviolet microbeam irradiation upon division in grasshoper spermatocytes. II. Results of irradiation during metaphase and anaphase I. Mie Med. J..

[23] McNeill P.A., Berns M.W. (1981). Chromosome behavior after laser microirradiation of a single kinetochore in mitotic PtK2 cells. J. Cell Biol..

[24] Hays T.S., Wise D., Salmon E.D. (1982). Traction force on a kinetochore at metaphase acts as a linear function of kinetochore fiber length. J. Cell Biol..

[25] Dietz R. (1972). Anaphase behaviour of inversions in living crane-fly spermatocytes. Chromosom. Today.

[26] Hays T.S., Salmon E.D. (1990). Poleward force at the kinetochore in metaphase depends on the number of kinetochore microtubules. J. Cell Biol..

[27] LaFountain J.R., Oldenbourg R. (2004). Maloriented bivalents have metaphase positions at the spindle equator with more kinetochore microtubules to one pole than to the other. Mol. Biol. Cell.

[28] McEwen B.F., Heagle A.B., Cassels G.O., Buttle K.F., Rieder C.L. (1997). Kinetochore fiber maturation in PtK1 cells and its implications for the mechanisms of chromosome congression and anaphase onset. J. Cell Biol..

[29] Maiato H., Rieder C.L., Khodjakov A. (2004). Kinetochore-driven formation of kinetochore fibers contributes to spindle assembly during animal mitosis. J. Cell Biol..

[30] Forer A. (1965). Local Reduction of Spindle Fiber Birefringence in Living Nephrotoma Suturalis (Loew) Spermatocytes Induced by Ultraviolet Microbeam Irradiation. J. Cell Biol..

[31] Inoue S. (1964). Organization and function of the mitotic spindle. Primitive Motile Systems in Cell Biology.

[32] Spurck T.P., Stonington O.G., Snyder J.A., Pickett-Heaps J.D., Bajer A., Mole-Bajer J. (1990). UV microbeam irradiations of the mitotic spindle. II. Spindle fiber dynamics and force production. J. Cell Biol..

[33] Nicklas R.B. (1989). The motor for poleward chromosome movement in anaphase is in or near the kinetochore. J. Cell Biol..

[34] Czaban B.B., Forer A., Bajer A.S. (1993). Ultraviolet microbeam irradiation of chromosomal spindle fibres in Haemanthus katherinae endosperm. I. Behaviour of the irradiated region. J. Cell Sci..

[35] Sikirzhytski V., Magidson V., Steinman J.B., He J., Le Berre M., Tikhonenko I., Ault J.G., McEwen B.F., Chen J.K., Sui H. (2014). Direct kinetochore-spindle pole connections are not required for chromosome segregation. J. Cell Biol..

[36] Elting M.W., Hueschen C.L., Udy D.B., Dumont S. (2014). Force on spindle microtubule minus ends moves chromosomes. J. Cell Biol..

[37] Kajtez J., Solomatina A., Novak M., Polak B., Vukusic K., Rudiger J., Cojoc G., Milas A., Sumanovac Sestak I., Risteski P. (2016). Overlap microtubules link sister k-fibres and balance the forces on bi-oriented kinetochores. Nat. Commun..

[38] Milas A., Tolic I.M., Zür M. (2016). Relaxation of interkinetochore tension after severing of a k-fiber depends on the length of the k-fiber stub. Matters.

[39] Bajer A.S., Molè-Bajer J. (1972). Spindle dynamics and chromosome movements. Int. Rev. Cytol..

[40] Luykx P. (1970). Cellular mechanisms of chromosome distribution. Int. Rev. Cytol..

[41] Bajer A.S. (1982). Functional autonomy of monopolar spindle and evidence for oscillatory movement in mitosis. J. Cell Biol..

[42] Molè-Bajer J., Bajer A., Owczarzak A. (1975). Chromosome movements in prometaphase and aster transport in the newt. Cytobios.

[43] Rieder C.L., Davison E.A., Jensen L.C., Cassimeris L., Salmon E.D. (1986). Oscillatory movements of monooriented chromosomes and their position relative to the spindle pole result from the ejection properties of the aster and half-spindle. J. Cell Biol..

[44] Salmon E.D., Hyams J.S., Brinkley B.R. (1989). Microtubule dynamics and chromosome movement. Mitosis: Molecules and Mechanisms.

[45] Ault J.G., DeMarco A.J., Salmon E.D., Rieder C.L. (1991). Studies on the ejection properties of asters: Astral microtubule turnover influences the oscillatory behavior and positioning of mono-oriented chromosomes. J. Cell Sci..

[46] Cassimeris L., Rieder C.L., Salmon E.D. (1994). Microtubule assembly and kinetochore directional instability in vertebrate monopolar spindles: Implications for the mechanism of chromosome congression. J. Cell Sci..

[47] Salmon E.D., Warner F.D., McIntosh J.R. (1989). Metaphase chromosome congression and anaphase poleward movement. Cell Movement: Kinesin, Dynein and Microtubule Dynamics.

[48] Khodjakov A., Rieder C.L. (1996). Kinetochores moving away from their associated pole do not exert a significant pushing force on the chromosome. J. Cell Biol..

[49] Waters J.C., Skibbens R.V., Salmon E.D. (1996). Oscillating mitotic newt lung cell kinetochores are, on average, under tension and rarely push. J. Cell Sci..

[50] Ke K., Cheng J., Hunt A.J. (2009). The distribution of polar ejection forces determines the amplitude of chromosome directional instability. Curr. Biol..

[51] Rieder C.L., Salmon E.D. (1994). Motile kinetochores and polar ejection forces dictate chromosome position on the vertebrate mitotic spindle. J. Cell Biol..

[52] Bajer A.S., Cypher C., Mole-Bajer J., Howard H.M. (1982). Taxol-induced anaphase reversal: Evidence that elongating microtubules can exert a pushing force in living cells. Proc. Natl. Acad. Sci. USA.

[53] Dogterom M., Yurke B. (1997). Measurement of the force-velocity relation for growing microtubules. Science.

[54] Fygenson D.K., Marko J.F., Libchaber A. (1997). Mechanics of microtubule-based membrane extension. Phys. Rev. Lett..

[55] Marshall W.F., Marko J.F., Agard D.A., Sedat J.W. (2001). Chromosome elasticity and mitotic polar ejection force measured in living Drosophila embryos by four-dimensional microscopy-based motion analysis. Curr. Biol..

[56] Brouhard G.J., Hunt A.J. (2005). Microtubule movements on the arms of mitotic chromosomes: Polar ejection forces quantified in vitro. Proc. Natl. Acad. Sci. USA.

[57] Kuo S.C., Sheetz M.P. (1993). Force of single kinesin molecules measured with optical tweezers. Science.

[58] Svoboda K., Block S.M. (1994). Force and velocity measured for single kinesin molecules. Cell.

[59] Hall K., Cole D., Yeh Y., Baskin R.J. (1996). Kinesin force generation measured using a centrifuge microscope sperm-gliding motility assay. Biophys. J..

[60] Carpenter A.T. (1991). Distributive segregation: Motors in the polar wind?. Cell.

[61] Wang S.Z., Adler R. (1995). Chromokinesin: A DNA-binding, kinesin-like nuclear protein. J. Cell Biol..

[62] Vernos I., Raats J., Hirano T., Heasman J., Karsenti E., Wylie C. (1995). Xklp1, a chromosomal Xenopus kinesin-like protein essential for spindle organization and chromosome positioning. Cell.

[63] Vanneste D., Ferreira V., Vernos I. (2011). Chromokinesins: Localization-dependent functions and regulation during cell division. Biochem. Soc. Trans..

[64] Theurkauf W.E., Hawley R.S. (1992). Meiotic spindle assembly in Drosophila females: Behavior of nonexchange chromosomes and the effects of mutations in the nod kinesin-like protein. J. Cell Biol..

[65] Funabiki H., Murray A.W. (2000). The Xenopus chromokinesin Xkid is essential for metaphase chromosome alignment and must be degraded to allow anaphase chromosome movement. Cell.

[66] Antonio C., Ferby I., Wilhelm H., Jones M., Karsenti E., Nebreda A.R., Vernos I. (2000). Xkid, a chromokinesin required for chromosome alignment on the metaphase plate. Cell.

[67] Levesque A.A., Compton D.A. (2001). The chromokinesin Kid is necessary for chromosome arm orientation and oscillation, but not congression, on mitotic spindles. J. Cell Biol..

[68] Goshima G., Vale R.D. (2003). The roles of microtubule-based motor proteins in mitosis: Comprehensive RNAi analysis in the Drosophila S2 cell line. J. Cell Biol..

[69] Mazumdar M., Sundareshan S., Misteli T. (2004). Human chromokinesin KIF4A functions in chromosome condensation and segregation. J. Cell Biol..

[70] Zhu C., Zhao J., Bibikova M., Leverson J.D., Bossy-Wetzel E., Fan J.B., Abraham R.T., Jiang W. (2005). Functional analysis of human microtubule-based motor proteins, the kinesins and dyneins, in mitosis/cytokinesis using RNA interference. Mol. Biol. Cell.

[71] Wandke C., Barisic M., Sigl R., Rauch V., Wolf F., Amaro A.C., Tan C.H., Pereira A.J., Kutay U., Maiato H. (2012). Human chromokinesins promote chromosome congression and spindle microtubule dynamics during mitosis. J. Cell Biol..

[72] Magidson V., O’Connell C.B., Loncarek J., Paul R., Mogilner A., Khodjakov A. (2011). The spatial arrangement of chromosomes during prometaphase facilitates spindle assembly. Cell.

[73] Sekine Y., Okada Y., Noda Y., Kondo S., Aizawa H., Takemura R., Hirokawa N. (1994). A novel microtubule-based motor protein (KIF4) for organelle transports, whose expression is regulated developmentally. J. Cell Biol..

[74] Bringmann H., Skiniotis G., Spilker A., Kandels-Lewis S., Vernos I., Surrey T. (2004). A kinesin-like motor inhibits microtubule dynamic instability. Science.

[75] Yajima J., Edamatsu M., Watai-Nishii J., Tokai-Nishizumi N., Yamamoto T., Toyoshima Y.Y. (2003). The human chromokinesin Kid is a plus end-directed microtubule-based motor. EMBO J..

[76] Bieling P., Kronja I., Surrey T. (2010). Microtubule motility on reconstituted meiotic chromatin. Curr. Biol..

[77] Cane S., Ye A.A., Luks-Morgan S.J., Maresca T.J. (2013). Elevated polar ejection forces stabilize kinetochore-microtubule attachments. J. Cell Biol..

[78] Skibbens R.V., Skeen V.P., Salmon E.D. (1993). Directional instability of kinetochore motility during chromosome congression and segregation in mitotic newt lung cells: A push-pull mechanism. J. Cell Biol..

[79] Nicklas R.B., Koch C.A. (1969). Chromosome micromanipulation. 3. Spindle fiber tension and the reorientation of mal-oriented chromosomes. J. Cell Biol..

[80] Nicklas R.B., Ward S.C. (1994). Elements of error correction in mitosis: Microtubule capture, release, and tension. J. Cell Biol..

[81] Drpic D., Pereira A.J., Barisic M., Maresca T.J., Maiato H. (2015). Polar Ejection Forces Promote the Conversion from Lateral to End-on Kinetochore-Microtubule Attachments on Mono-oriented Chromosomes. Cell Rep..

[82] Maresca T.J., Salmon E.D. (2009). Intrakinetochore stretch is associated with changes in kinetochore phosphorylation and spindle assembly checkpoint activity. J. Cell Biol..

[83] Uchida K.S., Takagaki K., Kumada K., Hirayama Y., Noda T., Hirota T. (2009). Kinetochore stretching inactivates the spindle assembly checkpoint. J. Cell Biol..

[84] Magidson V., He J., Ault J.G., O’Connell C.B., Yang N., Tikhonenko I., McEwen B.F., Sui H., Khodjakov A. (2016). Unattached kinetochores rather than intrakinetochore tension arrest mitosis in taxol-treated cells. J. Cell Biol..

[85] Inoue S. (1952). The effect of colchicine on the microscopic and submicroscopic structure of the mitotic spindle. Exp. Cell Res..

[86] Inoue S., Salmon E.D. (1995). Force generation by microtubule assembly/disassembly in mitosis and related movements. Mol. Biol. Cell.

[87] Koshland D.E., Mitchison T.J., Kirschner M.W. (1988). Polewards chromosome movement driven by microtubule depolymerization in vitro. Nature.

[88] Coue M., Lombillo V.A., McIntosh J.R. (1991). Microtubule depolymerization promotes particle and chromosome movement in vitro. J. Cell Biol..

[89] Grishchuk E.L., Molodtsov M.I., Ataullakhanov F.I., McIntosh J.R. (2005). Force production by disassembling microtubules. Nature.

[90] Cassimeris L., Salmon E.D. (1991). Kinetochore microtubules shorten by loss of subunits at the kinetochores of prometaphase chromosomes. J. Cell Sci..

[91] Tirnauer J.S., Canman J.C., Salmon E.D., Mitchison T.J. (2002). EB1 targets to kinetochores with attached, polymerizing microtubules. Mol. Biol. Cell.

[92] VandenBeldt K.J., Barnard R.M., Hergert P.J., Meng X., Maiato H., McEwen B.F. (2006). Kinetochores use a novel mechanism for coordinating the dynamics of individual microtubules. Curr. Biol..

[93] Armond J.W., Vladimirou E., Erent M., McAinsh A.D., Burroughs N.J. (2015). Probing microtubule polymerisation state at single kinetochores during metaphase chromosome motion. J. Cell Sci..

[94] Cheeseman I.M., Desai A. (2008). Molecular architecture of the kinetochore-microtubule interface. Nat. Rev. Mol. Cell Biol..

[95] Schuyler S.C., Pellman D. (2001). Microtubule “plus-end-tracking proteins”: The end is just the beginning. Cell.

[96] Akhmanova A., Steinmetz M.O. (2010). Microtubule +TIPs at a glance. J. Cell Sci..

[97] Akhmanova A., Steinmetz M.O. (2008). Tracking the ends: A dynamic protein network controls the fate of microtubule tips. Nat. Rev. Mol. Cell Biol..

[98] Brouhard G.J., Stear J.H., Noetzel T.L., Al-Bassam J., Kinoshita K., Harrison S.C., Howard J., Hyman A.A. (2008). XMAP215 is a processive microtubule polymerase. Cell.

[99] Komarova Y.A., Akhmanova A.S., Kojima S., Galjart N., Borisy G.G. (2002). Cytoplasmic linker proteins promote microtubule rescue in vivo. J. Cell Biol..

[100] Mimori-Kiyosue Y., Grigoriev I., Lansbergen G., Sasaki H., Matsui C., Severin F., Galjart N., Grosveld F., Vorobjev I., Tsukita S. (2005). CLASP1 and CLASP2 bind to EB1 and regulate microtubule plus-end dynamics at the cell cortex. J. Cell Biol..

[101] Akhmanova A., Hoogenraad C.C., Drabek K., Stepanova T., Dortland B., Verkerk T., Vermeulen W., Burgering B.M., De Zeeuw C.I., Grosveld F. (2001). Clasps are CLIP-115 and -170 associating proteins involved in the regional regulation of microtubule dynamics in motile fibroblasts. Cell.

[102] Perez F., Diamantopoulos G.S., Stalder R., Kreis T.E. (1999). CLIP-170 highlights growing microtubule ends in vivo. Cell.

[103] Dujardin D., Wacker U.I., Moreau A., Schroer T.A., Rickard J.E., De Mey J.R. (1998). Evidence for a role of CLIP-170 in the establishment of metaphase chromosome alignment. J. Cell Biol..

[104] Tanenbaum M.E., Galjart N., van Vugt M.A.T.M., Medema R.H. (2006). CLIP-170 facilitates the formation of kinetochore-microtubule attachments. EMBO J..

[105] Kakeno M., Matsuzawa K., Matsui T., Akita H., Sugiyama I., Ishidate F., Nakano A., Takashima S., Goto H., Inagaki M. (2014). Plk1 phosphorylates CLIP-170 and regulates its binding to microtubules for chromosome alignment. Cell Struct. Funct..

[106] Amin M.A., Kobayashi K., Tanaka K. (2015). CLIP-170 tethers kinetochores to microtubule plus ends against poleward force by dynein for stable kinetochore-microtubule attachment. FEBS Lett..

[107] Bonfils C., Bec N., Lacroix B., Harricane M.C., Larroque C. (2007). Kinetic analysis of tubulin assembly in the presence of the microtubule-associated protein TOGp. J. Biol. Chem..

[108] Gard D.L., Kirschner M.W. (1987). A microtubule-associated protein from Xenopus eggs that specifically promotes assembly at the plus-end. J. Cell Biol..

[109] Al-Bassam J., Chang F. (2011). Regulation of microtubule dynamics by TOG-domain proteins XMAP215/Dis1 and CLASP. Trends Cell Biol..

[110] Al-Bassam J., Kim H., Brouhard G., van Oijen A., Harrison S.C., Chang F. (2010). CLASP promotes microtubule rescue by recruiting tubulin dimers to the microtubule. Dev. Cell.

[111] Gandhi S.R., Gierlinski M., Mino A., Tanaka K., Kitamura E., Clayton L., Tanaka T.U. (2011). Kinetochore-dependent microtubule rescue ensures their efficient and sustained interactions in early mitosis. Dev. Cell.

[112] Gergely F., Draviam V.M., Raff J.W. (2003). The ch-TOG/XMAP215 protein is essential for spindle pole organization in human somatic cells. Genes Dev..

[113] Kitamura E., Tanaka K., Komoto S., Kitamura Y., Antony C., Tanaka T.U. (2010). Kinetochores generate microtubules with distal plus ends: Their roles and limited lifetime in mitosis. Dev. Cell.

[114] Miller M.P., Asbury C.L., Biggins S. (2016). A TOG Protein Confers Tension Sensitivity to Kinetochore-Microtubule Attachments. Cell.

[115] Cassimeris L., Becker B., Carney B. (2009). TOGp regulates microtubule assembly and density during mitosis and contributes to chromosome directional instability. Cell Motil. Cytoskeleton.

[116] Maiato H., Fairley E.A., Rieder C.L., Swedlow J.R., Sunkel C.E., Earnshaw W.C. (2003). Human CLASP1 is an outer kinetochore component that regulates spindle microtubule dynamics. Cell.

[117] Pereira A.L., Pereira A.J., Maia A.R., Drabek K., Sayas C.L., Hergert P.J., Lince-Faria M., Matos I., Duque C., Stepanova T. (2006). Mammalian CLASP1 and CLASP2 cooperate to ensure mitotic fidelity by regulating spindle and kinetochore function. Mol. Biol. Cell.

[118] Maiato H., Khodjakov A., Rieder C.L. (2005). Drosophila CLASP is required for the incorporation of microtubule subunits into fluxing kinetochore fibres. Nat. Cell Biol..

[119] Maffini S., Maia A.R., Manning A.L., Maliga Z., Pereira A.L., Junqueira M., Shevchenko A., Hyman A., Yates J.R., Galjart N. (2009). Motor-independent targeting of CLASPs to kinetochores by CENP-E promotes microtubule turnover and poleward flux. Curr. Biol..

[120] Manning A.L., Bakhoum S.F., Maffini S., Correia-Melo C., Maiato H., Compton D.A. (2010). CLASP1, astrin and Kif2b form a molecular switch that regulates kinetochore-microtubule dynamics to promote mitotic progression and fidelity. Embo J..

[121] Maia A.R., Garcia Z., Kabeche L., Barisic M., Maffini S., Macedo-Ribeiro S., Cheeseman I.M., Compton D.A., Kaverina I., Maiato H. (2012). Cdk1 and Plk1 mediate a CLASP2 phospho-switch that stabilizes kinetochore-microtubule attachments. J. Cell Biol..

[122] Walczak C.E., Gayek S., Ohi R. (2013). Microtubule-depolymerizing kinesins. Annu. Rev. Cell Dev. Biol..

[123] Desai A., Verma S., Mitchison T.J., Walczak C.E. (1999). Kin I kinesins are microtubule-destabilizing enzymes. Cell.

[124] Manning A.L., Ganem N.J., Bakhoum S.F., Wagenbach M., Wordeman L., Compton D.A. (2007). The kinesin-13 proteins Kif2a, Kif2b, and Kif2c/MCAK have distinct roles during mitosis in human cells. Mol. Biol. Cell.

[125] Walczak C.E. (2003). The Kin I kinesins are microtubule end-stimulated ATPases. Mol. Cell.

[126] Ganem N.J., Compton D.A. (2004). The KinI kinesin Kif2a is required for bipolar spindle assembly through a functional relationship with MCAK. J. Cell Biol..

[127] Walczak C.E., Mitchison T.J., Desai A. (1996). XKCM1: A Xenopus kinesin-related protein that regulates microtubule dynamics during mitotic spindle assembly. Cell.

[128] Kline-Smith S.L., Walczak C.E. (2002). The microtubule-destabilizing kinesin XKCM1 regulates microtubule dynamic instability in cells. Mol. Biol. Cell.

[129] Wordeman L., Wagenbach M., von Dassow G. (2007). MCAK facilitates chromosome movement by promoting kinetochore microtubule turnover. J. Cell Biol..

[130] Bakhoum S.F., Thompson S.L., Manning A.L., Compton D.A. (2009). Genome stability is ensured by temporal control of kinetochore-microtubule dynamics. Nat. Cell Biol..

[131] Gaetz J., Kapoor T.M. (2004). Dynein/dynactin regulate metaphase spindle length by targeting depolymerizing activities to spindle poles. J. Cell Biol..

[132] Ganem N.J., Upton K., Compton D.A. (2005). Efficient mitosis in human cells lacking poleward microtubule flux. Curr. Biol..

[133] Gupta M.L., Carvalho P., Roof D.M., Pellman D. (2006). Plus end-specific depolymerase activity of Kip3, a kinesin-8 protein, explains its role in positioning the yeast mitotic spindle. Nat. Cell Biol..

[134] Mayr M.I., Hümmer S., Bormann J., Grüner T., Adio S., Woehlke G., Mayer T.U. (2007). The human kinesin Kif18A is a motile microtubule depolymerase essential for chromosome congression. Curr. Biol..

[135] Varga V., Helenius J., Tanaka K., Hyman A.A., Tanaka T.U., Howard J. (2006). Yeast kinesin-8 depolymerizes microtubules in a length-dependent manner. Nat. Cell Biol..

[136] Varga V., Leduc C., Bormuth V., Diez S., Howard J. (2009). Kinesin-8 motors act cooperatively to mediate length-dependent microtubule depolymerization. Cell.

[137] Du Y., English C.A., Ohi R. (2010). The kinesin-8 Kif18A dampens microtubule plus-end dynamics. Curr. Biol..

[138] Stumpff J., Du Y., English C.A., Maliga Z., Wagenbach M., Asbury C.L., Wordeman L., Ohi R. (2011). A tethering mechanism controls the processivity and kinetochore-microtubule plus-end enrichment of the kinesin-8 Kif18A. Mol. Cell.

[139] Gandhi R., Bonaccorsi S., Wentworth D., Doxsey S., Gatti M., Pereira A. (2004). The Drosophila kinesin-like protein KLP67A is essential for mitotic and male meiotic spindle assembly. Mol. Biol. Cell.

[140] Goshima G., Wollman R., Stuurman N., Scholey J.M., Vale R.D. (2005). Length control of the metaphase spindle. Curr. Biol..

[141] Rischitor P.E., Konzack S., Fischer R. (2004). The Kip3-like kinesin KipB moves along microtubules and determines spindle position during synchronized mitoses in Aspergillus nidulans hyphae. Eukaryotic Cell.

[142] Straight A.F., Sedat J.W., Murray A.W. (1998). Time-lapse microscopy reveals unique roles for kinesins during anaphase in budding yeast. J. Cell Biol..

[143] West R.R., Malmstrom T., McIntosh J.R. (2002). Kinesins klp5(+) and klp6(+) are required for normal chromosome movement in mitosis. J. Cell Sci..

[144] Stumpff J., von Dassow G., Wagenbach M., Asbury C., Wordeman L. (2008). The kinesin-8 motor Kif18A suppresses kinetochore movements to control mitotic chromosome alignment. Dev. Cell.

[145] Jaqaman K., King E.M., Amaro A.C., Winter J.R., Dorn J.F., Elliott H.L., McHedlishvili N., McClelland S.E., Porter I.M., Posch M. (2010). Kinetochore alignment within the metaphase plate is regulated by centromere stiffness and microtubule depolymerases. J. Cell Biol..

[146] Stumpff J., Wagenbach M., Franck A., Asbury C.L., Wordeman L. (2012). Kif18A and chromokinesins confine centromere movements via microtubule growth suppression and spatial control of kinetochore tension. Dev. Cell.

[147] Thein K.H., Kleylein-Sohn J., Nigg E.A., Gruneberg U. (2007). Astrin is required for the maintenance of sister chromatid cohesion and centrosome integrity. J. Cell Biol..

[148] Schmidt J.C., Kiyomitsu T., Hori T., Backer C.B., Fukagawa T., Cheeseman I.M. (2010). Aurora B kinase controls the targeting of the Astrin-SKAP complex to bioriented kinetochores. J. Cell Biol..

[149] Dunsch A.K., Linnane E., Barr F.A., Gruneberg U. (2011). The astrin-kinastrin/SKAP complex localizes to microtubule plus ends and facilitates chromosome alignment. J. Cell Biol..

[150] Logarinho E., Maffini S., Barisic M., Marques A., Toso A., Meraldi P., Maiato H. (2012). CLASPs prevent irreversible multipolarity by ensuring spindle-pole resistance to traction forces during chromosome alignment. Nat. Cell Biol..

[151] Wu G., Lin Y.-T., Wei R., Chen Y., Shan Z., Lee W.-H. (2008). Hice1, a novel microtubule-associated protein required for maintenance of spindle integrity and chromosomal stability in human cells. Mol. Cell. Biol..

[152] Hoar K., Chakravarty A., Rabino C., Wysong D., Bowman D., Roy N., Ecsedy J.A. (2007). MLN8054, a Small-Molecule Inhibitor of Aurora A, Causes Spindle Pole and Chromosome Congression Defects Leading to Aneuploidy. Mol. Cell. Biol..

[153] Sasai K., Parant J.M., Brandt M.E., Carter J., Adams H.P., Stass S.A., Killary A.M., Katayama H., Sen S. (2008). Targeted disruption of Aurora A causes abnormal mitotic spindle assembly, chromosome misalignment and embryonic lethality. Oncogene.

[154] Kesisova I.A., Nakos K.C., Tsolou A., Angelis D., Lewis J., Chatzaki A., Agianian B., Giannis A., Koffa M.D. (2013). Tripolin A, a novel small-molecule inhibitor of aurora A kinase, reveals new regulation of HURP’s distribution on microtubules. PLoS ONE.

[155] Stevens D., Gassmann R., Oegema K., Desai A. (2011). Uncoordinated loss of chromatid cohesion is a common outcome of extended metaphase arrest. PLoS ONE.

[156] Tanudji M., Shoemaker J., L’Italien L., Russell L., Chin G., Schebye X.M. (2004). Gene silencing of CENP-E by small interfering RNA in HeLa cells leads to missegregation of chromosomes after a mitotic delay. Mol. Biol. Cell.

[157] Maia A.F., Feijão T., Vromans M.J.M., Sunkel C.E., Lens S.M.A. (2010). Aurora B kinase cooperates with CENP-E to promote timely anaphase onset. Chromosoma.

[158] Wu Q., He R., Zhou H., Yu A.C.H., Zhang B., Teng J., Chen J. (2012). Cep57, a NEDD1-binding pericentriolar material component, is essential for spindle pole integrity. Cell Res..

[159] Oshimori N., Li X., Ohsugi M., Yamamoto T. (2009). Cep72 regulates the localization of key centrosomal proteins and proper bipolar spindle formation. EMBO J..

[160] Kim K., Rhee K. (2011). The pericentriolar satellite protein CEP90 is crucial for integrity of the mitotic spindle pole. J. Cell Sci..

[161] Kimura M., Yoshioka T., Saio M., Banno Y., Nagaoka H., Okano Y. (2013). Mitotic catastrophe and cell death induced by depletion of centrosomal proteins. Cell Death Dis..

[162] Mimori-Kiyosue Y., Grigoriev I., Sasaki H., Matsui C., Akhmanova A., Tsukita S., Vorobjev I. (2006). Mammalian CLASPs are required for mitotic spindle organization and kinetochore alignment. Genes Cells.

[163] Hauf S., Cole R.W., LaTerra S., Zimmer C., Schnapp G., Walter R., Heckel A., van Meel J., Rieder C.L., Peters J.-M. (2003). The small molecule Hesperadin reveals a role for Aurora B in correcting kinetochore-microtubule attachment and in maintaining the spindle assembly checkpoint. J. Cell Biol..

[164] Johnson V.L., Scott M.I.F., Holt S.V., Hussein D., Taylor S.S. (2004). Bub1 is required for kinetochore localization of BubR1, Cenp-E, Cenp-F and Mad2, and chromosome congression. J. Cell Sci..

[165] Dai J., Sultan S., Taylor S.S., Higgins J.M.G. (2005). The kinase haspin is required for mitotic histone H3 Thr 3 phosphorylation and normal metaphase chromosome alignment. Genes Dev..

[166] Dai J., Sullivan B.A., Higgins J.M.G. (2006). Regulation of mitotic chromosome cohesion by Haspin and Aurora B. Dev. Cell.

[167] Dai J., Kateneva A.V., Higgins J.M.G. (2009). Studies of haspin-depleted cells reveal that spindle-pole integrity in mitosis requires chromosome cohesion. J. Cell Sci..

[168] Fielding A.B., Dobreva I., McDonald P.C., Foster L.J., Dedhar S. (2008). Integrin-linked kinase localizes to the centrosome and regulates mitotic spindle organization. J. Cell Biol..

[169] Fang L., Seki A., Fang G. (2009). SKAP associates with kinetochores and promotes the metaphase-to-anaphase transition. Cell Cycle.

[170] Martin-Lluesma S., Stucke V.M., Nigg E.A. (2002). Role of Hec1 in spindle checkpoint signaling and kinetochore recruitment of Mad1/Mad2. Science.

[171] Joseph J., Liu S.-T., Jablonski S.A., Yen T.J., Dasso M. (2004). The RanGAP1-RanBP2 complex is essential for microtubule-kinetochore interactions in vivo. Curr. Biol..

[172] Li L., Yang L., Scudiero D.A., Miller S.A., Yu Z.X., Stukenberg P.T., Shoemaker R.H., Kotin R.M. (2007). Development of recombinant adeno-associated virus vectors carrying small interfering RNA (shHec1)-mediated depletion of kinetochore Hec1 protein in tumor cells. Gene Ther..

[173] Sundin L.J., Guimaraes G.J., Deluca J.G. (2011). The NDC80 complex proteins Nuf2 and Hec1 make distinct contributions to kinetochore-microtubule attachment in mitosis. Mol. Biol. Cell.

[174] Xu P., Virshup D.M., Lee S.H. (2014). B56-PP2A regulates motor dynamics for mitotic chromosome alignment. J. Cell Sci..

[175] DeLuca J.G., Moree B., Hickey J.M., Kilmartin J.V., Salmon E.D. (2002). hNuf2 inhibition blocks stable kinetochore-microtubule attachment and induces mitotic cell death in HeLa cells. J. Cell Biol..

[176] Haren L., Gnadt N., Wright M., Merdes A. (2009). NuMA is required for proper spindle assembly and chromosome alignment in prometaphase. BMC Res..

[177] McGuinness B.E., Hirota T., Kudo N.R., Peters J.-M., Nasmyth K. (2005). Shugoshin prevents dissociation of cohesin from centromeres during mitosis in vertebrate cells. PLoS Biol..

[178] Raaijmakers J.A., Tanenbaum M.E., Medema R.H. (2013). Systematic dissection of dynein regulators in mitosis. J. Cell Biol..

[179] Barisic M., Sohm B., Mikolcevic P., Wandke C., Rauch V., Ringer T., Hess M., Bonn G., Geley S. (2010). Spindly/CCDC99 is required for efficient chromosome congression and mitotic checkpoint regulation. Mol. Biol. Cell.

[180] Schneider L., Essmann F., Kletke A., Rio P., Hanenberg H., Wetzel W., Schulze-Osthoff K., Nurnberg B., Piekorz R.P. (2007). The transforming acidic coiled coil 3 protein is essential for spindle-dependent chromosome alignment and mitotic survival. J. Biol. Chem..

[181] Lin C.H., Hu C.K., Shih H.M. (2010). Clathrin heavy chain mediates TACC3 targeting to mitotic spindles to ensure spindle stability. J. Cell Biol..

[182] Cheeseman L.P., Harry E.F., McAinsh A.D., Prior I.A., Royle S.J. (2013). Specific removal of TACC3-ch-TOG-clathrin at metaphase deregulates kinetochore fiber tension. J. Cell Sci..

[183] Royle S.J., Bright N.A., Lagnado L. (2005). Clathrin is required for the function of the mitotic spindle. Nature.

[184] Krauss S.W., Spence J.R., Bahmanyar S., Barth A.I.M., Go M.M., Czerwinski D., Meyer A.J. (2008). Downregulation of protein 4.1R, a mature centriole protein, disrupts centrosomes, alters cell cycle progression, and perturbs mitotic spindles and anaphase. Mol. Cell. Biol..

[185] Hanisch A., Silljé H.H.W., Nigg E.A. (2006). Timely anaphase onset requires a novel spindle and kinetochore complex comprising Ska1 and Ska2. EMBO J..

[186] Sivakumar S., Daum J.R., Tipton A.R., Rankin S., Gorbsky G.J. (2014). The spindle and kinetochore-associated (Ska) complex enhances binding of the anaphase-promoting complex/cyclosome (APC/C) to chromosomes and promotes mitotic exit. Mol. Biol. Cell.

[187] Gaitanos T.N., Santamaria A., Jeyaprakash A.A., Wang B., Conti E., Nigg E.A. (2009). Stable kinetochore-microtubule interactions depend on the Ska complex and its new component Ska3/C13Orf3. EMBO J..

[188] Welburn J.P.I., Grishchuk E.L., Backer C.B., Wilson-Kubalek E.M., Yates J.R., Cheeseman I.M. (2009). The human kinetochore Ska1 complex facilitates microtubule depolymerization-coupled motility. Dev. Cell.

[189] Daum J.R., Wren J.D., Daniel J.J., Sivakumar S., McAvoy J.N., Potapova T.A., Gorbsky G.J. (2009). Ska3 is required for spindle checkpoint silencing and the maintenance of chromosome cohesion in mitosis. Curr. Biol..

[190] Raaijmakers J.A., Tanenbaum M.E., Maia A.F., Medema R.H. (2009). RAMA1 is a novel kinetochore protein involved in kinetochore-microtubule attachment. J. Cell Sci..

[191] Tokai-Nishizumi N., Ohsugi M., Suzuki E., Yamamoto T. (2005). The chromokinesin Kid is required for maintenance of proper metaphase spindle size. Mol. Biol. Cell.

[192] Huang Y., Yao Y., Xu H.-Z., Wang Z.-G., Lu L., Dai W. (2009). Defects in chromosome congression and mitotic progression in KIF18A-deficient cells are partly mediated through impaired functions of CENP-E. Cell Cycle.

[193] Liu X.-S., Zhao X.-D., Wang X., Yao Y.-X., Zhang L.-L., Shu R.-Z., Ren W.-H., Huang Y., Huang L., Gu M.-M. (2010). Germinal Cell Aplasia in Kif18a Mutant Male Mice Due to Impaired Chromosome Congression and Dysregulated BubR1 and CENP-E. Genes Cancer.

[194] Tanenbaum M.E., Macurek L., van der Vaart B., Galli M., Akhmanova A., Medema R.H. (2011). A complex of Kif18b and MCAK promotes microtubule depolymerization and is negatively regulated by Aurora kinases. Curr. Biol..

[195] Stout J.R., Yount A.L., Powers J.A., Leblanc C., Ems-McClung S.C., Walczak C.E. (2011). Kif18B interacts with EB1 and controls astral microtubule length during mitosis. Mol. Biol. Cell.

[196] Walczak C.E., Zong H., Jain S., Stout J.R. (2016). Spatial regulation of astral microtubule dynamics by Kif18B in PtK cells. Mol. Biol. Cell.

[197] Kline-Smith S.L., Khodjakov A., Hergert P., Walczak C.E. (2004). Depletion of centromeric MCAK leads to chromosome congression and segregation defects due to improper kinetochore attachments. Mol. Biol. Cell.

[198] Silljé H.H.W., Nagel S., Körner R., Nigg E.A. (2006). HURP is a Ran-importin beta-regulated protein that stabilizes kinetochore microtubules in the vicinity of chromosomes. Curr. Biol..

[199] Wong J., Fang G. (2006). HURP controls spindle dynamics to promote proper interkinetochore tension and efficient kinetochore capture. J. Cell Biol..

[200] Ye F., Tan L., Yang Q., Xia Y., Deng L.-W., Murata-Hori M., Liou Y.-C. (2011). HURP regulates chromosome congression by modulating kinesin Kif18A function. Curr. Biol..

[201] McHedlishvili N., Wieser S., Holtackers R., Mouysset J., Belwal M., Amaro A.C., Meraldi P. (2012). Kinetochores accelerate centrosome separation to ensure faithful chromosome segregation. J. Cell Sci..

[202] Raemaekers T., Ribbeck K., Beaudouin J., Annaert W., Van Camp M., Stockmans I., Smets N., Bouillon R., Ellenberg J., Carmeliet G. (2003). NuSAP, a novel microtubule-associated protein involved in mitotic spindle organization. J. Cell Biol..

[203] Li C., Xue C., Yang Q., Low B.C., Liou Y.C. (2016). NuSAP governs chromosome oscillation by facilitating the Kid-generated polar ejection force. Nat. Commun..

[204] Ma N., Matsunaga S., Morimoto A., Sakashita G., Urano T., Uchiyama S., Fukui K. (2011). The nuclear scaffold protein SAF-A is required for kinetochore-microtubule attachment and contributes to the targeting of Aurora-A to mitotic spindles. J. Cell Sci..

[205] Meraldi P., Sorger P.K. (2005). A dual role for Bub1 in the spindle checkpoint and chromosome congression. EMBO J..

[206] Ditchfield C., Johnson V.L., Tighe A., Ellston R., Haworth C., Johnson T., Mortlock A., Keen N., Taylor S.S. (2003). Aurora B couples chromosome alignment with anaphase by targeting BubR1, Mad2, and Cenp-E to kinetochores. J. Cell Biol..

[207] Xu P., Raetz E.A., Kitagawa M., Virshup D.M., Lee S.H. (2013). BUBR1 recruits PP2A via the B56 family of targeting subunits to promote chromosome congression. Biol. Open.

[208] Elowe S., Dulla K., Uldschmid A., Li X., Dou Z., Nigg E.A. (2010). Uncoupling of the spindle-checkpoint and chromosome-congression functions of BubR1. J. Cell Sci..

[209] Itoh G., Sugino S., Ikeda M., Mizuguchi M., Kanno S.-i., Amin M.A., Iemura K., Yasui A., Hirota T., Tanaka K. (2013). Nucleoporin Nup188 is required for chromosome alignment in mitosis. Cancer Sci..

[210] Holt S.V., Vergnolle M.A.S., Hussein D., Wozniak M.J., Allan V.J., Taylor S.S. (2005). Silencing Cenp-F weakens centromeric cohesion, prevents chromosome alignment and activates the spindle checkpoint. J. Cell Sci..

[211] Serio G., Margaria V., Jensen S., Oldani A., Bartek J., Bussolino F., Lanzetti L. (2011). Small GTPase Rab5 participates in chromosome congression and regulates localization of the centromere-associated protein CENP-F to kinetochores. Proc. Natl. Acad. Sci. USA.

[212] Yang Z., Guo J., Chen Q., Ding C., Du J., Zhu X. (2005). Silencing mitosin induces misaligned chromosomes, premature chromosome decondensation before anaphase onset, and mitotic cell death. Mol. Cell. Biol..

[213] De Luca M., Lavia P., Guarguaglini G. (2006). A functional interplay between Aurora-A, Plk1 and TPX2 at spindle poles: Plk1 controls centrosomal localization of Aurora-A and TPX2 spindle association. Cell Cycle.

[214] Sumara I., Giménez-Abián J.F., Gerlich D., Hirota T., Kraft C., de la Torre C., Ellenberg J., Peters J.-M. (2004). Roles of polo-like kinase 1 in the assembly of functional mitotic spindles. Curr. Biol..

[215] Neumann B., Held M., Liebel U., Erfle H., Rogers P., Pepperkok R., Ellenberg J. (2006). High-throughput RNAi screening by time-lapse imaging of live human cells. Nat. Methods.

[216] Nishino M., Kurasawa Y., Evans R., Lin S.-H., Brinkley B.R., Yu-Lee L.-Y. (2006). NudC is required for Plk1 targeting to the kinetochore and chromosome congression. Curr. Biol..

[217] Chuang C., Pan J., Hawke D.H., Lin S.H., Yu-Lee L.Y. (2013). NudC deacetylation regulates mitotic progression. PLoS ONE.

[218] Gambe A.E., Matsunaga S., Takata H., Ono-Maniwa R., Baba A., Uchiyama S., Fukui K. (2009). A nucleolar protein RRS1 contributes to chromosome congression. FEBS Lett..

[219] Ma N., Matsunaga S., Takata H., Ono-Maniwa R., Uchiyama S., Fukui K. (2007). Nucleolin functions in nucleolus formation and chromosome congression. J. Cell Sci..

[220] Zhang L., Iyer J., Chowdhury A., Ji M., Xiao L., Yang S., Chen Y., Tsai M.-Y., Dong J. (2012). KIBRA regulates aurora kinase activity and is required for precise chromosome alignment during mitosis. J. Biol. Chem..

[221] Jang C.-Y., Wong J., Coppinger J.A., Seki A., Yates J.R., Fang G. (2008). DDA3 recruits microtubule depolymerase Kif2a to spindle poles and controls spindle dynamics and mitotic chromosome movement. J. Cell Biol..

[222] Jang C.-Y., Fang G. (2011). DDA3 associates with MCAK and controls chromosome congression. Biochem. Biophys. Res. Commun..

[223] Park S.J. (2010). Huntingtin-interacting protein 1-related is required for accurate congression and segregation of chromosomes. BMB Rep..

[224] Amin M.A., Matsunaga S., Uchiyama S., Fukui K. (2008). Depletion of nucleophosmin leads to distortion of nucleolar and nuclear structures in HeLa cells. Biochem. J..

[225] Frémont S., Gérard A., Galloux M., Janvier K., Karess R.E., Berlioz-Torrent C. (2013). Beclin-1 is required for chromosome congression and proper outer kinetochore assembly. EMBO Rep..

[226] Ritchie K., Seah C., Moulin J., Isaac C., Dick F., Bérubé N.G. (2008). Loss of ATRX leads to chromosome cohesion and congression defects. J. Cell Biol..

[227] Santamaria A., Nagel S., Sillje H.H.W., Nigg E.A. (2008). The spindle protein CHICA mediates localization of the chromokinesin Kid to the mitotic spindle. Curr. Biol..

[228] Dunsch A.K., Hammond D., Lloyd J., Schermelleh L., Gruneberg U., Barr F.A. (2012). Dynein light chain 1 and a spindle-associated adaptor promote dynein asymmetry and spindle orientation. J. Cell Biol..

[229] Kukkonen-Macchi A., Sicora O., Kaczynska K., Oetken-Lindholm C., Pouwels J., Laine L., Kallio M.J. (2011). Loss of p38gamma MAPK induces pleiotropic mitotic defects and massive cell death. J. Cell Sci..

[230] Archinti M., Lacasa C., Teixidó-Travesa N., Lüders J. (2010). SPICE—A previously uncharacterized protein required for centriole duplication and mitotic chromosome congression. J. Cell Sci..

[231] Li Y., Yu W., Liang Y., Zhu X. (2007). Kinetochore dynein generates a poleward pulling force to facilitate congression and full chromosome alignment. Cell Res..

[232] Yang Z., Tulu U.S., Wadsworth P., Rieder C.L. (2007). Kinetochore dynein is required for chromosome motion and congression independent of the spindle checkpoint. Curr. Biol..

[233] Shrestha R.L., Tamura N., Fries A., Levin N., Clark J., Draviam V.M. (2014). TAO1 kinase maintains chromosomal stability by facilitating proper congression of chromosomes. Open Biol..

[234] Carleton M., Mao M., Biery M., Warrener P., Kim S., Buser C., Marshall C.G., Fernandes C., Annis J., Linsley P.S. (2006). RNA Interference-Mediated Silencing of Mitotic Kinesin KIF14 Disrupts Cell Cycle Progression and Induces Cytokinesis Failure. Mol. Cell. Biol..

[235] Prendergast L., van Vuuren C., Kaczmarczyk A., Doering V., Hellwig D., Quinn N., Hoischen C., Diekmann S., Sullivan K.F. (2011). Premitotic Assembly of Human CENPs -T and -W Switches Centromeric Chromatin to a Mitotic State. PLoS Biol..

[236] Kaczmarczyk A., Sullivan K.F. (2014). CENP-W Plays a Role in Maintaining Bipolar Spindle Structure. PLoS ONE.

[237] Chun Y., Kim R., Lee S. (2016). Centromere Protein (CENP)-W Interacts with Heterogeneous Nuclear Ribonucleoprotein (hnRNP) U and May Contribute to Kinetochore-Microtubule Attachment in Mitotic Cells. PLoS ONE.

[238] Foltz D.R., Jansen L.E., Black B.E., Bailey A.O., Yates J.R., Cleveland D.W. (2006). The human CENP-A centromeric nucleosome-associated complex. Nat. Cell Biol..

[239] McClelland S.E., Borusu S., Amaro A.C., Winter J.R., Belwal M., McAinsh A.D., Meraldi P. (2007). The CENP-A NAC/CAD kinetochore complex controls chromosome congression and spindle bipolarity. EMBO J..

[240] McAinsh A.D., Meraldi P., Draviam V.M., Toso A., Sorger P.K. (2006). The human kinetochore proteins Nnf1R and Mcm21R are required for accurate chromosome segregation. EMBO J..

[241] Bancroft J., Auckland P., Samora C.P., McAinsh A.D. (2015). Chromosome congression is promoted by CENP-Q- and CENP-E-dependent pathways. J. Cell Sci..

[242] Hua S., Wang Z., Jiang K., Huang Y., Ward T., Zhao L., Dou Z., Yao X. (2011). CENP-U Cooperates with Hec1 to Orchestrate Kinetochore-Microtubule Attachment. J. Biol. Chem..

[243] Basilico F., Maffini S., Weir J.R., Prumbaum D., Rojas A.M., Zimniak T., De Antoni A., Jeganathan S., Voss B., van Gerwen S. (2014). The pseudo GTPase CENP-M drives human kinetochore assembly. Elife.

[244] Zhu M., Wang F., Yan F., Yao P.Y., Du J., Gao X., Wang X., Wu Q., Ward T., Li J. (2008). Septin 7 Interacts with Centromere-associated Protein E and Is Required for Its Kinetochore Localization. J. Biol. Chem..

[245] Milev M.P., Hasaj B., Saint-Dic D., Snounou S., Zhao Q., Sacher M. (2015). TRAMM/TrappC12 plays a role in chromosome congression, kinetochore stability, and CENP-E recruitment. J. Cell Biol..

[246] Liu X., Zheng H., Qu C.-K. (2012). Protein tyrosine phosphatase Shp2 (Ptpn11) plays an important role in maintenance of chromosome stability. Cancer Res..

[247] Liu X., Zheng H., Li X., Wang S., Meyerson H.J., Yang W., Neel B.G., Qu C.-K. (2016). Gain-of-function mutations of Ptpn11 (Shp2) cause aberrant mitosis and increase susceptibility to DNA damage-induced malignancies. Proc. Natl. Acad. Sci. USA.

[248] Porter I.M., McClelland S.E., Khoudoli G.A., Hunter C.J., Andersen J.S., McAinsh A.D., Blow J.J., Swedlow J.R. (2007). Bod1, a novel kinetochore protein required for chromosome biorientation. J. Cell Biol..

[249] Porter I.M., Schleicher K., Porter M., Swedlow J.R. (2013). Bod1 regulates protein phosphatase 2A at mitotic kinetochores. Nat. Commun..

[250] He J., Zhang Z., Ouyang M., Yang F., Hao H., Lamb K.L., Yang J., Yin Y., Shen W.H. (2016). PTEN regulates EG5 to control spindle architecture and chromosome congression during mitosis. Nat. Commun..

[251] Park Y.Y., Nam H.-J., Do M., Lee J.-H. (2016). The p90 ribosomal S6 kinase 2 specifically affects mitotic progression by regulating the basal level, distribution and stability of mitotic spindles. Exp. Mol. Med..

[252] Vigneron S., Brioudes E., Burgess A., Labbé J.C., Lorca T., Castro A. (2010). RSK2 is a kinetochore-associated protein that participates in the spindle assembly checkpoint. Oncogene.

[253] Willard F.S., Crouch M.F. (2001). MEK, ERK, and p90RSK are present on mitotic tubulin in Swiss 3T3 cells: A role for the MAP kinase pathway in regulating mitotic exit. Cell. Signal..

[254] Hashizume C., Moyori A., Kobayashi A., Yamakoshi N., Endo A., Wong R.W. (2013). Nucleoporin Nup62 maintains centrosome homeostasis. Cell Cycle.

[255] Wu Z., Jin Z., Zhang X., Shen N., Wang J., Zhao Y., Mei L. (2016). Nup62, associated with spindle microtubule rather than spindle matrix, is involved in chromosome alignment and spindle assembly during mitosis. Cell Biol. Int..

[256] Kwon H.J., Park J.E., Song H., Jang C.-Y. (2016). DDA3 and Mdp3 modulate Kif2a recruitment onto the mitotic spindle to control minus-end spindle dynamics. J. Cell Sci..

[257] Kim S., Jang C.-Y. (2016). ANKRD53 interacts with DDA3 and regulates chromosome integrity during mitosis. Biochem. Biophys. Res. Commun..

[258] Koliou X., Fedonidis C., Kalpachidou T., Mangoura D. (2016). Nuclear import mechanism of neurofibromin for localization on the spindle and function in chromosome congression. J. Neurochem..

[259] O’Regan L., Sampson J., Richards M.W., Knebel A., Roth D., Hood F.E., Straube A., Royle S.J., Bayliss R., Fry A.M. (2015). Hsp72 is targeted to the mitotic spindle by Nek6 to promote K-fiber assembly and mitotic progression. J. Cell Biol..

[260] de Souza E.E., Hehnly H., Perez A.M., Meirelles G.V., Smetana J.H.C., Doxsey S., Kobarg J. (2015). Human Nek7-interactor RGS2 is required for mitotic spindle organization. Cell Cycle.

[261] Foley E.A., Maldonado M., Kapoor T.M. (2011). Formation of stable attachments between kinetochores and microtubules depends on the B56-PP2A phosphatase. Nat. Cell Biol..

[262] Kitajima T.S., Sakuno T., Ishiguro K.-i., Iemura S.-i., Natsume T., Kawashima S.A., Watanabe Y. (2006). Shugoshin collaborates with protein phosphatase 2A to protect cohesin. Nature.

[263] Jaramillo-Lambert A., Hao J., Xiao H., Li Y., Han Z., Zhu W. (2013). Acidic nucleoplasmic DNA-binding protein (And-1) controls chromosome congression by regulating the assembly of centromere protein A (CENP-A) at centromeres. J. Biol. Chem..

[264] Lee M.H., Lin L., Equilibrina I., Uchiyama S., Matsunaga S., Fukui K. (2011). ASURA (PHB2) Is Required for Kinetochore Assembly and Subsequent Chromosome Congression. Acta Histochem. Cytochem..

[265] Oh H.J., Kim M.J., Song S.J., Kim T., Lee D., Kwon S.-H., Choi E.-J., Lim D.-S. (2010). MST1 limits the kinase activity of aurora B to promote stable kinetochore-microtubule attachment. Curr. Biol..

[266] Shimizu H., Nagamori I., Yabuta N., Nojima H. (2009). GAK, a regulator of clathrin-mediated membrane traffic, also controls centrosome integrity and chromosome congression. J. Cell Sci..

[267] Zhuo X., Guo X., Zhang X., Jing G., Wang Y., Chen Q., Jiang Q., Liu J., Zhang C. (2015). Usp16 regulates kinetochore localization of Plk1 to promote proper chromosome alignment in mitosis. J. Cell Biol..

[268] Pfarr C.M., Coue M., Grissom P.M., Hays T.S., Porter M.E., McIntosh J.R. (1990). Cytoplasmic dynein is localized to kinetochores during mitosis. Nature.

[269] Steuer E.R., Wordeman L., Schroer T.A., Sheetz M.P. (1990). Localization of cytoplasmic dynein to mitotic spindles and kinetochores. Nature.

[270] Maiato H., Lince-Faria M. (2010). The perpetual movements of anaphase. Cell Mol. Life Sci..

[271] King J.M., Hays T.S., Nicklas R.B. (2000). Dynein is a transient kinetochore component whose binding is regulated by microtubule attachment, not tension. J. Cell Biol..

[272] Wojcik E., Basto R., Serr M., Scaerou F., Karess R., Hays T. (2001). Kinetochore dynein: Its dynamics and role in the transport of the Rough deal checkpoint protein. Nat. Cell Biol..

[273] Lombillo V.A., Nislow C., Yen T.J., Gelfand V.I., McIntosh J.R. (1995). Antibodies to the kinesin motor domain and CENP-E inhibit microtubule depolymerization-dependent motion of chromosomes in vitro. J. Cell Biol..

[274] Lombillo V.A., Stewart R.J., McIntosh J.R. (1995). Minus-end-directed motion of kinesin-coated microspheres driven by microtubule depolymerization. Nature.

[275] Schneider B. (1933). Über die umordunug der chromosomen bei der mitose. Z. Zellf Mikr Anat..

[276] Bajer A. (1954). Cine-micrographic studies on mitosis in endosperm I. Acta Soc. Bot. Poloniae.

[277] Bajer A., Molè-Bajer J. (1956). Cine-micrographic studies on mitosis in endosperm. II. Chromosoma.

[278] Uretz R.B., Bloom W., Zirkle R.E. (1954). Irradiation of parts of individual cells. II. Effects of an ultraviolet microbeam focused on parts of chromosomes. Science.

[279] Bloom W., Zirkle R.E., Uretz R.B. (1955). Irradiation of parts of individual cells. III. Effects of chromosomal and extrachromosomal irradiation on chromosome movements. Ann. N. Y. Acad. Sci..

[280] Zirkle R.E. (1957). Partial-cell irradiation. Adv. Biol. Med. Phys..

[281] Rickards G.K. (1975). Prophase chromosome movements in living house cricket spermatocytes and their relationship to prometaphase, anaphase and granule movements. Chromosoma.

[282] Roos U.P. (1976). Light and electron microscopy of rat kangaroo cells in mitosis. III. Patterns of chromosome behavior during prometaphase. Chromosoma.

[283] Rieder C.L., Alexander S.P. (1990). Kinetochores are transported poleward along a single astral microtubule during chromosome attachment to the spindle in newt lung cells. J. Cell Biol..

[284] Merdes A., De Mey J. (1990). The mechanism of kinetochore-spindle attachment and polewards movement analyzed in PtK2 cells at the prophase-prometaphase transition. Eur. J. Cell Biol..

[285] Wordeman L., Steuer E.R., Sheetz M.P., Mitchison T. (1991). Chemical subdomains within the kinetochore domain of isolated CHO mitotic chromosomes. J. Cell Biol..

[286] Vorozhko V.V., Emanuele M.J., Kallio M.J., Stukenberg P.T., Gorbsky G.J. (2008). Multiple mechanisms of chromosome movement in vertebrate cells mediated through the Ndc80 complex and dynein/dynactin. Chromosoma.

[287] Mitchison T.J., Kirschner M.W. (1985). Properties of the kinetochore in vitro. II. Microtubule capture and ATP-dependent translocation. J. Cell Biol..

[288] Hyman A.A., Mitchison T.J. (1991). Two different microtubule-based motor activities with opposite polarities in kinetochores. Nature.

[289] Yen T.J., Compton D.A., Wise D., Zinkowski R.P., Brinkley B.R., Earnshaw W.C., Cleveland D.W. (1991). CENP-E, a novel human centromere-associated protein required for progression from metaphase to anaphase. EMBO J..

[290] Yen T.J., Li G., Schaar B.T., Szilak I., Cleveland D.W. (1992). CENP-E is a putative kinetochore motor that accumulates just before mitosis. Nature.

[291] Yao X., Anderson K.L., Cleveland D.W. (1997). The microtubule-dependent motor centromere-associated protein E (CENP-E) is an integral component of kinetochore corona fibers that link centromeres to spindle microtubules. J. Cell Biol..

[292] Cooke C.A., Schaar B., Yen T.J., Earnshaw W.C. (1997). Localization of CENP-E in the fibrous corona and outer plate of mammalian kinetochores from prometaphase through anaphase. Chromosoma.

[293] Wood K.W., Sakowicz R., Goldstein L.S., Cleveland D.W. (1997). CENP-E is a plus end-directed kinetochore motor required for metaphase chromosome alignment. Cell.

[294] Yao X., Abrieu A., Zheng Y., Sullivan K.F., Cleveland D.W. (2000). CENP-E forms a link between attachment of spindle microtubules to kinetochores and the mitotic checkpoint. Nat. Cell Biol..

[295] Schaar B.T., Chan G.K., Maddox P., Salmon E.D., Yen T.J. (1997). CENP-E function at kinetochores is essential for chromosome alignment. J. Cell Biol..

[296] Yucel J.K., Marszalek J.D., McIntosh J.R., Goldstein L.S., Cleveland D.W., Philp A.V. (2000). CENP-meta, an essential kinetochore kinesin required for the maintenance of metaphase chromosome alignment in Drosophila. J. Cell Biol..

[297] McEwen B.F., Chan G.K., Zubrowski B., Savoian M.S., Sauer M.T., Yen T.J. (2001). CENP-E is essential for reliable bioriented spindle attachment, but chromosome alignment can be achieved via redundant mechanisms in mammalian cells. Mol. Biol. Cell.

[298] Putkey F.R., Cramer T., Morphew M.K., Silk A.D., Johnson R.S., McIntosh J.R., Cleveland D.W. (2002). Unstable kinetochore-microtubule capture and chromosomal instability following deletion of CENP-E. Dev. Cell.

[299] Cleveland D.W., Mao Y., Sullivan K.F. (2003). Centromeres and kinetochores: from epigenetics to mitotic checkpoint signaling. Cell.

[300] Kapoor T.M., Lampson M.A., Hergert P., Cameron L., Cimini D., Salmon E.D., McEwen B.F., Khodjakov A. (2006). Chromosomes can congress to the metaphase plate before biorientation. Science.

[301] Espeut J., Gaussen A., Bieling P., Morin V., Prieto S., Fesquet D., Surrey T., Abrieu A. (2008). Phosphorylation relieves autoinhibition of the kinetochore motor Cenp-E. Mol. Cell.

[302] Kim Y., Heuser J.E., Waterman C.M., Cleveland D.W. (2008). CENP-E combines a slow, processive motor and a flexible coiled coil to produce an essential motile kinetochore tether. J. Cell Biol..

[303] Gudimchuk N., Vitre B., Kim Y., Kiyatkin A., Cleveland D.W., Ataullakhanov F.I., Grishchuk E.L. (2013). Kinetochore kinesin CENP-E is a processive bi-directional tracker of dynamic microtubule tips. Nat. Cell Biol..

[304] Vitre B., Gudimchuk N., Borda R., Kim Y., Heuser J.E., Cleveland D.W., Grishchuk E.L. (2014). Kinetochore-microtubule attachment throughout mitosis potentiated by the elongated stalk of the kinetochore kinesin CENP-E. Mol. Biol. Cell.

[305] Cai S., O’Connell C.B., Khodjakov A., Walczak C.E. (2009). Chromosome congression in the absence of kinetochore fibres. Nat. Cell Biol..

[306] Roos U.P. (1973). Light and electron microscopy of rat kangaroo cells in mitosis. II. Kinetochore structure and function. Chromosoma.

[307] Magidson V., Paul R., Yang N., Ault J.G., O’Connell C.B., Tikhonenko I., McEwen B.F., Mogilner A., Khodjakov A. (2015). Adaptive changes in the kinetochore architecture facilitate proper spindle assembly. Nat. Cell Biol..

[308] Maddox P.S., Oegema K., Desai A., Cheeseman I.M. (2004). Holoer than thou: Chromosome segregation and kinetochore function in *C. elegans*. Chromosome Res..

[309] Powers J., Rose D.J., Saunders A., Dunkelbarger S., Strome S., Saxton W.M. (2004). Loss of KLP-19 polar ejection force causes misorientation and missegregation of holocentric chromosomes. J. Cell Biol..

[310] Rieder C.L., Cole R.W., Khodjakov A., Sluder G. (1995). The checkpoint delaying anaphase in response to chromosome monoorientation is mediated by an inhibitory signal produced by unattached kinetochores. J. Cell Biol..

[311] Brenner S.L., Liaw L.H., Berns M.W. (1980). Laser microirradiation of kinetochores in mitotic PtK2 cells: Chromatid separation and micronucleus formation. Cell Biophys..

[312] Brinkley B.R., Zinkowski R.P., Mollon W.L., Davis F.M., Pisegna M.A., Pershouse M., Rao P.N. (1988). Movement and segregation of kinetochores experimentally detached from mammalian chromosomes. Nature.

[313] Wise D.A., Brinkley B.R. (1997). Mitosis in cells with unreplicated genomes (MUGs): Spindle assembly and behavior of centromere fragments. Cell Motil Cytoskeleton.

[314] O’Connell C.B., Loncarek J., Hergert P., Kourtidis A., Conklin D.S., Khodjakov A. (2008). The spindle assembly checkpoint is satisfied in the absence of interkinetochore tension during mitosis with unreplicated genomes. J. Cell Biol..

[315] Khodjakov A., Cole R.W., McEwen B.F., Buttle K.F., Rieder C.L. (1997). Chromosome fragments possessing only one kinetochore can congress to the spindle equator. J. Cell Biol..

[316] Barisic M., Maiato H. (2015). Dynein prevents erroneous kinetochore-microtubule attachments in mitosis. Cell Cycle.

[317] Iemura K., Tanaka K. (2015). Chromokinesin Kid and kinetochore kinesin CENP-E differentially support chromosome congression without end-on attachment to microtubules. Nat. Commun..

[318] Nousiainen M., Silljé H.H.W., Sauer G., Nigg E.A., Körner R. (2006). Phosphoproteome analysis of the human mitotic spindle. Proc. Natl. Acad. Sci. USA.

[319] Liao H., Li G., Yen T.J. (1994). Mitotic regulation of microtubule cross-linking activity of CENP-E kinetochore protein. Science.

[320] Zecevic M., Catling A.D., Eblen S.T., Renzi L., Hittle J.C., Yen T.J., Gorbsky G.J., Weber M.J. (1998). Active MAP kinase in mitosis: localization at kinetochores and association with the motor protein CENP-E. J. Cell Biol..

[321] Kim Y., Holland A.J., Lan W., Cleveland D.W. (2010). Aurora kinases and protein phosphatase 1 mediate chromosome congression through regulation of CENP-E. Cell.

[322] Whyte J., Bader J.R., Tauhata S.B., Raycroft M., Hornick J., Pfister K.K., Lane W.S., Chan G.K., Hinchcliffe E.H., Vaughan P.S. (2008). Phosphorylation regulates targeting of cytoplasmic dynein to kinetochores during mitosis. J. Cell Biol..

[323] Bader J.R., Kasuboski J.M., Winding M., Vaughan P.S., Hinchcliffe E.H., Vaughan K.T. (2011). Polo-like kinase1 is required for recruitment of dynein to kinetochores during mitosis. J Biol. Chem..

[324] Kardon J.R., Reck-Peterson S.L., Vale R.D. (2009). Regulation of the processivity and intracellular localization of Saccharomyces cerevisiae dynein by dynactin. Proc. Natl. Acad. Sci. USA.

[325] McKenney R.J., Huynh W., Tanenbaum M.E., Bhabha G., Vale R.D. (2014). Activation of cytoplasmic dynein motility by dynactin-cargo adapter complexes. Science.

[326] Zhang X.-D., Goeres J., Zhang H., Yen T.J., Porter A.C.G., Matunis M.J. (2008). SUMO-2/3 modification and binding regulate the association of CENP-E with kinetochores and progression through mitosis. Mol. Cell.

[327] Ashar H.R., James L., Gray K., Carr D., Black S., Armstrong L., Bishop W.R., Kirschmeier P. (2000). Farnesyl transferase inhibitors block the farnesylation of CENP-E and CENP-F and alter the association of CENP-E with the microtubules. J. Biol. Chem..

[328] Schafer-Hales K., Iaconelli J., Snyder J.P., Prussia A., Nettles J.H., El-Naggar A., Khuri F.R., Giannakakou P., Marcus A.I. (2007). Farnesyl transferase inhibitors impair chromosomal maintenance in cell lines and human tumors by compromising CENP-E and CENP-F function. Mol. Cancer Ther..

[329] Crespo N.C., Ohkanda J., Yen T.J., Hamilton A.D., Sebti S.M. (2001). The farnesyltransferase inhibitor, FTI-2153, blocks bipolar spindle formation and chromosome alignment and causes prometaphase accumulation during mitosis of human lung cancer cells. J. Biol. Chem..

[330] Crespo N.C., Delarue F., Ohkanda J., Carrico D., Hamilton A.D., Sebti S.M. (2002). The farnesyltransferase inhibitor, FTI-2153, inhibits bipolar spindle formation during mitosis independently of transformation and Ras and p53 mutation status. Cell Death Differ..

[331] Hussein D., Taylor S.S. (2002). Farnesylation of Cenp-F is required for G2/M progression and degradation after mitosis. J. Cell Sci..

[332] Brown K.D., Coulson R.M., Yen T.J., Cleveland D.W. (1994). Cyclin-like accumulation and loss of the putative kinetochore motor CENP-E results from coupling continuous synthesis with specific degradation at the end of mitosis. J. Cell Biol..

[333] Gurden M.D.J., Holland A.J., van Zon W., Tighe A., Vergnolle M.A., Andres D.A., Spielmann H.P., Malumbres M., Wolthuis R.M.F., Cleveland D.W. (2010). Cdc20 is required for the post-anaphase, KEN-dependent degradation of centromere protein F. J. Cell Sci..

[334] Holland A.J., Reis R.M., Niessen S., Pereira C., Andres D.A., Spielmann H.P., Cleveland D.W., Desai A., Gassmann R. (2015). Preventing farnesylation of the dynein adaptor Spindly contributes to the mitotic defects caused by farnesyltransferase inhibitors. Mol. Biol. Cell.

[335] Moudgil D.K., Westcott N., Famulski J.K., Patel K., Macdonald D., Hang H., Chan G.K.T. (2015). A novel role of farnesylation in targeting a mitotic checkpoint protein, human Spindly, to kinetochores. J. Cell Biol..

[336] Hewitt L., Tighe A., Santaguida S., White A.M., Jones C.D., Musacchio A., Green S., Taylor S.S. (2010). Sustained Mps1 activity is required in mitosis to recruit O-Mad2 to the Mad1-C-Mad2 core complex. J. Cell Biol..

[337] Jelluma N., Brenkman A.B., van den Broek N.J.F., Cruijsen C.W.A., van Osch M.H.J., Lens S.M.A., Medema R.H., Kops G.J.P.L. (2008). Mps1 phosphorylates Borealin to control Aurora B activity and chromosome alignment. Cell.

[338] Maure J.-F., Kitamura E., Tanaka T.U. (2007). Mps1 kinase promotes sister-kinetochore bi-orientation by a tension-dependent mechanism. Curr. Biol..

[339] Wang X., Yu H., Xu L., Zhu T., Zheng F., Fu C., Wang Z., Dou Z. (2014). Dynamic autophosphorylation of mps1 kinase is required for faithful mitotic progression. PLoS ONE.

[340] Maciejowski J., George K.A., Terret M.-E., Zhang C., Shokat K.M., Jallepalli P.V. (2010). Mps1 directs the assembly of Cdc20 inhibitory complexes during interphase and mitosis to control M phase timing and spindle checkpoint signaling. J. Cell Biol..

[341] Santaguida S., Tighe A., D’Alise A.M., Taylor S.S., Musacchio A. (2010). Dissecting the role of MPS1 in chromosome biorientation and the spindle checkpoint through the small molecule inhibitor reversine. J. Cell Biol..

[342] Abrieu A., Magnaghi-Jaulin L., Kahana J.A., Peter M., Castro A., Vigneron S., Lorca T., Cleveland D.W., Labbe J.C. (2001). Mps1 is a kinetochore-associated kinase essential for the vertebrate mitotic checkpoint. Cell.

[343] Verhey K.J., Gaertig J. (2007). The tubulin code. Cell Cycle.

[344] Janke C. (2014). The tubulin code: Molecular components, readout mechanisms, and functions. J. Cell Biol..

[345] Barisic M., Silva e Sousa R., Tripathy S.K., Magiera M.M., Zaytsev A.V., Pereira A.L., Janke C., Grishchuk E.L., Maiato H. (2015). Mitosis. Microtubule detyrosination guides chromosomes during mitosis. Science.

[346] Barisic M., Maiato H. (2016). The Tubulin Code: A Navigation System for Chromosomes during Mitosis. Trends Cell Biol..

[347] Park I.Y., Powell R.T., Tripathi D.N., Dere R., Ho T.H., Blasius T.L., Chiang Y.C., Davis I.J., Fahey C.C., Hacker K.E. (2016). Dual Chromatin and Cytoskeletal Remodeling by SETD2. Cell.

[348] Hammond J.W., Huang C.F., Kaech S., Jacobson C., Banker G., Verhey K.J. (2010). Posttranslational modifications of tubulin and the polarized transport of kinesin-1 in neurons. Mol. Biol. Cell.

[349] Konishi Y., Setou M. (2009). Tubulin tyrosination navigates the kinesin-1 motor domain to axons. Nat. Neurosci..

[350] Maas C., Belgardt D., Lee H.K., Heisler F.F., Lappe-Siefke C., Magiera M.M., van Dijk J., Hausrat T.J., Janke C., Kneussel M. (2009). Synaptic activation modifies microtubules underlying transport of postsynaptic cargo. Proc. Natl. Acad. Sci. USA.

[351] Reed N.A., Cai D., Blasius T.L., Jih G.T., Meyhofer E., Gaertig J., Verhey K.J. (2006). Microtubule acetylation promotes kinesin-1 binding and transport. Curr. Biol..

[352] Kaul N., Soppina V., Verhey K.J. (2014). Effects of alpha-tubulin K40 acetylation and detyrosination on kinesin-1 motility in a purified system. Biophys. J..

[353] Sirajuddin M., Rice L.M., Vale R.D. (2014). Regulation of microtubule motors by tubulin isotypes and post-translational modifications. Nat. Cell Biol..

[354] Bobinnec Y., Moudjou M., Fouquet J.P., Desbruyeres E., Edde B., Bornens M. (1998). Glutamylation of centriole and cytoplasmic tubulin in proliferating non-neuronal cells. Cell Motil. Cytoskeleton.

[355] Gundersen G.G., Bulinski J.C. (1986). Distribution of tyrosinated and nontyrosinated α-tubulin during mitosis. J. Cell Biol..

[356] Gundersen G.G., Kalnoski M.H., Bulinski J.C. (1984). Distinct populations of microtubules: Tyrosinated and nontyrosinated alpha tubulin are distributed differently in vivo. Cell.

[357] Wilson P.J., Forer A. (1997). Effects of nanomolar taxol on crane-fly spermatocyte spindles indicate that acetylation of kinetochore microtubules can be used as a marker of poleward tubulin flux. Cell Motil. Cytoskeleton.

[358] McKenney R.J., Huynh W., Vale R.D., Sirajuddin M. (2016). Tyrosination of alpha-tubulin controls the initiation of processive dynein-dynactin motility. EMBO J..

[359] Nirschl J.J., Magiera M.M., Lazarus J.E., Janke C., Holzbaur E.L. (2016). alpha-Tubulin Tyrosination and CLIP-170 Phosphorylation Regulate the Initiation of Dynein-Driven Transport in Neurons. Cell Rep..

[360] Peris L., Thery M., Faure J., Saoudi Y., Lafanechere L., Chilton J.K., Gordon-Weeks P., Galjart N., Bornens M., Wordeman L. (2006). Tubulin tyrosination is a major factor affecting the recruitment of CAP-Gly proteins at microtubule plus ends. J. Cell Biol..

[361] Kubo T., Yanagisawa H.A., Yagi T., Hirono M., Kamiya R. (2010). Tubulin polyglutamylation regulates axonemal motility by modulating activities of inner-arm dyneins. Curr. Biol..

[362] Wang Z., Sheetz M.P. (2000). The C-terminus of tubulin increases cytoplasmic dynein and kinesin processivity. Biophys. J..

[363] Alper J.D., Decker F., Agana B., Howard J. (2014). The motility of axonemal dynein is regulated by the tubulin code. Biophys. J..

[364] Sardar H.S., Gilbert S.P. (2012). Microtubule capture by mitotic kinesin centromere protein E (CENP-E). J. Biol. Chem..

[365] Belyy V., Schlager M.A., Foster H., Reimer A.E., Carter A.P., Yildiz A. (2016). The mammalian dynein-dynactin complex is a strong opponent to kinesin in a tug-of-war competition. Nat. Cell Biol..

[366] Iniguez A., Allard J. (2016). Spatial pattern formation in microtubule post-translational modifications and the tight localization of motor-driven cargo. J. Math. Biol..

[367] Skiniotis G., Cochran J.C., Muller J., Mandelkow E., Gilbert S.P., Hoenger A. (2004). Modulation of kinesin binding by the C-termini of tubulin. EMBO J..

[368] Neumann E., Garcia-Saez I., DeBonis S., Wade R.H., Kozielski F., Conway J.F. (2006). Human kinetochore-associated kinesin CENP-E visualized at 17 A resolution bound to microtubules. J. Mol. Biol..

[369] Garcia-Saez I., Yen T., Wade R.H., Kozielski F. (2004). Crystal structure of the motor domain of the human kinetochore protein CENP-E. J. Mol. Biol..

[370] Musinipally V., Howes S., Alushin G.M., Nogales E. (2013). The microtubule binding properties of CENP-E’s C-terminus and CENP-F. J. Mol. Biol..

[371] Wang Q., Crevenna A.H., Kunze I., Mizuno N. (2014). Structural basis for the extended CAP-Gly domains of p150(glued) binding to microtubules and the implication for tubulin dynamics. Proc. Natl. Acad. Sci. USA.

[372] Yan S., Guo C., Hou G., Zhang H., Lu X., Williams J.C., Polenova T. (2015). Atomic-resolution structure of the CAP-Gly domain of dynactin on polymeric microtubules determined by magic angle spinning NMR spectroscopy. Proc. Natl. Acad. Sci. USA.

[373] Pereira A.J., Matos I., Lince-Faria M., Maiato H. (2009). Dissecting mitosis with laser microsurgery and RNAi in Drosophila cells. Methods Mol. Biol..

[374] McIntosh J.R., Hepler P.K., Van Wie D.G. (1969). Model for mitosis. Nature.

[375] Goode D. (1981). Microtubule turnover as a mechanism of mitosis and its possible evolution. Biosystems.

[376] Margolis R.L., Wilson L., Keifer B.I. (1978). Mitotic mechanism based on intrinsic microtubule behaviour. Nature.

[377] Nicklas R.B., Kubai D.F., Hays T.S. (1982). Spindle microtubules and their mechanical associations after micromanipulation in anaphase. J. Cell Biol..

[378] Mastronarde D.N., McDonald K.L., Ding R., McIntosh J.R. (1993). Interpolar spindle microtubules in PTK cells. J. Cell Biol..

[379] Shimamoto Y., Maeda Y.T., Ishiwata S., Libchaber A.J., Kapoor T.M. (2011). Insights into the micromechanical properties of the metaphase spindle. Cell.

[380] Vladimirou E., McHedlishvili N., Gasic I., Armond J.W., Samora C.P., Meraldi P., McAinsh A.D. (2013). Nonautonomous Movement of Chromosomes in Mitosis. Dev. Cell.

[381] Pereira A.J., Maiato H. (2012). Maturation of the kinetochore-microtubule interface and the meaning of metaphase. Chromosome Res..

[382] Cross R.A., McAinsh A. (2014). Prime movers: The mechanochemistry of mitotic kinesins. Nat. Rev. Mol. Cell Biol..

[383] Royle S.J. (2012). The role of clathrin in mitotic spindle organisation. J. Cell Sci..

[384] Maiato H., Sampaio P., Sunkel C.E. (2004). Microtubule-associated proteins and their essential roles during mitosis. Int. Rev. Cytol..

[385] Hoffman D.B., Pearson C.G., Yen T.J., Howell B.J., Salmon E.D. (2001). Microtubule-dependent changes in assembly of microtubule motor proteins and mitotic spindle checkpoint proteins at PtK1 kinetochores. Mol. Biol. Cell.

[386] Ohashi A., Ohori M., Iwai K., Nambu T., Miyamoto M., Kawamoto T., Okaniwa M. (2015). A Novel Time-Dependent CENP-E Inhibitor with Potent Antitumor Activity. PLoS ONE.

[387] Gorbsky G.J. (2013). Cohesion fatigue. Curr. Biol..

[388] Daum J.R., Potapova T.A., Sivakumar S., Daniel J.J., Flynn J.N., Rankin S., Gorbsky G.J. (2011). Cohesion fatigue induces chromatid separation in cells delayed at metaphase. Curr. Biol..

[389] Bannigan A., Lizotte-Waniewski M., Riley M., Baskin T.I. (2008). Emerging molecular mechanisms that power and regulate the anastral mitotic spindle of flowering plants. Cell Motil. Cytoskelet..

[390] Yamada M., Goshima G. (2017). Mitotic spindle assembly in land plants: Molecules and mechanisms. Biology.

[391] Khodjakov A., Cole R.W., Bajer A.S., Rieder C.L. (1996). The force for poleward chromosome motion in Haemanthus cells acts along the length of the chromosome during metaphase but only at the kinetochore during anaphase. J. Cell Biol..

[392] Schuh M., Ellenberg J. (2007). Self-organization of MTOCs replaces centrosome function during acentrosomal spindle assembly in live mouse oocytes. Cell.

[393] Dumont J., Desai A. (2012). Acentrosomal spindle assembly and chromosome segregation during oocyte meiosis. Trends Cell Biol..

[394] Bennabi I., Terret M.E., Verlhac M.H. (2016). Meiotic spindle assembly and chromosome segregation in oocytes. J. Cell Biol..

[395] Kitajima T.S., Ohsugi M., Ellenberg J. (2011). Complete kinetochore tracking reveals error-prone homologous chromosome biorientation in mammalian oocytes. Cell.

[396] Ohsugi M., Adachi K., Horai R., Kakuta S., Sudo K., Kotaki H., Tokai-Nishizumi N., Sagara H., Iwakura Y., Yamamoto T. (2008). Kid-mediated chromosome compaction ensures proper nuclear envelope formation. Cell.

[397] Gui L., Homer H. (2012). Spindle assembly checkpoint signalling is uncoupled from chromosomal position in mouse oocytes. Development.

[398] Radford S.J., Hoang T.L., Gluszek A.A., Ohkura H., McKim K.S. (2015). Lateral and End-On Kinetochore Attachments Are Coordinated to Achieve Bi-orientation in Drosophila Oocytes. PLoS Genet.

[399] Wignall S.M., Villeneuve A.M. (2009). Lateral microtubule bundles promote chromosome alignment during acentrosomal oocyte meiosis. Nat. Cell Biol..

[400] Dumont J., Oegema K., Desai A. (2010). A kinetochore-independent mechanism drives anaphase chromosome separation during acentrosomal meiosis. Nat. Cell Biol..

[401] Wickstead B., Gull K. (2007). Dyneins across eukaryotes: A comparative genomic analysis. Traffic.

[402] ten Hoopen R., Schleker T., Manteuffel R., Schubert I. (2002). Transient CENP-E-like kinetochore proteins in plants. Chromosome Res..

[403] Naito H., Goshima G. (2015). NACK kinesin is required for metaphase chromosome alignment and cytokinesis in the moss Physcomitrella patens. Cell Struct. Funct..

[404] Moutinho-Pereira S., Stuurman N., Afonso O., Hornsveld M., Aguiar P., Goshima G., Vale R.D., Maiato H. (2013). Genes involved in centrosome-independent mitotic spindle assembly in Drosophila S2 cells. Proc. Natl. Acad. Sci. USA.

[405] Thompson S.L., Bakhoum S.F., Compton D.A. (2010). Mechanisms of chromosomal instability. Curr. Biol..

[406] Carter S.L., Eklund A.C., Kohane I.S., Harris L.N., Szallasi Z. (2006). A signature of chromosomal instability inferred from gene expression profiles predicts clinical outcome in multiple human cancers. Nat. Genet..

[407] Choi C.-M., Seo K.W., Jang S.J., Oh Y.-M., Shim T.-S., Kim W.S., Lee D.-S., Lee S.-D. (2009). Chromosomal instability is a risk factor for poor prognosis of adenocarcinoma of the lung: Fluorescence in situ hybridization analysis of paraffin-embedded tissue from Korean patients. Lung Cancer.

[408] Lee A.J.X., Endesfelder D., Rowan A.J., Walther A., Birkbak N.J., Futreal P.A., Downward J., Szallasi Z., Tomlinson I.P.M., Howell M. (2011). Chromosomal instability confers intrinsic multidrug resistance. Cancer Res..

[409] McClelland S.E., Burrell R.A., Swanton C. (2009). Chromosomal instability: A composite phenotype that influences sensitivity to chemotherapy. Cell Cycle.

[410] Swanton C., Nicke B., Schuett M., Eklund A.C., Ng C., Li Q., Hardcastle T., Lee A., Roy R., East P. (2009). Chromosomal instability determines taxane response. Proc. Natl. Acad. Sci. USA.

[411] Birkbak N.J., Eklund A.C., Li Q., McClelland S.E., Endesfelder D., Tan P., Tan I.B., Richardson A.L., Szallasi Z., Swanton C. (2011). Paradoxical relationship between chromosomal instability and survival outcome in cancer. Cancer Res..

[412] Burrell R.A., Juul N., Johnston S.R., Reis-Filho J.S., Szallasi Z., Swanton C. (2010). Targeting chromosomal instability and tumour heterogeneity in HER2-positive breast cancer. J. Cell. Biochem..

[413] Roschke A.V., Kirsch I.R. (2005). Targeting cancer cells by exploiting karyotypic complexity and chromosomal instability. Cell Cycle.

[414] Gordon D.J., Resio B., Pellman D. (2012). Causes and consequences of aneuploidy in cancer. Nat. Rev. Genet..

[415] Maiato H., Logarinho E. (2014). Mitotic spindle multipolarity without centrosome amplification. Nat. Cell Biol..

[416] Weaver B.A.A., Bonday Z.Q., Putkey F.R., Kops G.J.P.L., Silk A.D., Cleveland D.W. (2003). Centromere-associated protein-E is essential for the mammalian mitotic checkpoint to prevent aneuploidy due to single chromosome loss. J. Cell Biol..

[417] Silk A.D., Zasadil L.M., Holland A.J., Vitre B., Cleveland D.W., Weaver B.A. (2013). Chromosome missegregation rate predicts whether aneuploidy will promote or suppress tumors. Proc. Natl. Acad. Sci. USA.

[418] Weaver B.A.A., Silk A.D., Montagna C., Verdier-Pinard P., Cleveland D.W. (2007). Aneuploidy acts both oncogenically and as a tumor suppressor. Cancer Cell.

[419] Zasadil L.M., Britigan E.M.C., Ryan S.D., Kaur C., Guckenberger D.J., Beebe D.J., Moser A.R., Weaver B.A. (2016). High rates of chromosome missegregation suppress tumor progression but do not inhibit tumor initiation. Mol. Biol. Cell.

[420] Morais da Silva S., Moutinho-Santos T., Sunkel C.E. (2013). A tumor suppressor role of the Bub3 spindle checkpoint protein after apoptosis inhibition. J. Cell Biol..

[421] Clemente-Ruiz M., Muzzopappa M., Milán M. (2014). Tumor suppressor roles of CENP-E and Nsl1 in Drosophila epithelial tissues. Cell Cycle.

[422] Kullmann F., Judex M., Ballhorn W., Jüsten H.P., Wessinghage D., Welsh J., Yen T.J., Lang B., Hittle J.C., McClelland M. (1999). Kinesin-like protein CENP-E is upregulated in rheumatoid synovial fibroblasts. Arthritis Res..

[423] Kung P.-P., Martinez R., Zhu Z., Zager M., Blasina A., Rymer I., Hallin J., Xu M., Carroll C., Chionis J. (2014). Chemogenetic evaluation of the mitotic kinesin CENP-E reveals a critical role in triple-negative breast cancer. Mol. Cancer Ther..

[424] Liu Z., Ling K., Wu X., Cao J., Liu B., Li S., Si Q., Cai Y., Yan C., Zhang Y. (2009). Reduced expression of cenp-e in human hepatocellular carcinoma. J. Exp. Clin. Cancer Res..

[425] Kumar A., Purohit R. (2012). Computational screening and molecular dynamics simulation of disease associated nsSNPs in CENP-E. Mutat. Res..

[426] Mirzaa G.M., Vitre B., Carpenter G., Abramowicz I., Gleeson J.G., Paciorkowski A.R., Cleveland D.W., Dobyns W.B., O’Driscoll M. (2014). Mutations in CENPE define a novel kinetochore-centromeric mechanism for microcephalic primordial dwarfism. Hum. Genet..

[427] Nagahara M., Nishida N., Iwatsuki M., Ishimaru S., Mimori K., Tanaka F., Nakagawa T., Sato T., Sugihara K., Hoon D.S.B. (2011). Kinesin 18A expression: Clinical relevance to colorectal cancer progression. Int. J. Cancer.

[428] Zhang C., Zhu C., Chen H., Li L., Guo L., Jiang W., Lu S.H. (2010). Kif18A is involved in human breast carcinogenesis. Carcinogenesis.

[429] Rucksaken R., Khoontawad J., Roytrakul S., Pinlaor P., Hiraku Y., Wongkham C., Pairojkul C., Boonmars T., Pinlaor S. (2012). Proteomic analysis to identify plasma orosomucoid 2 and kinesin 18A as potential biomarkers of cholangiocarcinoma. Cancer Biomark..

[430] Tooker B.C., Newman L.S., Bowler R.P., Karjalainen A., Oksa P., Vainio H., Pukkala E., Brandt-Rauf P.W. (2011). Proteomic detection of cancer in asbestosis patients using SELDI-TOF discovered serum protein biomarkers. Biomarkers.

[431] Zhu H., Xu W., Zhang H., Liu J., Xu H., Lu S., Dang S., Kuang Y., Jin X., Wang Z. (2013). Targeted deletion of Kif18a protects from colitis-associated colorectal (CAC) tumors in mice through impairing Akt phosphorylation. Biochem. Biophys. Res. Commun..

[432] Kurasawa Y., Earnshaw W.C., Mochizuki Y., Dohmae N., Todokoro K. (2004). Essential roles of KIF4 and its binding partner PRC1 in organized central spindle midzone formation. EMBO J..

[433] Mazumdar M., Lee J.-H., Sengupta K., Ried T., Rane S., Misteli T. (2006). Tumor formation via loss of a molecular motor protein. Curr. Biol..

[434] Gao J., Sai N., Wang C., Sheng X., Shao Q., Zhou C., Shi Y., Sun S., Qu X., Zhu C. (2011). Overexpression of chromokinesin KIF4 inhibits proliferation of human gastric carcinoma cells both in vitro and in vivo. Tumour Biol..

[435] Narayan G., Bourdon V., Chaganti S., Arias-Pulido H., Nandula S.V., Rao P.H., Gissmann L., Dürst M., Schneider A., Pothuri B. (2007). Gene dosage alterations revealed by cDNA microarray analysis in cervical cancer: Identification of candidate amplified and overexpressed genes. Genes Chromosom. Cancer.

[436] Taniwaki M., Takano A., Ishikawa N., Yasui W., Inai K., Nishimura H., Tsuchiya E., Kohno N., Nakamura Y., Daigo Y. (2007). Activation of KIF4A as a prognostic biomarker and therapeutic target for lung cancer. Clin. Cancer Res..

[437] Jordan M.A., Wilson L. (2004). Microtubules as a target for anticancer drugs. Nat. Rev. Cancer.

[438] Gotaskie G.E., Andreassi B.F. (1994). Paclitaxel: A new antimitotic chemotherapeutic agent. Cancer Pract..

[439] Kavallaris M. (2010). Microtubules and resistance to tubulin-binding agents. Nat. Rev. Cancer.

[440] Zhou X.J., Rahmani R. (1992). Preclinical and clinical pharmacology of vinca alkaloids. Drugs.

[441] Jordan M.A., Toso R.J., Thrower D., Wilson L. (1993). Mechanism of mitotic block and inhibition of cell proliferation by taxol at low concentrations. Proc. Natl. Acad. Sci. USA.

[442] Jordan M.A., Wendell K., Gardiner S., Derry W.B., Copp H., Wilson L. (1996). Mitotic block induced in HeLa cells by low concentrations of paclitaxel (Taxol) results in abnormal mitotic exit and apoptotic cell death. Cancer Res..

[443] Jordan M.A., Thrower D., Wilson L. (1992). Effects of vinblastine, podophyllotoxin and nocodazole on mitotic spindles. Implications for the role of microtubule dynamics in mitosis. J. Cell Sci..

[444] Yang Z., Kenny A.E., Brito D.A., Rieder C.L. (2009). Cells satisfy the mitotic checkpoint in Taxol, and do so faster in concentrations that stabilize syntelic attachments. J. Cell Biol..

[445] Zasadil L.M., Andersen K.A., Yeum D., Rocque G.B., Wilke L.G., Tevaarwerk A.J., Raines R.T., Burkard M.E., Weaver B.A. (2014). Cytotoxicity of paclitaxel in breast cancer is due to chromosome missegregation on multipolar spindles. Sci. Transl. Med..

[446] Manchado E., Guillamot M., Malumbres M. (2012). Killing cells by targeting mitosis. Cell Death Differ..

[447] Miglarese M.R., Carlson R.O. (2006). Development of new cancer therapeutic agents targeting mitosis. Expert Opin. Investig. Drugs.

[448] Jackson J.R., Patrick D.R., Dar M.M., Huang P.S. (2007). Targeted anti-mitotic therapies: can we improve on tubulin agents?. Nat. Rev. Cancer.

[449] Wood K.W., Lad L., Luo L., Qian X., Knight S.D., Nevins N., Brejc K., Sutton D., Gilmartin A.G., Chua P.R. (2010). Antitumor activity of an allosteric inhibitor of centromere-associated protein-E. Proc. Natl. Acad. Sci. USA.

[450] Qian X., McDonald A., Zhou H.-J., Adams N.D., Parrish C.A., Duffy K.J., Fitch D.M., Tedesco R., Ashcraft L.W., Yao B. (2010). Discovery of the First Potent and Selective Inhibitor of Centromere-Associated Protein E: GSK923295. ACS Med. Chem. Lett..

[451] Balamuth N.J., Wood A., Wang Q., Jagannathan J., Mayes P., Zhang Z., Chen Z., Rappaport E., Courtright J., Pawel B. (2010). Serial transcriptome analysis and cross-species integration identifies centromere-associated protein E as a novel neuroblastoma target. Cancer Res..

[452] Hu Z., Kuo W.-l., Das D., Ziyad S., Gu S., Bhattacharya S., Wyrobek A., Wang N., Feiler H., Wooster R. (2009). Abstract #5572: Small molecular inhibitor of centromere-associated protein E (CENP-E), GSK923295A inhibits cell growth in breast cancer cells. Cancer Res..

[453] Lock R.B., Carol H., Morton C.L., Keir S.T., Reynolds C.P., Kang M.H., Maris J.M., Wozniak A.W., Gorlick R., Kolb E.A. (2012). Initial testing of the CENP-E inhibitor GSK923295A by the pediatric preclinical testing program. Pediatr. Blood Cancer.

[454] Mayes P.A., Degenhardt Y.Y., Wood A., Toporovskya Y., Diskin S.J., Haglund E., Moy C., Wooster R., Maris J.M. (2013). Mitogen-activated protein kinase (MEK/ERK) inhibition sensitizes cancer cells to centromere-associated protein E inhibition. Int. J. Cancer.

[455] Sutton D., Gilmartin A., Kusnierz A., Sung C.-M., Luo L., Carson J., Laquerre S., Cornwell W., King A., Knight S. (2007). A potent and selective inhibitor of the mitotic kinesin CENP-E (GSK923295A), demonstrates a novel mechanism of inhibiting tumor cell proliferation and shows activity against a broad panel of human tumor cell lines in vitro. Am. Assoc. Cancer Res..

[456] Bennett A., Bechi B., Tighe A., Thompson S., Procter D.J., Taylor S.S. (2015). Cenp-E inhibitor GSK923295: Novel synthetic route and use as a tool to generate aneuploidy. Oncotarget.

[457] Tcherniuk S.O., Oleinikov A.V. (2015). Pgp efflux pump decreases the cytostatic effect of CENP-E inhibitor GSK923295. Cancer Lett..

[458] Ohashi A., Ohori M., Iwai K., Nakayama Y., Nambu T., Morishita D., Kawamoto T., Miyamoto M., Hirayama T., Okaniwa M. (2015). Aneuploidy generates proteotoxic stress and DNA damage concurrently with p53-mediated post-mitotic apoptosis in SAC-impaired cells. Nat. Commun..

[459] Kim J.-H., Lee H.-S., Lee N.C.O., Goncharov N.V., Kumeiko V., Masumoto H., Earnshaw W.C., Kouprina N., Larionov V. (2016). Development of a novel HAC-based “gain of signal” quantitative assay for measuring chromosome instability (CIN) in cancer cells. Oncotarget.

[460] Henderson M.C., Shaw Y.-J.Y., Wang H., Han H., Hurley L.H., Flynn G., Dorr R.T., Von Hoff D.D. (2009). UA62784, a novel inhibitor of centromere protein E kinesin-like protein. Mol. Cancer Ther..

[461] Tcherniuk S., Deshayes S., Sarli V., Divita G., Abrieu A. (2011). UA62784 Is a cytotoxic inhibitor of microtubules, not CENP-E. Chem. Biol..

[462] Maiato H., Logarinho E. (2011). Motor-dependent and -independent roles of CENP-E at kinetochores: The cautionary tale of UA62784. Chem. Biol..

[463] Shaw A.Y., Henderson M.C., Flynn G., Samulitis B., Han H., Stratton S.P., Chow H.H.S., Hurley L.H., Dorr R.T. (2009). Characterization of novel diaryl oxazole-based compounds as potential agents to treat pancreatic cancer. J. Pharmacol. Exp. Ther..

[464] Ding X., Yan F., Yao P., Yang Z., Wan W., Wang X., Liu J., Gao X., Abrieu A., Zhu T. (2010). Probing CENP-E function in chromosome dynamics using small molecule inhibitor syntelin. Cell Res..

[465] Chung V., Heath E.I., Schelman W.R., Johnson B.M., Kirby L.C., Lynch K.M., Botbyl J.D., Lampkin T.A., Holen K.D. (2012). First-time-in-human study of GSK923295, a novel antimitotic inhibitor of centromere-associated protein E (CENP-E), in patients with refractory cancer. Cancer Chemother. Pharmacol..

[466] Capell B.C., Erdos M.R., Madigan J.P., Fiordalisi J.J., Varga R., Conneely K.N., Gordon L.B., Der C.J., Cox A.D., Collins F.S. (2005). Inhibiting farnesylation of progerin prevents the characteristic nuclear blebbing of Hutchinson-Gilford progeria syndrome. Proc. Natl. Acad. Sci. USA.

[467] Buckner F.S., Eastman R.T., Yokoyama K., Gelb M.H., Van Voorhis W.C. (2005). Protein farnesyl transferase inhibitors for the treatment of malaria and African trypanosomiasis. Curr. Opin. Investig. Drugs.

[468] Gordon L.B., Kleinman M.E., Miller D.T., Neuberg D.S., Giobbie-Hurder A., Gerhard-Herman M., Smoot L.B., Gordon C.M., Cleveland R., Snyder B.D. (2012). Clinical trial of a farnesyltransferase inhibitor in children with Hutchinson-Gilford progeria syndrome. Proc. Natl. Acad. Sci. USA.

[469] Nallan L., Bauer K.D., Bendale P., Rivas K., Yokoyama K., Horney C.P., Pendyala P.R., Floyd D., Lombardo L.J., Williams D.K. (2005). Protein farnesyltransferase inhibitors exhibit potent antimalarial activity. J. Med. Chem..

[470] Wiesner J., Kettler K., Sakowski J., Ortmann R., Katzin A.M., Kimura E.A., Silber K., Klebe G., Jomaa H., Schlitzer M. (2004). Farnesyltransferase inhibitors inhibit the growth of malaria parasites in vitro and in vivo. Angew. Chem. Int. Ed. Engl..

[471] Shen M., Pan P., Li Y., Li D., Yu H., Hou T. (2015). Farnesyltransferase and geranylgeranyltransferase I: Structures, mechanism, inhibitors and molecular modeling. Drug Discov. Today.

[472] Moorthy N.S., Sousa S.F., Ramos M.J., Fernandes P.A. (2013). Farnesyltransferase inhibitors: A comprehensive review based on quantitative structural analysis. Curr. Med. Chem..

[473] Agrawal A.G., Somani R.R. (2009). Farnesyltransferase inhibitor as anticancer agent. Mini Rev. Med. Chem..

[474] Kho Y., Kim S.C., Jiang C., Barma D., Kwon S.W., Cheng J., Jaunbergs J., Weinbaum C., Tamanoi F., Falck J. (2004). A tagging-via-substrate technology for detection and proteomics of farnesylated proteins. Proc. Natl. Acad. Sci. USA.

[475] Clark G.J., Kinch M.S., Rogers-Graham K., Sebti S.M., Hamilton A.D., Der C.J. (1997). The Ras-related protein Rheb is farnesylated and antagonizes Ras signaling and transformation. J. Biol. Chem..

[476] Holstein S.A., Hohl R.J. (2012). Is there a future for prenyltransferase inhibitors in cancer therapy?. Curr. Opin. Pharmacol..

[477] Kohl N.E., Mosser S.D., deSolms S.J., Giuliani E.A., Pompliano D.L., Graham S.L., Smith R.L., Scolnick E.M., Oliff A., Gibbs J.B. (1993). Selective inhibition of ras-dependent transformation by a farnesyltransferase inhibitor. Science.

[478] Lee K.H., Koh M., Moon A. (2016). Farnesyl transferase inhibitor FTI-277 inhibits breast cell invasion and migration by blocking H-Ras activation. Oncol. Lett..

[479] Cox A.D., Der C.J. (1997). Farnesyltransferase inhibitors and cancer treatment: Targeting simply Ras?. Biochim. Biophys. Acta.

[480] Sepp-Lorenzino L., Ma Z., Rands E., Kohl N.E., Gibbs J.B., Oliff A., Rosen N. (1995). A peptidomimetic inhibitor of farnesyl:protein transferase blocks the anchorage-dependent and -independent growth of human tumor cell lines. Cancer Res..

[481] Karp J.E., Lancet J.E., Kaufmann S.H., End D.W., Wright J.J., Bol K., Horak I., Tidwell M.L., Liesveld J., Kottke T.J. (2001). Clinical and biologic activity of the farnesyltransferase inhibitor R115777 in adults with refractory and relapsed acute leukemias: A phase 1 clinical-laboratory correlative trial. Blood.

[482] Rolland D., Camara-Clayette V., Barbarat A., Salles G., Coiffier B., Ribrag V., Thieblemont C. (2008). Farnesyltransferase inhibitor R115777 inhibits cell growth and induces apoptosis in mantle cell lymphoma. Cancer Chemother. Pharmacol..

[483] Adjei A.A., Davis J.N., Bruzek L.M., Erlichman C., Kaufmann S.H. (2001). Synergy of the protein farnesyltransferase inhibitor SCH66336 and cisplatin in human cancer cell lines. Clin. Cancer Res..

[484] Russo P., Malacarne D., Falugi C., Trombino S., O’Connor P.M. (2002). RPR-115135, a farnesyltransferase inhibitor, increases 5-FU- cytotoxicity in ten human colon cancer cell lines: role of p53. Int. J. Cancer.

[485] Brassard D.L., English J.M., Malkowski M., Kirschmeier P., Nagabhushan T.L., Bishop W.R. (2002). Inhibitors of farnesyl protein transferase and MEK1,2 induce apoptosis in fibroblasts transformed with farnesylated but not geranylgeranylated H-Ras. Exp. Cell Res..

[486] Edamatsu H., Gau C.L., Nemoto T., Guo L., Tamanoi F. (2000). Cdk inhibitors, roscovitine and olomoucine, synergize with farnesyltransferase inhibitor (FTI) to induce efficient apoptosis of human cancer cell lines. Oncogene.

[487] Nagai T., Ohmine K., Fujiwara S., Uesawa M., Sakurai C., Ozawa K. (2010). Combination of tipifarnib and rapamycin synergistically inhibits the growth of leukemia cells and overcomes resistance to tipifarnib via alteration of cellular signaling pathways. Leuk. Res..

[488] Moasser M.M., Sepp-Lorenzino L., Kohl N.E., Oliff A., Balog A., Su D.S., Danishefsky S.J., Rosen N. (1998). Farnesyl transferase inhibitors cause enhanced mitotic sensitivity to taxol and epothilones. Proc. Natl. Acad. Sci. USA.

[489] Karp J.E., Kaufmann S.H., Adjei A.A., Lancet J.E., Wright J.J., End D.W. (2001). Current status of clinical trials of farnesyltransferase inhibitors. Curr. Opin. Oncol..

[490] Santos E.S., Rosenblatt J.D., Goodman M. (2004). Role of farnesyltransferase inhibitors in hematologic malignancies. Expert Rev. Anticancer Ther..

[491] Sebti S.M., Adjei A.A. (2004). Farnesyltransferase inhibitors. Semin. Oncol..

[492] Karp J.E., Lancet J.E. (2005). Targeting the process of farynesylation for therapy of hematologic malignancies. Curr. Mol. Med..

[493] Epling-Burnette P.K., Loughran T.P. (2010). Suppression of farnesyltransferase activity in acute myeloid leukemia and myelodysplastic syndrome: Current understanding and recommended use of tipifarnib. Expert Opin. Investig. Drugs.

[494] Rao S., Cunningham D., de Gramont A., Scheithauer W., Smakal M., Humblet Y., Kourteva G., Iveson T., Andre T., Dostalova J. (2004). Phase III double-blind placebo-controlled study of farnesyl transferase inhibitor R115777 in patients with refractory advanced colorectal cancer. J. Clin. Oncol..

[495] Van Cutsem E., van de Velde H., Karasek P., Oettle H., Vervenne W.L., Szawlowski A., Schoffski P., Post S., Verslype C., Neumann H. (2004). Phase III trial of gemcitabine plus tipifarnib compared with gemcitabine plus placebo in advanced pancreatic cancer. J. Clin. Oncol..

[496] Macdonald J.S., McCoy S., Whitehead R.P., Iqbal S., Wade J.L., Giguere J.K., Abbruzzese J.L. (2005). A phase II study of farnesyl transferase inhibitor R115777 in pancreatic cancer: A Southwest oncology group (SWOG 9924) study. Investig. New Drugs.

[497] Harousseau J.L., Martinelli G., Jedrzejczak W.W., Brandwein J.M., Bordessoule D., Masszi T., Ossenkoppele G.J., Alexeeva J.A., Beutel G., Maertens J. (2009). A randomized phase 3 study of tipifarnib compared with best supportive care, including hydroxyurea, in the treatment of newly diagnosed acute myeloid leukemia in patients 70 years or older. Blood.

[498] Stieglitz E., Ward A.F., Gerbing R.B., Alonzo T.A., Arceci R.J., Liu Y.L., Emanuel P.D., Widemann B.C., Cheng J.W., Jayaprakash N. (2015). Phase II/III trial of a pre-transplant farnesyl transferase inhibitor in juvenile myelomonocytic leukemia: A report from the Children’s Oncology Group. Pediatr. Blood Cancer.

[499] Gajewski T.F., Salama A.K., Niedzwiecki D., Johnson J., Linette G., Bucher C., Blaskovich M.A., Sebti S.M., Haluska F., Cancer and Leukemia Group B (2012). Phase II study of the farnesyltransferase inhibitor R115777 in advanced melanoma (CALGB 500104). J. Transl. Med..

[500] Burnett A.K., Russell N.H., Culligan D., Cavanagh J., Kell J., Wheatley K., Virchis A., Hills R.K., Milligan D., AML Working Group of the UK National Cancer Research Institute (2012). The addition of the farnesyl transferase inhibitor, tipifarnib, to low dose cytarabine does not improve outcome for older patients with AML. Br. J. Haematol..

[501] Meier W., du Bois A., Rau J., Gropp-Meier M., Baumann K., Huober J., Wollschlaeger K., Kreienberg R., Canzler U., Schmalfeldt B. (2012). Randomized phase II trial of carboplatin and paclitaxel with or without lonafarnib in first-line treatment of epithelial ovarian cancer stage IIB-IV. Gynecol. Oncol..

[502] Adjei A.A., Croghan G.A., Erlichman C., Marks R.S., Reid J.M., Sloan J.A., Pitot H.C., Alberts S.R., Goldberg R.M., Hanson L.J. (2003). A Phase I trial of the farnesyl protein transferase inhibitor R115777 in combination with gemcitabine and cisplatin in patients with advanced cancer. Clin. Cancer Res..

[503] Siegel-Lakhai W.S., Crul M., Zhang S., Sparidans R.W., Pluim D., Howes A., Solanki B., Beijnen J.H., Schellens J.H. (2005). Phase I and pharmacological study of the farnesyltransferase inhibitor tipifarnib (Zarnestra, R115777) in combination with gemcitabine and cisplatin in patients with advanced solid tumours. Br. J. Cancer.

[504] Sparano J.A., Moulder S., Kazi A., Vahdat L., Li T., Pellegrino C., Munster P., Malafa M., Lee D., Hoschander S. (2006). Targeted inhibition of farnesyltransferase in locally advanced breast cancer: A phase I and II trial of tipifarnib plus dose-dense doxorubicin and cyclophosphamide. J. Clin. Oncol..

[505] Medeiros B.C., Landau H.J., Morrow M., Lockerbie R.O., Pitts T., Eckhardt S.G. (2007). The farnesyl transferase inhibitor, tipifarnib, is a potent inhibitor of the MDR1 gene product, P-glycoprotein, and demonstrates significant cytotoxic synergism against human leukemia cell lines. Leukemia.

[506] Jabbour E., Kantarjian H., Ravandi F., Garcia-Manero G., Estrov Z., Verstovsek S., O’Brien S., Faderl S., Thomas D.A., Wright J.J. (2011). A phase 1–2 study of a farnesyltransferase inhibitor, tipifarnib, combined with idarubicin and cytarabine for patients with newly diagnosed acute myeloid leukemia and high-risk myelodysplastic syndrome. Cancer.

[507] Li T., Guo M., Gradishar W.J., Sparano J.A., Perez E.A., Wang M., Sledge G.W. (2012). A phase II trial of capecitabine in combination with the farnesyltransferase inhibitor tipifarnib in patients with anthracycline-treated and taxane-resistant metastatic breast cancer: An Eastern Cooperative Oncology Group Study (E1103). Breast Cancer Res. Treat..

[508] Kim E.S., Kies M.S., Fossella F.V., Glisson B.S., Zaknoen S., Statkevich P., Munden R.F., Summey C., Pisters K.M., Papadimitrakopoulou V. (2005). Phase II study of the farnesyltransferase inhibitor lonafarnib with paclitaxel in patients with taxane-refractory/resistant nonsmall cell lung carcinoma. Cancer.

[509] Karp J.E., Smith B.D., Gojo I., Lancet J.E., Greer J., Klein M., Morris L., Levis M.J., Gore S.D., Wright J.J. (2008). Phase II trial of tipifarnib as maintenance therapy in first complete remission in adults with acute myelogenous leukemia and poor-risk features. Clin. Cancer Res..

[510] Castro-Castro A., Janke C., Montagnac G., Paul-Gilloteaux P., Chavrier P. (2012). ATAT1/MEC-17 acetyltransferase and HDAC6 deacetylase control a balance of acetylation of alpha-tubulin and cortactin and regulate MT1-MMP trafficking and breast tumor cell invasion. Eur. J. Cell Biol..

[511] Boggs A.E., Vitolo M.I., Whipple R.A., Charpentier M.S., Goloubeva O.G., Ioffe O.B., Tuttle K.C., Slovic J., Lu Y., Mills G.B. (2015). α-Tubulin acetylation elevated in metastatic and basal-like breast cancer cells promotes microtentacle formation, adhesion, and invasive migration. Cancer Res..

[512] Kashiwaya K., Nakagawa H., Hosokawa M., Mochizuki Y., Ueda K., Piao L., Chung S., Hamamoto R., Eguchi H., Ohigashi H. (2010). Involvement of the tubulin tyrosine ligase-like family member 4 polyglutamylase in PELP1 polyglutamylation and chromatin remodeling in pancreatic cancer cells. Cancer Res..

[513] Wasylyk C., Zambrano A., Zhao C., Brants J., Abecassis J., Schalken J.A., Rogatsch H., Schaefer G., Pycha A., Klocker H. (2010). Tubulin tyrosine ligase like 12 links to prostate cancer through tubulin posttranslational modification and chromosome ploidy. Int. J. Cancer.

[514] Brants J., Semenchenko K., Wasylyk C., Robert A., Carles A., Zambrano A., Pradeau-Aubreton K., Birck C., Schalken J.A., Poch O. (2012). Tubulin tyrosine ligase like 12, a TTLL family member with SET- and TTL-like domains and roles in histone and tubulin modifications and mitosis. PLoS ONE.

[515] Rocha C., Papon L., Cacheux W., Marques Sousa P., Lascano V., Tort O., Giordano T., Vacher S., Lemmers B., Mariani P. (2014). Tubulin glycylases are required for primary cilia, control of cell proliferation and tumor development in colon. EMBO J..

[516] Lafanechere L., Courtay-Cahen C., Kawakami T., Jacrot M., Rudiger M., Wehland J., Job D., Margolis R.L. (1998). Suppression of tubulin tyrosine ligase during tumor growth. J. Cell Sci..

[517] Mialhe A., Lafanechere L., Treilleux I., Peloux N., Dumontet C., Bremond A., Panh M.H., Payan R., Wehland J., Margolis R.L. (2001). Tubulin detyrosination is a frequent occurrence in breast cancers of poor prognosis. Cancer Res..

[518] Kato C., Miyazaki K., Nakagawa A., Ohira M., Nakamura Y., Ozaki T., Imai T., Nakagawara A. (2004). Low expression of human tubulin tyrosine ligase and suppressed tubulin tyrosination/detyrosination cycle are associated with impaired neuronal differentiation in neuroblastomas with poor prognosis. Int. J. Cancer.

[519] Soucek K., Kamaid A., Phung A.D., Kubala L., Bulinski J.C., Harper R.W., Eiserich J.P. (2006). Normal and prostate cancer cells display distinct molecular profiles of alpha-tubulin posttranslational modifications. Prostate.

[520] Kuroda H., Saito K., Kuroda M., Suzuki Y. (2010). Differential expression of glu-tubulin in relation to mammary gland disease. Virchows Arch..

[521] Whipple R.A., Matrone M.A., Cho E.H., Balzer E.M., Vitolo M.I., Yoon J.R., Ioffe O.B., Tuttle K.C., Yang J., Martin S.S. (2010). Epithelial-to-mesenchymal transition promotes tubulin detyrosination and microtentacles that enhance endothelial engagement. Cancer Res..

[522] Kreuger M.R., Grootjans S., Biavatti M.W., Vandenabeele P., D’Herde K. (2012). Sesquiterpene lactones as drugs with multiple targets in cancer treatment: Focus on parthenolide. Anticancer Drugs.

[523] Curry E.A., Murry D.J., Yoder C., Fife K., Armstrong V., Nakshatri H., O’Connell M., Sweeney C.J. (2004). Phase I dose escalation trial of feverfew with standardized doses of parthenolide in patients with cancer. Investig. New Drugs.

[524] Ghantous A., Sinjab A., Herceg Z., Darwiche N. (2013). Parthenolide: From plant shoots to cancer roots. Drug Discov. Today.

[525] Bork P.M., Schmitz M.L., Kuhnt M., Escher C., Heinrich M. (1997). Sesquiterpene lactone containing Mexican Indian medicinal plants and pure sesquiterpene lactones as potent inhibitors of transcription factor NF-κB. FEBS Lett..

[526] Hehner S.P., Hofmann T.G., Droge W., Schmitz M.L. (1999). The antiinflammatory sesquiterpene lactone parthenolide inhibits NF-κB by targeting the IκB kinase complex. J. Immunol..

[527] Kwok B.H., Koh B., Ndubuisi M.I., Elofsson M., Crews C.M. (2001). The anti-inflammatory natural product parthenolide from the medicinal herb Feverfew directly binds to and inhibits IκB kinase. Chem. Biol..

[528] Garcia-Pineres A.J., Castro V., Mora G., Schmidt T.J., Strunck E., Pahl H.L., Merfort I. (2001). Cysteine 38 in p65/NF-κB plays a crucial role in DNA binding inhibition by sesquiterpene lactones. J. Biol. Chem..

[529] Fonrose X., Ausseil F., Soleilhac E., Masson V., David B., Pouny I., Cintrat J.C., Rousseau B., Barette C., Massiot G. (2007). Parthenolide inhibits tubulin carboxypeptidase activity. Cancer Res..

[530] Whipple R.A., Vitolo M.I., Boggs A.E., Charpentier M.S., Thompson K., Martin S.S. (2013). Parthenolide and costunolide reduce microtentacles and tumor cell attachment by selectively targeting detyrosinated tubulin independent from NF-κB inhibition. Breast Cancer Res..

[531] Shanmugam R., Kusumanchi P., Appaiah H., Cheng L., Crooks P., Neelakantan S., Peat T., Klaunig J., Matthews W., Nakshatri H. (2011). A water soluble parthenolide analog suppresses in vivo tumor growth of two tobacco-associated cancers, lung and bladder cancer, by targeting NF-κB and generating reactive oxygen species. Int. J. Cancer.

[532] Shanmugam R., Kusumanchi P., Appaiah H., Cheng L., Crooks P., Neelakantan S., Peat T., Klaunig J., Matthews W., Nakshatri H. (2010). Naturally occurring asteriscunolide A induces apoptosis and activation of mitogen-activated protein kinase pathway in human tumor cell lines. Mol. Carcinog..

[533] Rozenblat S., Grossman S., Bergman M., Gottlieb H., Cohen Y., Dovrat S. (2008). Induction of G2/M arrest and apoptosis by sesquiterpene lactones in human melanoma cell lines. Biochem. Pharmacol..

[534] Guzman M.L., Rossi R.M., Karnischky L., Li X., Peterson D.R., Howard D.S., Jordan C.T. (2005). The sesquiterpene lactone parthenolide induces apoptosis of human acute myelogenous leukemia stem and progenitor cells. Blood.

[535] Carnero A., Garcia-Mayea Y., Mir C., Lorente J., Rubio I.T., ME L.L. (2016). The cancer stem-cell signaling network and resistance to therapy. Cancer Treat. Rev..

[536] Valent P., Bonnet D., De Maria R., Lapidot T., Copland M., Melo J.V., Chomienne C., Ishikawa F., Schuringa J.J., Stassi G. (2012). Cancer stem cell definitions and terminology: The devil is in the details. Nat. Rev. Cancer.

[537] Kawasaki B.T., Hurt E.M., Kalathur M., Duhagon M.A., Milner J.A., Kim Y.S., Farrar W.L. (2009). Effects of the sesquiterpene lactone parthenolide on prostate tumor-initiating cells: An integrated molecular profiling approach. Prostate.

[538] Guzman M.L., Rossi R.M., Neelakantan S., Li X., Corbett C.A., Hassane D.C., Becker M.W., Bennett J.M., Sullivan E., Lachowicz J.L. (2007). An orally bioavailable parthenolide analog selectively eradicates acute myelogenous leukemia stem and progenitor cells. Blood.

[539] Carlisi D., Buttitta G., Di Fiore R., Scerri C., Drago-Ferrante R., Vento R., Tesoriere G. (2016). Parthenolide and DMAPT exert cytotoxic effects on breast cancer stem-like cells by inducing oxidative stress, mitochondrial dysfunction and necrosis. Cell Death Dis..

[540] Shanmugam R., Kusumanchi P., Cheng L., Crooks P., Neelakantan S., Matthews W., Nakshatri H., Sweeney C.J. (2010). A water-soluble parthenolide analogue suppresses in vivo prostate cancer growth by targeting NFkappaB and generating reactive oxygen species. Prostate.

[541] Sweeney C.J., Mehrotra S., Sadaria M.R., Kumar S., Shortle N.H., Roman Y., Sheridan C., Campbell R.A., Murry D.J., Badve S. (2005). The sesquiterpene lactone parthenolide in combination with docetaxel reduces metastasis and improves survival in a xenograft model of breast cancer. Mol. Cancer Ther..

[542] Zhang D., Qiu L., Jin X., Guo Z., Guo C. (2009). Nuclear factor-kappaB inhibition by parthenolide potentiates the efficacy of Taxol in non-small cell lung cancer in vitro and in vivo. Mol. Cancer Res..

[543] Liu Y., Lu W.L., Guo J., Du J., Li T., Wu J.W., Wang G.L., Wang J.C., Zhang X., Zhang Q. (2008). A potential target associated with both cancer and cancer stem cells: A combination therapy for eradication of breast cancer using vinorelbine stealthy liposomes plus parthenolide stealthy liposomes. J. Control. Release.

